# Inhibition of 26S proteasome activity by α‐synuclein is mediated by the proteasomal chaperone Rpn14/PAAF1

**DOI:** 10.1111/acel.14128

**Published:** 2024-02-28

**Authors:** Dajana Galka, Tariq T. Ali, Alexander Bast, Marie Niederleithinger, Ellen Gerhardt, Ryo Motosugi, Eri Sakata, Michael Knop, Tiago F. Outeiro, Blagovesta Popova, Gerhard H. Braus

**Affiliations:** ^1^ Department of Molecular Microbiology and Genetics, Institute for Microbiology and Genetics University of Göttingen Göttingen Germany; ^2^ Department of Experimental Neurodegeneration, Center for Biostructural Imaging of Neurodegeneration University Medical Center Göttingen Göttingen Germany; ^3^ Institute for Auditory Neuroscience University Medical Center Göttingen Göttingen Germany; ^4^ Multiscale Bioimaging: from Molecular Machines to Networks of Excitable Cells (MBExC) University of Göttingen Göttingen Germany; ^5^ Zentrum für Molekulare Biologie der Universität Heidelberg (ZMBH), DKFZ‐ZMBH Alliance Heidelberg University Heidelberg Germany; ^6^ Translational and Clinical Research Institute, Faculty of Medical Sciences Newcastle University Newcastle Upon Tyne UK; ^7^ Max Planck Institute for Multidisciplinary Sciences Göttingen Germany; ^8^ Scientific employee with an honorary contract at Deutsches Zentrum für Neurodegenerative Erkrankungen (DZNE) Göttingen Germany

**Keywords:** 26S proteasome, α‐Synuclein, Parkinson's disease, posttranslational modifications, proteasomal chaperone, protein homeostasis, tandem fluorescent protein timer, yeast

## Abstract

Parkinson's disease (PD) is characterized by aggregation of α‐synuclein (α‐syn) into protein inclusions in degenerating brains. Increasing amounts of aggregated α‐syn species indicate significant perturbation of cellular proteostasis. Altered proteostasis depends on α‐syn protein levels and the impact of α‐syn on other components of the proteostasis network. Budding yeast *Saccharomyces cerevisiae* was used as eukaryotic reference organism to study the consequences of α‐syn expression on protein dynamics. To address this, we investigated the impact of overexpression of α‐syn and S129A variant on the abundance and stability of most yeast proteins using a genome‐wide yeast library and a tandem fluorescent protein timer (tFT) reporter as a measure for protein stability. This revealed that the stability of in total 377 cellular proteins was altered by α‐syn expression, and that the impact on protein stability was significantly enhanced by phosphorylation at Ser129 (pS129). The proteasome assembly chaperone Rpn14 was identified as one of the top candidates for increased protein stability by expression of pS129 α‐syn. Elevated levels of Rpn14 enhanced the growth inhibition by α‐syn and the accumulation of ubiquitin conjugates in the cell. We found that Rpn14 interacts physically with α‐syn and stabilizes pS129 α‐syn. The expression of α‐syn along with elevated levels of Rpn14 or its human counterpart PAAF1 reduced the proteasome activity in yeast and in human cells, supporting that pS129 α‐syn negatively affects the 26S proteasome through Rpn14. This comprehensive study into the alternations of protein homeostasis highlights the critical role of the Rpn14/PAAF1 in α‐syn‐mediated proteasome dysfunction.

## Graphical abstract

α‐Synuclein (α‐syn) is a hallmark protein of Parkinson's disease. It affects multiple protein stabilities disturbing cellular proteostasis. α‐Synuclein interacts in yeast with the proteasomal chaperone Rpn14, which corresponds to mammalian PAAF1. Activities of the 26S proteasome are inhibited by α‐syn through Rpn14/PAAF1. This impact on proteostasis strongly correlates with α‐syn phosphorylation at S129. Rpn14/PAAF1 is a newly identified key factor functioning as link between α‐syn and cytotoxicity based on disturbances in cellular proteostasis as it is found in Parkinson's disease.

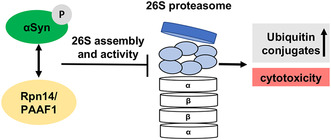

**Abbreviations**
BiFC: Bimolecular Fluorescence Complementation assays
CP: core particle
LBs: Lewy bodies
PI: Propidium iodide
PTMs: posttranslational modifications
RFU: relative fluorescence units
RP: regulatory particle
SC: synthetic complete dropout
SGA: Synthetic genetic array
tFT: tandem fluorescent protein timer
UPS: ubiquitin–proteasome system
Y2H: yeast‐two‐hybrid
yTHC: yeast *Tet*‐Promoters Hughes Collection

## 1 INTRODUCTION

Parkinson's disease (PD) is characterized by progressive degeneration of neuronal cells in the brain and, in most cases, by the presence of Lewy bodies (LBs) and Lewy neurites, protein inclusions rich in the protein α‐synuclein (α‐syn) (Spillantini et al., 1997). A large fraction of α‐syn in LB is phosphorylated at S129 (pS129) which indicates the important role of this modification for PD pathology (Oueslati, 2016). Accumulation of misfolded α‐syn indicates a failure of the proteostasis network, which is responsible for maintaining protein quality control (Lehtonen et al., 2019). The ubiquitin–proteasome system (UPS) and autophagy are the main protein clearance pathways in cells that execute cellular proteolysis and ensure the removal of dysfunctional proteins. Proteome balance declines with age and is linked to neurodegenerative diseases like PD. UPS and autophagy are responsible for degradation of α‐syn, and failure in either pathway leads to α‐syn accumulation and worsens the disease (Stefanis et al., 2019). A wide range of studies show that accumulation of misfolded α‐syn is associated with reduced proteasome activity, contributing to PD pathology (Bentea et al., 2017; McNaught et al., 2002; McNaught & Jenner, 2001). Variant (Stefanis et al., 2001; Tanaka et al., 2001), oligomeric (Emmanouilidou et al., 2010; Zhang et al., 2008), or aggregated (Snyder et al., 2003) forms of α‐syn can bind to and inhibit the proteasome. Cellular models of PD with α‐syn overexpression exhibit accumulation of ubiquitin conjugates and decreased proteasome function (Outeiro & Lindquist, 2003). The exact causes of proteasome inhibition in PD are not yet understood, but it is suggested that α‐syn disrupts UPS function, leading to imbalances in cellular proteostasis. However, a comprehensive study on PD‐related alterations of protein dynamics is still lacking.

The proteotoxicity of α‐syn is influenced by its turnover, which is regulated by various posttranslational modifications (PTMs) such as phosphorylation, nitration, sumoylation, ubiquitination, or acetylation. PTMs of α‐syn guide the protein into specific degradation pathways and regulate its clearance (Stefanis et al., 2019). Among these PTMs, phosphorylation at S129 plays a central role in protein stability and toxicity (Oueslati, 2016). It enhances α‐syn degradation by the 26S proteasome as well as by autophagy (Shahpasandzadeh et al., 2014; Tenreiro et al., 2014). Quantitative cellular proteomics revealed that α‐syn expression significantly changes the yeast proteome, leading to a decrease in the abundance of multiple 26S proteasome subunits (Popova, Galka, et al., 2021). This effect correlates with α‐syn turnover including phosphorylation of α‐syn at S129.

The yeast *Saccharomyces cerevisiae* was used as prototypic eukaryotic cell model to investigate the effects of α‐syn or the phosphorylation‐deficient variant S129A on protein homeostasis. Similar to neurons, α‐syn expression in yeast leads to the formation of inclusions and significant growth retardation (Outeiro & Lindquist, 2003; Petroi et al., 2012). A proteome‐wide screening using tandem fluorescent protein timer (tFT) fusions was conducted to explore changes in protein stability (Khmelinskii et al., 2012, 2014). This approach monitors changes in protein homeostasis beyond the level of protein abundance, providing insights into protein age. Multiple proteins with significantly changed stability were identified, highlighting for the first time the critical role of the proteasomal chaperone Rpn14 in α‐syn‐mediated alternations of cellular proteostasis. These findings provide novel insights into the complex interplay between the 26S proteasome and α‐syn causing a substantial disbalance in protein homeostasis.

## 2 EXPERIMENTAL PROCEDURES

Yeast strains and plasmids used in the study are listed in Tables S1 and S2.

### 2.1 Transformations and growth conditions

*Saccharomyces cerevisiae* strains were transformed using standard lithium acetate protocol (Gietz & Woods, 2002). Yeast strains were grown at 30°C in nonselective YEPD (Yeast Extract‐Peptone‐Dextrose) or synthetic complete dropout (SC) medium lacking the relevant amino acids for selection, supplemented with 2% glucose, 2% raffinose, or 2% galactose. The expression of essential genes from the yeast *Tet*‐Promoters Hughes Collection (yTHC) was downregulated by supplementing the medium with 10 μg/mL doxycycline. Expression of *GAL1*‐α‐syn was induced by shifting overnight cultures from 2% raffinose to 2% galactose‐containing SC selection medium at *A*_600_ = 0.3. Human Embryonic Kidney 293 (HEK) cells were maintained and transfected as previously described (Popova, Wang, et al., 2021).

### 2.2 Cloning of recombinant DNA

Yeast plasmids were constructed using GeneArt® Seamless Cloning and Assembly Enzyme Mix (Invitrogen, USA). The S129A‐VC and S129D‐VC mutants constructs were generated by site‐directed mutagenesis using QuikChange II Site‐Directed Mutagenesis Kit (Agilent Technologies). All constructs were verified by DNA sequencing.

### 2.3 Tandem fluorescent protein timer screening and data analyses

Yeast tFT library was employed that consists of 4044 strains each expressing a different tFT‐tagged protein (Khmelinskii et al., 2014). The generation of new libraries expressing α‐syn or S129A and the screening procedures were performed as in (Khmelinskii et al., 2014) and described briefly in Supplement Methods S1.

### 2.4 Spotting assay

Yeast cells were pre‐grown in selective SC medium containing 2% raffinose. After normalizing the cells to equal densities (*A*_600_ = 0.1), a series of 10‐fold dilutions were prepared and spotted in a volume of 10 μL onto selective SC agar plates supplemented with 2% glucose or 2% galactose. Where indicated, the plates were supplemented with 10 μg/mL doxycycline. The growth rate intensity was documented after 3 days of incubation at 30°C, unless indicated otherwise.

### 2.5 Yeast‐two‐hybrid assay

Protein–protein interactions were analyzed with yeast‐two‐hybrid (Y2H) assay by fusing two proteins of interest to the activation domain or the DNA‐binding domain of a transcriptional activator of a reporter gene as described (Golemis et al., 1999). α‐Synuclein, S129A or S129D were fused to the “acid blob” B42 as activation domain, whereas Rpn14 was fused to the DNA binding domain of the bacterial repressor protein LexA. The bait and the prey constructs were co‐transformed in the yeast strain EGY48. Interaction of the bait and prey fusion constructs was confirmed by growth on selective SC‐His‐Trp‐Leu medium complemented with 2% galactose.

### 2.6 Purification of 26S proteasomes

The intact 26S proteasomes were purified via *RPN11‐3xFLAG* tag, as described previously (Eisele et al., 2018). Details are described in Supplement Methods S1.

### 2.7 Western blot analysis

Protein extraction was performed as previously described (Knop et al., 1999). Western blot analysis was performed using standard procedures (Popova, Wang, et al., 2021). Following primary antibodies were used: α‐syn mouse antibody (1:2000; BD Transduction Laboratory, USA), mouse anti phospho‐Ser129 α‐syn antibody (1:2000; Wako Chemicals, USA), mouse anti‐ubiquitin antibody (1:2000; Merch Millipore, USA), rat anti‐GFP antibody (1:1000; Chromotek, Germany), mouse anti‐GAPDH antibody (1:5000; ThermoFisher Scientific, USA), and mouse anti His6 antibody (1:1000; ThermoFisher Scientific, USA). Western blot quantifications of pixel density values were obtained from TIFF files generated from digitized x‐ray films (Kodak, USA), and analyzed with ImageJ software (NIH, Bethesda, USA). Sample density values are presented as ratios to the corresponding loading control and normalized to the control per blot. At least three independent experiments were performed for quantification of the signals.

### 2.8 Measurement of peptidase activity

α‐Synuclein expression was induced for 16 h in SC selection medium supplemented with 2% galactose. Yeast cells were lysed with glass beads in A‐buffer (50 mM Tris–HCl [pH 7.5], 100 mM NaCl, 2 mM DTT, 2 mM ATP, 5 mM MgCl_2_). HEK cells were lysed by sonication in lysis buffer (40 mM Tris–HCl [pH 7.2], 50 mM NaCl, 2 mM BME, 2 mM ATP, 5 mM MgCl_2_, 10% glycerol). The protein extracts were cleared by centrifugation at 4°C at 15,000 *g* for 15 min. Protein concentration was determined with Bradford assay and a total of 60 μg crude protein extract from each probe was used for activity assays. Peptidase activity was measured using the fluorescent peptide substrate Suc‐LLVY‐AMC (Enzo Life Science, USA) at a final concentration of 100 μM in 20 mM Tris–HCl [pH 7.5], 50 mM NaCl, 2 mM DTT. The degradation of the fluorogenic peptide was measured by monitoring the fluorescence of the liberated 7‐amino‐4‐methylcoumarin (AMC) using TECAN Infinite 200 microplate reader (Tecan, Switzerland) at 37°C for 30 min (*excitation wavelength =* 350 nm; *emission wavelength =* 440 nm).

### 2.9 Native PAGE and fluorescence imaging

Yeast cells were cultured as described above. Cell pellets were frozen in liquid nitrogen and ground to powder in a mortar. Cell powder was resuspended in 1.5 volume of Extraction buffer (50 mM Tris pH 7.5, 100 mM NaCl, 10% glycerol, 10 mM MgCl_2_, 4 mM ATP). The protein extracts were cleared by centrifugation at 4°C at 15,000 *g* for 15 min. The concentration of the supernatants was determined by Bradford assay and 200 μg from each probe were loaded onto 4% native polyacrylamide gels, complemented with 1 mM ATP, 5 mM MgCl_2_ and 2.5% sucrose with a 3% stacking gel. Electrophoresis was run at 100 V, 4°C about 2.5 h–3 h. In‐gel peptidase activity assays were performed by overlaying the gels with 50 mM Tris–HCl pH 7.5, 150 mM NaCl, 5 mM MgCl_2_, 1 mM ATP, 50 μM Suc‐LLVY‐AMC for 10 min, followed by overlay with the same buffer supplemented with 0.05% SDS. Fluorescence was imaged with Fusion FX6 Edge Imaging System (Vilber, France).

### 2.10 Fluorescence microscopy and flow cytometry

Fluorescence images were acquired using a Zeiss Axio Observer microscope at 63× magnification. Z1 microscope (Zeiss, Germany) equipped with a CSU‐X1 A1 confocal scanner unit (Yokogawa, Japan), a QuantEM:512SC digital camera (Photometrics, USA), and SlideBook 6.0 software package (Intelligent Imaging Innovations, USA). Fluorescence measurements were performed after subtraction of the background fluorescence. Flow cytometry was performed as previously described (Popova, Galka, et al., 2021).

### 2.11 Quantification and statistical analysis

The data were analyzed with GraphPad Prism 6 software (San Diego, USA) and presented as means ± SEM from at least three independent experiments. The significance of differences was calculated using Student's *t* test or one‐way ANOVA. *p‐*Value <0.05 was considered to indicate a significant difference.

## 3 RESULTS

### 3.1 Expression of p129 α‐syn alters stability of numerous individual yeast proteins

**FIGURE 1:**
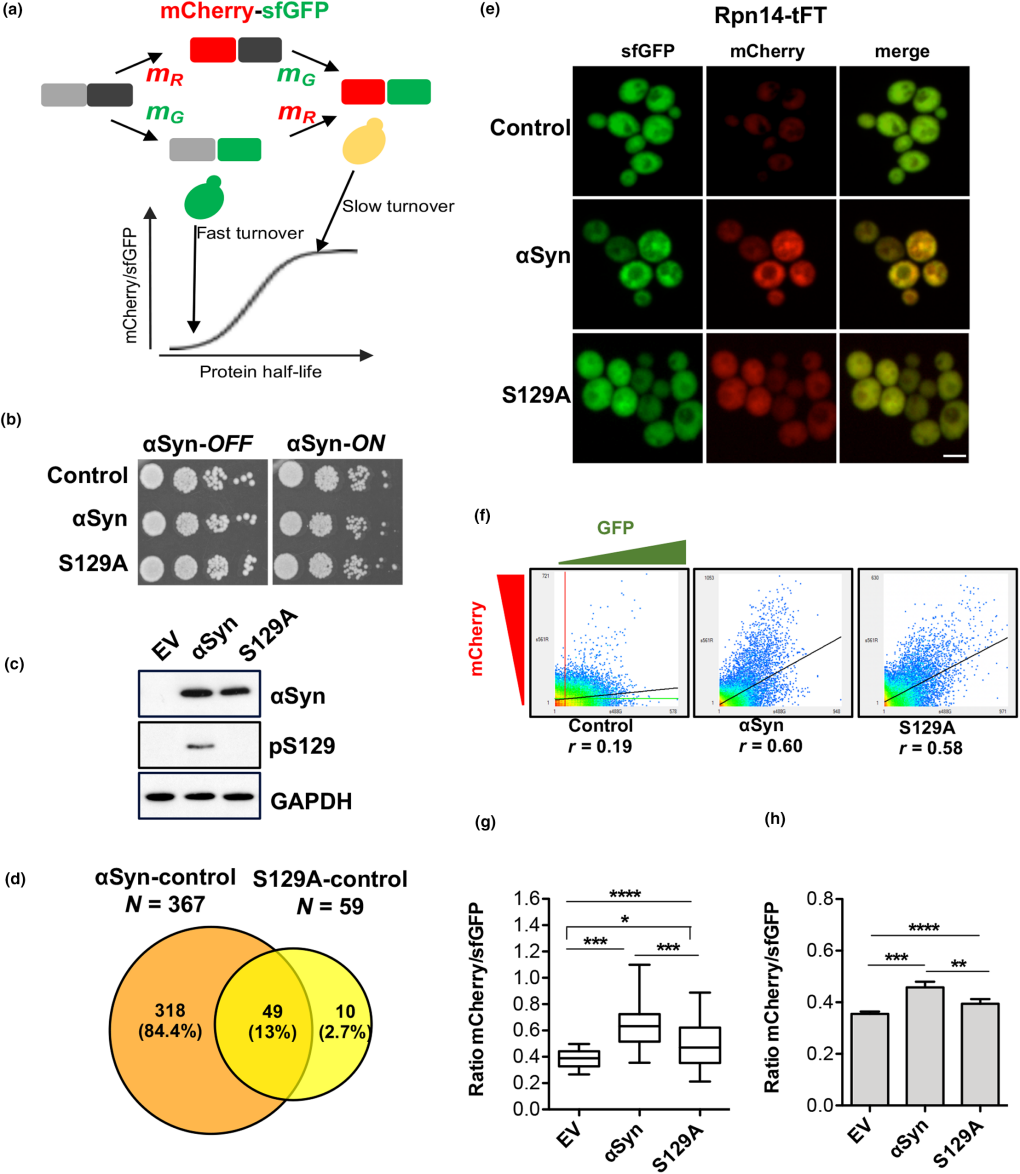
Expression of α‐syn has higher impact on yeast protein stabilities than the non‐phosphorylated S129A variant. (a) Schematic representation of tFT timer composed of a slow maturing mCherry (grey/red with maturation rate constant m_R_), and a fast maturing sfGFP (black/green with maturation rate constant m_G_). The mCherry/sfGFP ratio reports on the stability of the tFT‐fusion protein. Fast turnover – fusions are degraded before mCherry maturation; slow turnover – the relative fraction of mature mCherry increases. (b) Growth assays of yeast strains expressing *GAL1*‐driven α‐syn or S129A from two genomically integrated copies used as query strains in the tFT screen, with empty vector as control. Cells were spotted on selective plates containing glucose (α‐syn‐*OFF*) or galactose (α‐syn‐*ON*). (c) Immunodetection of proteins from (b) using α‐syn‐specific or pS129‐specific antibodies. GAPDH antibody was used as loading control. (d) Venn diagram depicting unique und shared proteome between pairwise comparisons of proteins with significantly changed stabilities upon expression of α‐syn or S129A compared to the control (empty vector). (e) Fluorescence microscopy of *RPN14*‐tFT strain, expressing α‐syn or S129A or empty vector as control. Scale bar: 5 μm. (f) Scatterplot of the intensity of the pixels in GFP channel versus mCherry channel, as determined by fluorescence microscopy of *RPN14*‐tFT cells from (e). The black line in the scatterplot depicts linear regression of the data in the 2D histogram. *r* = Pearson's Correlation Coefficient for the colocalization analysis. (g) Quantification of the fluorescent signal in cells from (e). The ratio mCherry/sfGFP was calculated per cell. Significance of differences was calculated with one‐way ANOVA with Newman–Keuls post‐hoc test (****p < 0.0001; ***p < 0.001; *p < 0.01; *n* = 100). (h) Flow cytometry measurements of log‐phase cultures of Rpn14‐tFT strain expressing α‐syn, S129A or empty vector control. The intensity of mCherry and sfGFP signal was measured for 10,000 single cells per experiment. Significance of differences was calculated with one‐way ANOVA with Newman–Keuls post hoc test (****p < 0.0001; ***p < 0.001; **p < 0.01, *n* = 6).

Tandem fluorescent protein timer (tFT) fusions consist of two fluorescent proteins with different fluorophore maturation times, usually a fast maturing green fluorescent protein (e.g., superfolder GFP, sfGFP), and a slow maturing red fluorescent protein (e.g., mCherry). In such a tFT the green fluorescence reports on the abundance of the tagged protein, whereas the mCherry/sfGFP ratio reports on the average age of the population of tagged protein. Under steady state conditions this ratio is proportional to the turnover of the pool of tagged proteins (Khmelinskii et al., 2012) (Figure 1a). Using the tFT tag, a genome‐wide yeast library of >4000 strains representing most of the genes tagged C‐terminally with the tFT reporter was created. We used this resource (Khmelinskii et al., 2014) and yeast high‐throughput strain construction (Tong & Boone, 2006) to investigate changes in protein homeostasis of yeast upon overexpression of wild‐type and mutant human α‐syn.

For strain construction and crossing with the tFT library, we created query strains conditionally expressing two copies of α‐syn‐encoding gene or S129A variant using galactose inducible promoters and chromosomally integrated vectors. Empty vector served as control. The expression level from two gene copies does not affect growth of yeast cells under standard growth conditions (Petroi et al., 2012), ensuring similar growth for strains with α‐syn and controls (Figure 1b). Immunoblotting with crude protein extracts from the two strains confirmed equal protein levels of α‐syn and S129A within the corresponding yeast strains (Figure 1c).

Each of the three query strains was crossed to a yeast library comprising 4044 strains, where each strain harbors a distinct tFT‐tagged open reading frame (Khmelinskii et al., 2014). Synthetic genetic array (SGA) methodology was used for generation of haploid double mutant strains (Tong & Boone, 2006). The resulting yeast strains express single tFT‐tagged yeast ORFs together with either α‐syn, S129A or empty vector and were grown as an ordered array of 1528 colonies per plate. To investigate tFT fluorescence in the strains the colonies were pinned on fresh plates containing 2% galactose to induce *GAL1*‐driven α‐syn or S129A expression. Following an incubation for 24 h we used a plate reader for high‐throughput plate fluorescence measurements of mCherry and sfGFP signal in each of the colonies. Subsequent analysis quantified the ratios of mCherry/sfGFP fluorescence intensities in presence of α‐syn or S129A and the changes of these ratios relative to cells without α‐syn (empty vector control).

A total of 377 proteins exhibited significant changes in mCherry/sfGFP ratios upon α‐syn or S129A expression compared to the control strain (Figure 1d, Tables S3, S4). Among them, 49 proteins displayed altered mCherry/sfGFP ratios in response to both α‐syn as well as S129A variant, 318 proteins upon α‐syn expression alone, whereas a total of 10 proteins had changed mCherry/sfGFP ratios in presence of S129A alone. Flow cytometry measurements with representatives of different functional categories confirmed the results obtained in the tFT‐screen (Figure S1). These data support that the effect of α‐syn with intact S129 phosphorylation site on the changes of individual mCherry/sfGFP ratios is considerably more profound in comparison to that of the S129A phosphorylation‐deficient variant.

### 3.2 Proteasomal Rpn14 chaperone is stabilized upon expression of α‐syn

The observation that mCherry/sfGFP ratios change for almost 10% of the yeast proteins tagged with the tFT reporter suggests that α‐syn expression could disturb key processes needed to protein homeostasis in yeast cells. Inspection of the list of proteins with changes in mCherry/sfGFP ratios identified enrichment of proteins involved in DNA replication and repair, mitosis, mRNA processing, nuclear transport, transcription, and mitochondria (Figure S2, Table S5). Many of these processes are involved in dynamic regulation of the cell and include proteins that are subject to regulation by selective proteolysis via the proteasome. One explanation could be that α‐syn overexpression leads to saturation of selective protein degradation. Alternatively, α‐syn affects the cellular machinery of selective protein degradation in a more specific manner. A closer inspection of the list of proteins with increased mCherry/sfGFP ratios identified Rpn14, a proteasome assembly chaperone to be among the particularly stabilized proteins by α‐syn expression. Rpn14 is involved in the assembly of the base subcomplex of the 19S proteasome regulatory particle (RP) and antagonizes the interaction of the base with the 20S core particle (CP) (Funakoshi et al., 2009; Park et al., 2009; Roelofs et al., 2009; Saeki et al., 2009). It interacts with the Rpt6 base subunit and enhances the assembly of the proteasome (Ehlinger et al., 2013).

Live cell fluorescence microscopy of yeast cells expressing *RPN14*‐tFT and *GAL1*‐driven α‐syn or S129A confirmed the results obtained in the genomic screening. The ratios of mCherry to sfGFP fluorescence signals showed a stronger effect on Rpn14 stabilization upon α‐syn expression compared to S129A expression (Figure 1e–g). Similar results were obtained with flow cytometry measurements of *RPN14*‐tFT strain in presence or absence of α‐syn or S129A (Figure 1h). The effect of α‐syn expression on Rpn14 stability was further investigated by cycloheximide‐chase experiments, utilizing Rpn14‐GFP instead of the relatively large tFT‐tag (Figure S3). α‐Synuclein expression led to enhanced stability of Rpn14‐GFP. These data support the notion that expression of α‐syn significantly increases Rpn14 stability and that this effect is promoted by S129 phosphorylation.

### 3.3 High protein levels of Rpn14 enhance α‐syn‐associated growth retardation

**FIGURE 2:**
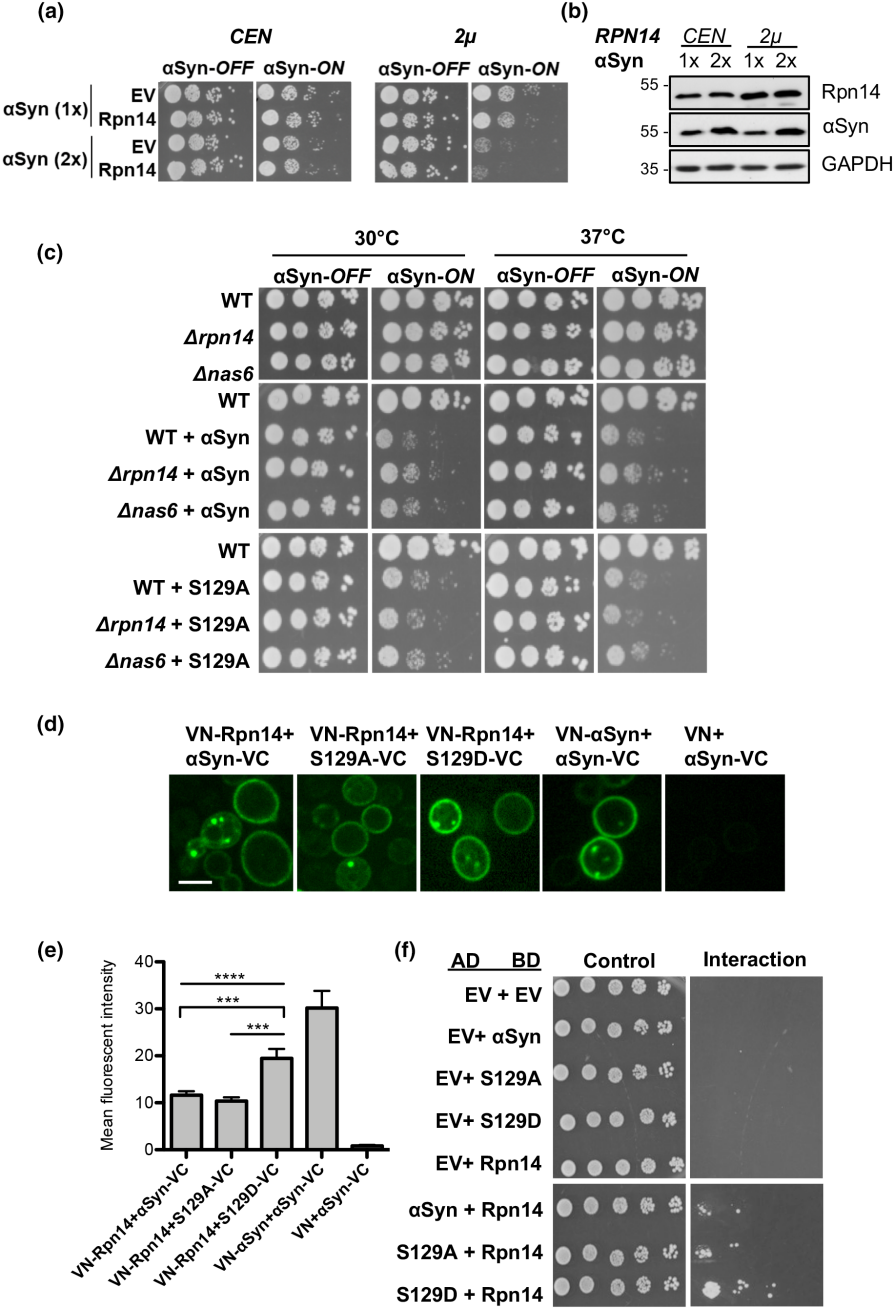
High Rpn14 protein levels enhance α‐syn‐associated cellular growth retardation. (a) Growth assays of yeast cells expressing *GAL1*‐driven α‐syn‐GFP from one (1×) or two (2×) gene copies, and *RPN14* from a low copy *CEN* plasmid or a high copy 2 μ plasmid with empty vector (EV) as control. (b) Immunodetection of proteins from (a) after 6 h induction of *GAL1* promoter. Rpn14 was expressed as a His‐tagged fusion and detected with anti‐His6 antibody. GAPDH antibody was used as loading control. (c) Growth assays of wild type (WT), *Δrpn14* or *Δnas6* yeast strain in presence or absence of α‐syn or S129A expressed from 2 μ plasmids. The plates were incubated in parallel at 30°C and 37°C for 4 days. (d) Bimolecular Fluorescence Complementation assay (BiFC). Rpn14, α‐syn, S129A, or S29D were fused to the N‐ or C‐terminal fragments of fluorescent Venus reporter protein (VN and VC). Live‐cell fluorescence microscopy of yeast cells, expressing different combinations of the fusion constructs 6 h post induction. BiFC of VN‐α‐syn+α‐syn‐VC served as positive control, and VN + α‐syn‐VC as negative control. Images are scaled to min/max pixel intensity for optimization of signal to noise ratio. Scale bar = 5 μm. (e) Quantification of BiFC signal intensities from (d). Significance of differences was calculated with one‐way ANOVA with Newman–Keuls post hoc test (****p < 0.0001; ***p < 0.001, *n* = 3). (f) Yeast‐two‐hybrid assay. Yeast cells were transformed with plasmids encoding the indicated proteins fused to the B42‐activation domain (AD) under the control of *GAL1* inducible promoter (prey), or LexA‐DNA‐binding domain (BD) driven by the constitutive *ADH* promoter (bait). Cells were spotted on SC‐His‐Trp + glucose selection plates as a control for equal dilution. The *LEU2* was used as a reporter gene for growth upon interaction of the bait and prey on selective medium lacking leucine (SC‐His‐Trp‐Leu + galactose).

The effects of different Rpn14 protein levels on yeast cells were evaluated to examine whether α‐syn‐induced toxicity is connected with the stabilization of the proteasomal chaperone. Yeast strains with defined copy numbers for α‐syn‐encoding genes were used for sensitive monitoring of small changes in cytotoxicity. Expression of α‐syn from one or two gene copies did not inhibit yeast growth (Petroi et al., 2012). Similarly, expression of *RPN14* from a low copy *CEN* plasmid in addition to the endogenous gene copy was not sufficient to affect growth of α‐syn expressing cells (Figure 2a). However, when *RPN14* was expressed from a high copy 2 μ plasmid, it enhanced α‐syn‐induced growth retardation upon expression of two copies of α‐syn (Figure 2a,b). Growth in liquid medium resulted in similar effects (Figure S4a). Further, it was assessed whether the impact of Rpn14 on α‐syn cytotoxicity depends on S129 phosphorylation. The human kinase GRK5 is known to phosphorylate α‐syn at S129 in human as well as in yeast cells (Shahpasandzadeh et al., 2014) and was co‐expressed to increase the fraction of pS129. Cytotoxicity of α‐syn was examined under stress conditions at elevated temperature with growth assays or by flow cytometry. Propidium iodide (PI) staining was employed as a sensitive method to determine cell viability from the fraction of cells with compromised membrane integrity. Increased levels of Rpn14 decreased cell growth (Figure S4b) or cell viability (Figure S4c–e). Importantly, this effect correlated with the level of pS129. These results corroborate that elevated levels of Rpn14 enhance α‐syn cytotoxicity and this effect correlates with α‐syn phosphorylation at S129.

The *Rpn14*‐dependent enhancement of α‐syn growth inhibition was not accompanied by changes in α‐syn inclusion formation within cells (Figure S5). Similarly, no effect on α‐syn inclusion formation was observed in *Δrpn14* strain in comparison to the isogenic wild‐type background. These results indicate that the Rpn14‐dependent enhancement of α‐syn cytotoxicity is independent of α‐syn aggregate formation.

Rpn14 functions redundantly with Nas6, another proteasomal chaperone that binds to the Rpt3 base subunit (Funakoshi et al., 2009; Saeki et al., 2009). Nas6 regulates the association of the lid and CP with the base and may protect against assembly of structurally defective 26S proteasomes (Li et al., 2017; Nemec et al., 2019). To elucidate whether the observed enhancement of α‐syn toxicity by elevated protein levels of Rpn14 is specific for this chaperone, we examined the effect of *RPN14* or *NAS6* deletion on yeast cell growth at 30 and 37°C (Figure 2c). No effect on α‐syn, or S129A‐induced toxicity was observed at 30°C in the corresponding deletion strains in comparison to wild type. However, deletion of *RPN14* resulted in partial rescue of α‐syn‐induced toxicity at 37°C. Similar growth rescue was not detected in the *Δnas6* strain, suggesting that the α‐syn effect is specific for Rpn14. Cells expressing S129A grew similarly in presence or absence of the proteasomal chaperones. Growth in liquid medium at 30°C and 37°C resulted in similar effects (Figure S6a,b). Additionally, S129D mutant was used that mimics constant phosphorylation at S129 residue. For quantitative assessment of cell viability, flow cytometry measurements of PI‐stained cells were performed (Figure S6c–f). Deletion of *RPN14* diminished the cells with compromised membrane integrity upon expression of α‐syn or S129D but not S129A, corroborating that native expression of Rpn14 increases the toxicity of pS129.

In summary, elevated levels of the proteasomal chaperone Rpn14 enhance α‐syn‐mediated growth retardation and viability. The increased stability of Rpn14 upon α‐syn expression presumably mediates toxicity resulting in cellular growth inhibition.

### 3.4 Rpn14 interacts with α‐syn

We assessed whether stabilization of Rpn14 upon α‐syn expression is due to their physical interaction. Bimolecular Fluorescence Complementation assays (BiFC) were performed to visualize protein–protein interactions (Popova, Wang, et al., 2021). Rpn14, α‐syn, S129A, or S129D were fused to the nonfluorescent complementary N‐ and C‐terminal fragments of the fluorescent reporter protein Venus (VN and VC). Co‐expression of α‐syn‐VC and VN‐Rpn14 constructs in yeast yielded green fluorescence indicating reconstitution of the Venus fluorophore by the interaction of Rpn14 with α‐syn (Figure 2d). The efficiency of the fluorescence complementation was quantified by the fluorescence intensity per cell (Figure 2e). Co‐expression of S129A‐VC and VN‐Rpn14 showed similar intensities of the BiFC signals. Importantly, co‐expression of Rpn14 with S129D revealed higher intensity of the reconstituted fluorophore which supports that this pair is more competent to physically interact.

The physical interactions between α‐syn and Rpn14 were verified using yeast‐two‐hybrid (Y2H) assays. The genes for the proteins of interest were fused to a sequence for a transcriptional activation domain (AD) (prey), or to DNA‐binding domain (BD) (bait) (Golemis et al., 1999). Upon interaction of the two proteins, transcription of the reporter gene *LEU2* is activated and can be validated by growth on selection medium lacking leucine. The Y2H experiment corroborated interaction between Rpn14 and α‐syn (Figure 2f). Yeast cells co‐expressing Rpn14 with α‐syn or S129A fusion constructs grew similarly on selection medium, whereas co‐expression of Rpn14 with S129D showed enhanced growth. These results together with the BiFC data corroborate that there is a physical interaction between α‐syn and Rpn14 and that pS129 further promotes this interaction.

Next, we addressed whether α‐syn interacts with functional 26S proteasomes. *RPN11‐3xFLAG* strain was used for purification of 26S proteasomes in presence of α‐syn and the pull‐down fractions were further enriched by gradient centrifugation (Figure S7). Immunoblotting analysis revealed that monomeric α‐syn as well as α‐syn oligomeric species co‐purify with the 26S proteasome complex, indicating their interaction with the native proteasome.

### 3.5 Elevated protein levels or depletion of Rpn14 is deleterious for yeast cells upon proteasome stress

**FIGURE 3:**
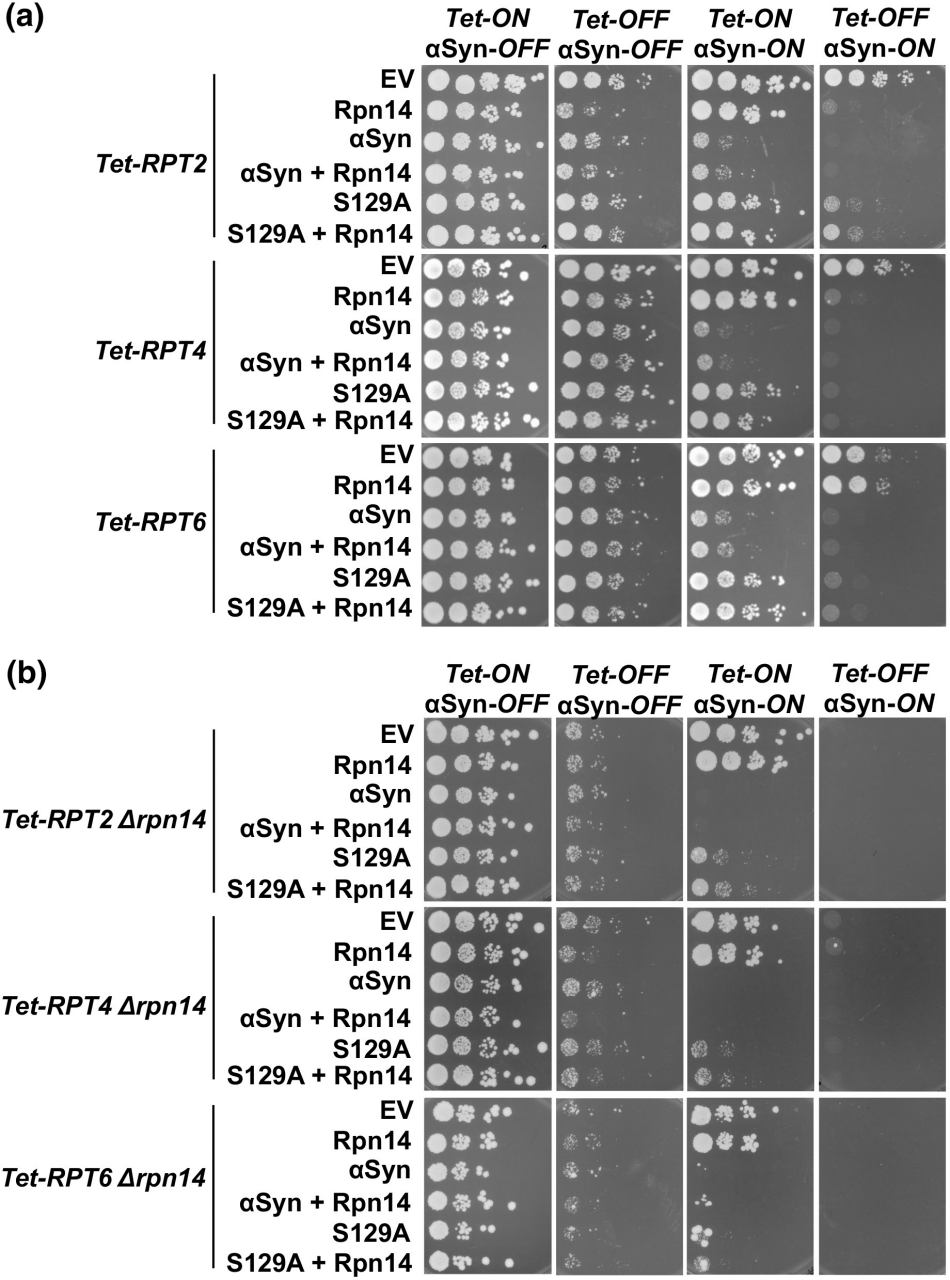
Elevated protein level or depletion of Rpn14 causes growth impairment upon proteasome stress. (a) Growth assays of yeast cells expressing *GPD*‐driven *RPN14* from *CEN* plasmid and *GAL1*‐driven α‐syn or S129A from 2 μ plasmid with an empty vector (EV) as a control in *Tet‐RPT2*, *Tet‐RPT4* and *Tet‐RPT6* yeast strains, or in *Tet‐RPT2 Δrpn14*, *Tet‐RPT4 Δrpn14*, and *Tet‐RPT6 Δrpn14* yeast strains (b). Cells were spotted on selective plates containing glucose (α‐syn‐*OFF*) or galactose (α‐syn‐*ON*). Repression of *Tet*‐promoter (*Tet*‐OFF) was achieved by addition of 2 μg/mL doxycycline in the plates.

Downregulation of the base proteasome subunit genes *RPT2*, *RPT4*, and *RPT6* significantly enhances α‐syn toxicity. Furthermore, elevated levels of α‐syn increase the pool of ubiquitinated substrates upon downregulation of *RPT2* (Popova, Galka, et al., 2021). Therefore, we next examined whether the impact of α‐syn is affected by different levels of Rpn14 in growth assays (Figure 3). We employed yeast strains from the *Tet*‐Promoters Hughes collection (yTHC), in which the endogenous promoter of each gene is replaced with a *Tet*‐titratable promoter (Mnaimneh et al., 2004). This substitution allows the deactivation of the *Tet‐RPT2*, *Tet‐RPT4*, or *Tet‐RPT6* genes by addition of doxycycline to the yeast growth medium. Double mutant strains were generated to elucidate further the role of Rpn14, where *RPN14* gene was deleted in the background of the three *Tet*‐strains. Elevated level of *RPN14* was achieved by expression from a *CEN* plasmid in addition to the genomic copy of the gene. Yeast cells, expressing α‐syn S129A or *RPN14* were spotted on glucose (α‐syn‐*OFF*) or galactose (α‐syn‐*ON*) containing plates. *Tet*‐promoter was repressed by addition of doxycycline. In all *Tet*‐strain with intact *RPN14* gene, the observed growth impairment upon expression of α‐syn was more severe than upon expression of S129A (Figure 3a). Downregulation of each of the three genes in presence of α‐syn or S129A resulted in severe synthetic‐sick phenotype. Strong growth impairment was observed due to elevated protein level of Rpn14 upon downregulation of *Tet‐RPT2* and *Tet‐RPT4*. High level of Rpn14 did not affect yeast growth upon downregulation of *Tet‐RPT6*. The synthetic‐sick phenotype of *Tet‐RPT6 Δrpn14* under *Tet‐OFF* α‐syn‐*ON* conditions could not be restored by ectopic expression of Rpn14, presumably due to the deletion, which has caused a genomic imbalance affecting cell survival (Teng et al., 2013). Growth assays in *Tet‐RPT2 Δrpn14*, *Tet‐RPT4 Δrpn14*, and *Tet‐RPT6 Δrpn14* revealed a strong synthetic‐sick phenotype upon downregulation of each of the three genes (Figure 3b), whereas growth on galactose‐containing plates resulted in synthetic‐lethal phenotype by downregulation of the *Tet*‐promoter.

These results demonstrate that yeast cells are highly sensitive to changed protein levels of Rpn14, indicating that normal protein levels of Rpn14 chaperone are important for the cellular well‐being upon proteasome stress conditions.

### 3.6 Increased Rpn14 or α‐syn level inhibit the degradation of ubiquitin conjugates upon proteolytic stress

The interplay of Rpn14 and α‐syn was further studied under proteolytic stress conditions. α‐Synuclein significantly alters ubiquitin homeostasis (Popova, Galka, et al., 2021). It was examined whether the impact of α‐syn is affected by different protein levels of Rpn14. Changes in the ubiquitin pool by downregulation of the essential genes *RPT2*, *RPT4*, and *RPT6* and in presence or absence of Rpn14, α‐syn, or S129A were examined.

**FIGURE 4:**
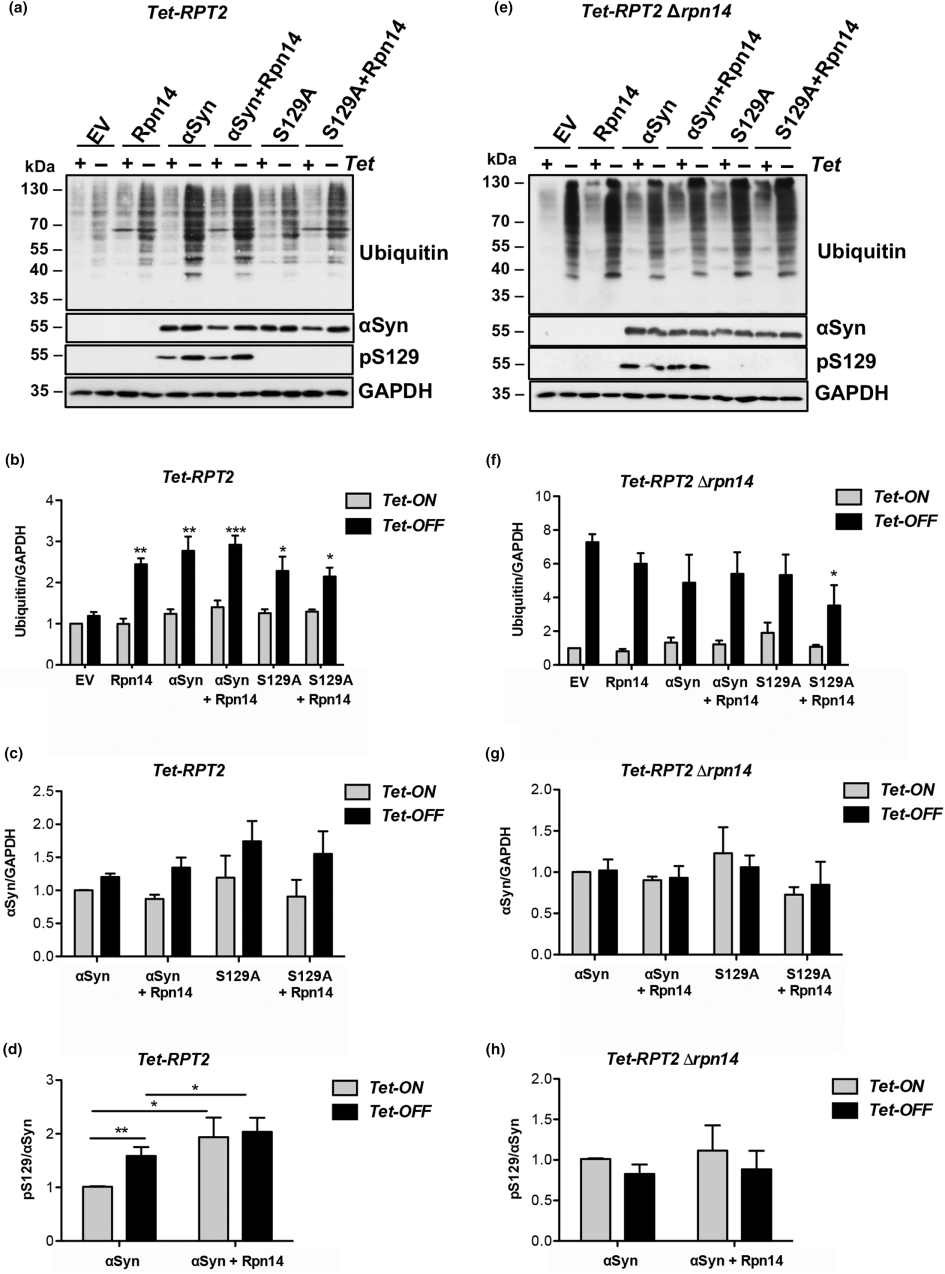
Rpn14 is directly involved in pS129 α‐syn turnover. (a) Immunoblot analysis of *Tet‐RPT2* strain expressing *GPD*‐driven *RPN14*, *GAL1*‐driven α‐syn‐GFP or S129A‐GFP. Empty vector (EV) was used as control. Yeast cells were grown overnight in galactose‐containing medium to induce α‐syn expression. The *Tet* promoter was repressed by simultaneous addition of 10 μg/mL doxycycline to the growth medium. (+) indicates *Tet‐ON*, and (−) *Tet‐OFF*. Immunoblotting analyses were performed with anti‐ubiquitin, α‐syn, or pS129 antibodies. GAPDH was used as a loading control. (b) Densitometric analysis of the immunodetection of the ubiquitin conjugates in *Tet‐RPT2* strain relative to GAPDH. Significance of differences was calculated with *t*‐test relative to EV control (*p < 0.05; **p < 0.01; ***p < 0.001). (c) Densitometric analysis of α‐syn protein levels relative to GAPDH. (d) Densitometric analysis of the fraction of pS129 relative to α‐syn. Significance of differences was calculated with *t*‐test (*p < 0.05; **p < 0.01). (e) Immunoblot analysis of *Tet‐RPT2 Δrpn14* strain expressing *GPD*‐driven *RPN14*, *GAL1*‐driven α‐syn‐GFP or S129A‐GFP, performed under the same conditions as in (a). (f) Densitometric analysis of the immunodetection of the ubiquitin conjugates in *Tet‐RPT2 Δrpn14* strain relative to GAPDH. The significance of differences was calculated with *t*‐test relative to EV control. (g) Densitometric analysis of α‐syn protein levels from *Tet*‐*RPT2 Δrpn14* (e) relative to GAPDH. (h) Densitometric analysis of pS129 fraction relative to α‐syn signal in *Tet*‐*RPT2 Δrpn14* strain.

The levels of ubiquitin conjugates in *Tet‐RPT2* strain were analyzed by immunoblotting (Figure 4a,b). Rpt2 is one of the AAA+ ATPase subunits of the 19S regulatory particle (RP), which is essential for substrate unfolding and translocation (Sakata et al., 2021). An α‐syn subpopulation is localized in proximity of Rpt2 (Popova, Galka, et al., 2021). α‐Synuclein may directly or indirectly interact with the 19S RP, which could interfere with the assembly with the 20S core particle to 26S proteasome. Downregulation of the *Tet‐RPT2* had no effect on the level of ubiquitinated conjugates when compared to *Tet‐ON* in the empty vector control (Figure 4a,b). Similarly, expression of α‐syn or elevated level of Rpn14 did not alter the accumulation of ubiquitinated proteins upon *Tet‐ON*. In contrast, downregulation of *Tet‐RPT2* upon expression of α‐syn, elevated level of Rpn14, or both resulted in a significant increase of the ubiquitin conjugates compared to the empty vector control. A synergistic effect of Rpn14 and α‐syn expression was not observed. Significantly less accumulation of ubiquitin conjugates was observed when S129A was expressed in comparison to α‐syn. Expression of S129D increased the accumulation of ubiquitin conjugates compared to α‐syn (Figure S8). This supports our findings that α‐syn phosphorylated at S129 affect cellular proteostasis significantly more than the non‐phosphorylatable S129A version. In summary, the results corroborate that elevated levels of the Rpn14 chaperone contribute to proteasome dysfunction under proteotoxic stress conditions.

### 3.7 Rpn14 is involved in the turnover of phosphorylated α‐syn

The consequences of increased levels of Rpn14 on α‐syn or S129A turnover were compared. Steady‐state protein levels of α‐syn and S129A were not significantly changed (Figure 4c). However, significant accumulation of phosphorylated α‐syn was observed upon elevated protein level of Rpn14 (Figure 4d), independent of the expression level of *Tet‐RPT2*. Thus, Rpn14 protein is linked to the accumulation of the pS129 fraction, indicating that Rpn14 suppresses pS129 α‐syn turnover.

The accumulation of ubiquitinated proteins was analyzed in a *Tet‐RPT2 Δrpn14* strain (Figure 4e). The levels of ubiquitinated proteins were similar to that of the control cells, when α‐syn or *RPN14* were expressed, in contrast to the same conditions in a *Tet‐RPT2* strain. These results suggest that α‐syn‐induced accumulation of ubiquitin conjugates when Rpt2 is depleted depends on the presence of Rpn14. The protein abundance of α‐syn in *Tet‐RPT2 Δrpn14* strain was similar in all examined conditions independently from the *RPT2* expression level (Figure 4g). Immunoblotting with a pS129 antibody revealed that the fraction of phosphorylated α‐syn protein did not change in *Tet‐RPT2 Δrpn14* (Figure 4h), in contrast to a *Tet‐RPT2* strain with intact *RPT14*. These results reveal that elevated levels of Rpn14 inhibit the turnover of phosphorylated α‐syn.

Similar analyses of the changes in ubiquitin pool and α‐syn turnover were performed in *Tet‐RPT4* (Figure S9), *Tet‐RPT6* (Figure S10) and in the corresponding *RPN14* deletion strains. Rpn14 interacts with the base ATPase Rpt6 leading to its translocation to the α‐subunits of the core particle (Ehlinger et al., 2013). Similar to *Tet‐RPT2*, an increase in the accumulation of ubiquitinated proteins was observed under downregulation of *Tet‐RPT4* when α‐syn was expressed or the level of Rpn14 was elevated. The differences between *Tet‐ON* and *Tet‐OFF* were less prominent in comparison to *Tet‐RPT2*. In contrast to *Tet‐RPT2* and *Tet‐RPT4*, accumulation of ubiquitinated proteins occurred in the empty vector control, when the proteasome base subunit gene *RPT6* was downregulated (Figure S10a,b). Downregulation of *Tet‐RPT6* resulted in a significant increase of phosphorylated fraction of α‐syn; however the level was not dependent on Rpn14 (Figure S10d), in contrast to *Tet‐RPT2* background (Figure 4d). These results indicate distinct cellular responses to the expression of *RPN14* and α‐syn upon depletion of one of the base subunits Rpt2, Rpt4, or Rpt6. Therefore, α‐syn disturbs the proteasome function via multiple pathways.

### 3.8 α‐Synuclein‐induced inhibition of 26S activity is mediated by Rpn14

**FIGURE 5:**
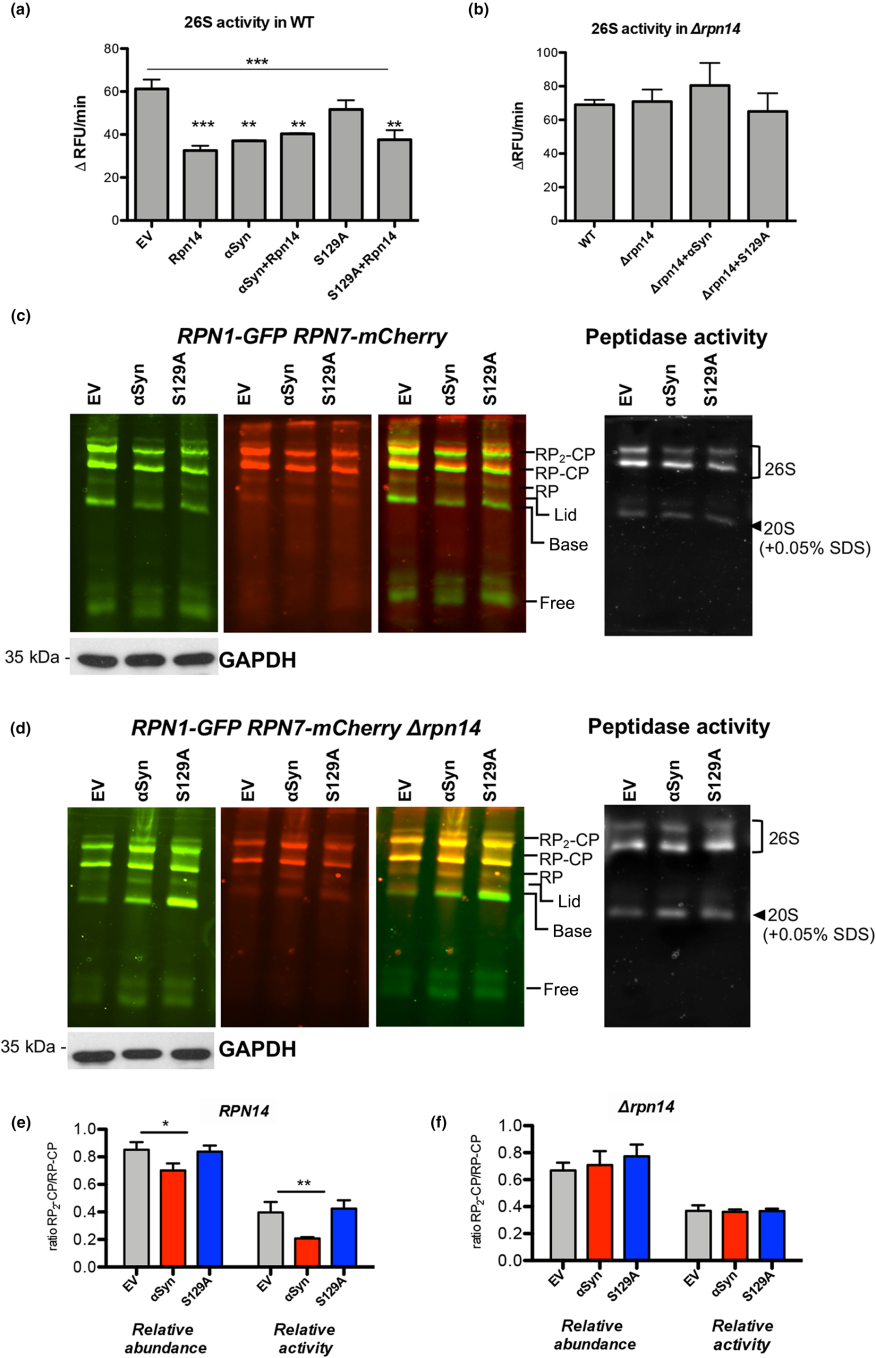
Rpn14 is required for α‐syn‐induced decrease of 26S proteasomal activity. (a) Wild‐type (WT) yeast cells expressing *RPN14* from *CEN* plasmid, α‐syn or S129A from 2 μ plasmid, or empty vector (EV) as control were collected after 16 h of *GAL1* induction. The 26S chymotrypsin‐like proteasomal activity in crude protein extracts was monitored by measuring the hydrolysis of the fluorogenic peptide Suc‐LLVY‐AMC by detecting relative fluorescence units (RFU) per minute. Significance of differences was calculated with one‐way ANOVA with Newman–Keuls post hoc test (***p < 0.001, **p < 0.01; *n* = 4). (b) 26S chymotrypsin‐like proteasomal activity in *Δrpn14* strain, performed similarly as in (a). (c) Extracts from cells expressing *RPN1‐GFP*, *RPN7‐mCherry*, α‐syn, S129A or EV control were prepared in presence of ATP and resolved on 4% native PAGE. Fluorescence of Rpn1‐GFP and Rpn7‐mCherry was imaged by a fluoroimager. The protein bands assigned to the two isoforms of the 26S proteasome (RP_2_‐CP and RP‐CP), free RP, base, and lid are indicated. The gel was overlaid with Suc‐LLVY‐AMC to monitor the 26S proteasome activities and afterwards in the presence of 0.05% SDS to visualize CP activity. (d) Native PAGE performed as in (c) in *Δrpn14* strain. (e, f) Relative fluorescence abundance and intensity determined from GFP fluorescence or in‐gel activity assays per lane from (c) and (d). Significance of differences was calculated with t test (**p < 0.01; *n* = 3).

The effects of α‐syn, S129A, or elevated Rpn14 levels on 26S proteasome activities were examined in wild‐type yeast (Figure 5a) in comparison to *Δrpn14* strain (Figure 5b). The chymotrypsin‐like proteasome activity was measured using the fluorogenic peptide Succinyl‐Leu‐Leu‐Val‐Tyr‐7‐amido‐4‐methylcoumarin (Suc‐LLVY‐AMC). The degradation of the fluorogenic peptide was measured by continuously monitoring the fluorescence of the reaction (Figure S11a,b). Crude protein extracts, treated with the proteasome inhibitor MG132 as control revealed that the kinetic assay is specific for proteasome cleavage (Figure S11c,d). Significant decrease of the 26S proteasome activity was caused by expression of α‐syn as well as upon *RPN14* expression in wild‐type yeast strain. This is in line with observations showing that overexpression of *RPN14* results in decreased 26S proteasome activity presumably because of inefficient assembly (Shirozu et al., 2015). Both proteins decreased 26S proteasome activities to similar levels without further synergistic effects. In contrast, 26S proteasome activity upon expression of S129A was not significantly changed. Co‐expression of *RPN14* and S129A reduced 26S activities, indicating that 26S proteasome inhibition is primarily caused by elevated Rpn14 protein levels. The 26S proteasome activity was compared in a *Δrpn14* strain, in presence or absence of α‐syn or S129A. Deletion of *RPN14* revealed similar proteasome activity as wild type, in accordance with previous reports (Saeki et al., 2009; Seong et al., 2007). Importantly, inhibition of 26S proteasome activity was not observed upon expression of α‐syn.

The mechanism by which α‐syn affects 26S proteasome activity in presence or absence of Rpn14 was further investigated. Fluorescent protein imaging was used to monitor proteasome assembly and detect proteasome complexes with high resolution. The base, lid, RP, as well as their assemblies were monitored in strains, where the base subunit Rpn1 is tagged with GFP, and the lid subunit Rpn7 with mCherry (Saeki et al., 2009). Protein extracts prepared in the presence of ATP were resolved on 4% native PAGE. Multiple bands were detected that were assigned to RP_2_‐CP, RP‐CP, RP, base, and lid (Figure 5c). Similar native PAGE was performed with *RPN1‐GFP RPN7‐mCherry Δrpn14* strain (Figure 5d). The activities of double‐capped 30S (RP_2_‐CP) and single‐capped (RP‐CP) 26S proteasomes were visualized by in‐gel activity assays using Suc‐LLVY‐AMC, followed by visualization of the latent CP peptidase activity in the presence of 0.05% SDS. The abundance of single‐capped and double‐capped proteasomes, measured from the GFP fluorescence signals, and their corresponding activities were evaluated relative to each other per lane to exclude small differences in loading (Figure 5e). In presence of α‐syn and Rpn14, the relative abundance and activity of RP_2_‐CP was significantly reduced in comparison to the control. In contrast, the relative abundance and activity of RP_2_‐CP proteasomes in presence of α‐syn did not differ from the control in *Δrpn14* cells (Figure 5f). S129A expression did not change the ratio of double‐capped to single capped 26S proteasome activities. These findings reveal that α‐syn affects the activity and assembly of 26S proteasomes when *RPN14* is present and further demonstrate that Rpn14 functions presumably through physical interaction as mediator for α‐syn‐induced proteasome inhibition.

### 3.9 Human PAAF1, a counterpart of yeast Rpn14, enhances α‐syn‐induced proteasome inhibition

**FIGURE 6:**
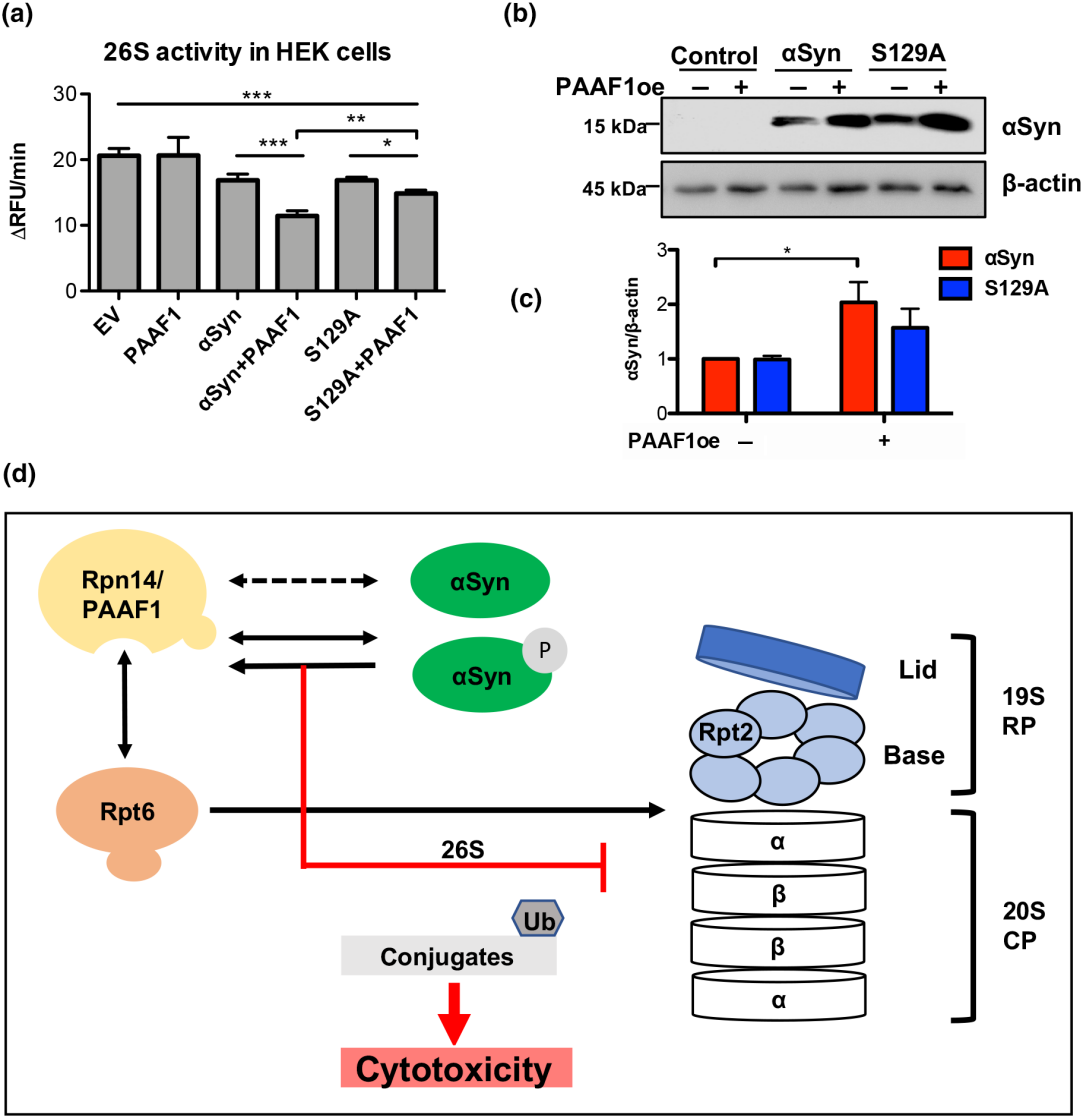
Human PAAF1 corresponding to yeast Rpn14 enhances α‐syn‐induced inhibition of 26S proteasomal activity. (a) HEK cells were transfected with constructs expressing *PAAF1*, α‐syn, or S129A under the control of the *CMV* promoter. EV, empty vector. The 26S chymotrypsin‐like proteasomal activity was assayed in crude protein extracts by measuring the hydrolysis of Suc‐LLVY‐AMC per minute. Significance of differences was calculated with one‐way ANOVA with Newman–Keuls post hoc test (***p < 0.001; **p < 0.01; *p < 0.05; *n* = 4). (b) Immunoblot analysis of probes from (a) using α‐syn‐antibody. β‐Actin was used as a loading control. (c) Densitometric analysis of α‐syn protein levels from (b) relative to β‐actin. Significance of differences was calculated with t test (*p < 0.05; *n* = 4). (d) α‐syn inhibition of 26S proteasome activity is mediated by the proteasomal assembly chaperone Rpn14/PAAF1. Rpn14 binds Rpt6 base subunit and escorts it to the core particle. α‐syn physically interacts with and stabilizes Rpn14, which leads to reduction of double‐capped proteasomes and decreased 26S proteasomal activity. Phosphorylated α‐syn and increased levels of Rpn14 cause accumulation of ubiquitin conjugates under proteasome stress.

Key features of proteasome base assembly are conserved between yeast and mammals. The chaperone function of yeast Rpn14 is paralleled in mammals by the proteasome‐interacting protein, proteasomal ATPase‐associated factor 1 (PAAF1). PAAF1 binds to PSMC5 base subunit that corresponds to yeast Rpt6, and is required for targeted degradation of unassembled intermediates to maintain protein homeostasis (Park et al., 2005; Zavodszky et al., 2021). The interplay of PAAF1 and α‐syn on 26S proteasome activity was examined in mammalian cells. Human embryonic kidney 293 (HEK) cells were co‐transfected with *PAAF1*, α‐syn, or S129A expressing constructs. The 26S proteasome activities were measured in cell lysates using Suc‐LLVY‐AMC peptide substrate (Figure 6a). *PAAF1* expression alone did not affect proteasome activity. However, expression of *PAAF1* together with α‐syn significantly enhanced α‐syn‐induced proteasome inhibition. This effect was less pronounced upon co‐expression of *PAAF1* with S129A. Immunoblot analysis of α‐syn steady‐state levels revealed almost twofold accumulation of α‐syn protein upon elevated PAAF1 protein level (Figure 6b,c). This reflects a direct correlation between α‐syn accumulation and decreased 26S proteasome activity probably as a result of positive feedback loop. These data corroborate the results for Rpn14 in yeast and suggest that PAAF1 is a key mediator of α‐syn‐induced proteasome inhibition in human cells.

## 4 DISCUSSION

Aggregated α‐syn is a key component of Lewy bodies in the brain of PD patients and plays a crucial role in the disease progression. Accumulation of oligomeric and aggregated species reveals dysfunctional cellular proteostasis, however the exact mechanisms leading to this imbalance are still elusive (Lehtonen et al., 2019). The main finding of this study is that α‐syn disturbs protein homeostasis by interacting with the proteasomal yeast chaperone Rpn14, which results in decreased activity of the 26S proteasome and reduced abundance of double‐capped 30S proteasome complexes. We demonstrated that α‐syn monomers and oligomers interact with the native 26S proteasome. This affects the stability of multiple proteins, as well as α‐syn‐induced cellular toxicity. α‐Synuclein phosphorylated at S129 (pS129) affects cellular proteostasis significantly more than the non‐phosphorylatable S129A version. α‐Synuclein increases the stability of the proteasomal yeast chaperone Rpn14, which corresponds to mammalian ortholog PAAF1. Rpn14/PAAF1 enhances the cytotoxic effect α‐syn on cells by decreasing the activity of 26S proteasome and inhibiting the degradation of α‐syn (Figure 6d).

The global effects of α‐syn expression on protein stabilities have not been thoroughly investigated yet. We employed a systematic screening approach with tandem fluorescent timer (tFT) fusions in combination with the power of yeast genetics to monitor the cytosolic turnover of proteins in presence of α‐syn and to identify factors with changed stability. The stability of almost 10 percent (377) of the 4044 yeast proteins examined was altered by the presence of α‐syn. Phosphorylated α‐syn (pS129) was found to affect protein stability significantly more than its non‐phosphorylated counterpart. This corroborates quantitative cellular proteomics studies, revealing significant alterations in protein homeostasis and decreased abundance of 10 proteasome subunits upon expression of α‐syn, dependent on S129 phosphorylation (Popova, Galka, et al., 2021). Phosphorylation of α‐syn at S129 is the main posttranslational modification of α‐syn and a major determinant for protein degradation (Stefanis et al., 2019). Intracellular homeostasis of α‐syn is maintained by endogenous regulatory mechanisms, including the ubiquitin‐proteasome system (UPS) and the autophagy (Petroi et al., 2012; Vilchez et al., 2014). Accumulation of α‐syn is strongly linked to the impairment of these degradation pathways (Xilouri et al., 2013). UPS dysfunction is an important aspect of α‐syn‐mediated toxicity. Monomeric as well as aggregated α‐syn binds to the 19S regulatory particle of the proteasome in vitro (Snyder et al., 2003). α‐Synuclein filaments can bind to the proteasome 20S core and are able to inhibit the chymotrypsin‐like hydrolytic activity (Lindersson et al., 2004). Phosphorylated α‐syn is found in the proximity to the Rpt2 base subunit and interferes with the proteasome function, resulting in an altered pool of ubiquitin conjugates (Popova, Galka, et al., 2021). Thus, impairment of proteasome activities which are connected to neurodegeneration may be mediated by physical contact of α‐syn to the regulatory or core proteasome particle. This results in alterations in proteasome composition, 26S assembly, or stability.

We identified the proteasomal chaperone Rpn14 as top candidate among the proteins that were particularly stabilized by the expression of pS129 α‐syn. The multiple α‐syn interactions with the 26S proteasome presumably include indirect as well as direct interactions. Yeast‐two‐hybrid data support a direct binary interaction between Rpn14 and α‐syn. Increased levels of Rpn14 enhanced α‐syn‐associated growth inhibition and the accumulation of ubiquitin conjugates in stressed conditions. Co‐expression of Rpn14 and α‐syn did not lead to additive accumulation of ubiquitinated species, suggesting the presence of additional cellular responses that aggravate cytotoxicity. Increased stress upon co‐expression of the two proteins might not only inactivate the proteasome, but also diminish ubiquitin conjugate levels due to inactivation of ubiquitin‐conjugating enzymes. This scenario could accelerate the accumulation of oxidatively damaged proteins, further compromising cell viability.

Rpn14 stabilizes pS129 α‐syn. The 26S proteasome activity decreased in presence of Rpn14 but not in the deletion mutant, indicating that the negative effect of pS129 on the 26S proteasome is mediated through the Rpn14 chaperone. Rpn14 is one of the four proteasome interacting proteins that govern the assembly of the yeast/mammalian 19S regulatory particle base, namely Rpn14/PAAF1, Nas2/p27, Nas6/gankyrin, and Hsm3/S5b. These proteins interact with their cognate Rpt subunits of the RP and form a distinct subassembly of base components, which escorts them to mature RPs (Funakoshi et al., 2009; Park et al., 2009; Roelofs et al., 2009; Saeki et al., 2009). Proteasomal chaperones provide “checkpoints” for the assembly of the RP and prevent the addition of other subunits until a proper assembly step is accomplished (Nahar et al., 2022). Normally, chaperones exist in sub‐stoichiometric levels to the total cellular RPs, and their specificity is maintained by their limited cellular level. Excess of the proteasomal chaperones may saturate the RPs by binding to every single particle. Overexpression of *RPN14* results in a decrease of 26S proteasome activity and increase of 20S activity (Shirozu et al., 2015), indicating that excess of Rpn14 can misregulate the assembly of the 26S proteasome and is detrimental to cellular integrity. Similarly, overexpression of PAAF1, the mammalian ortholog of Rpn14, inhibits the assembly of the 26S proteasome (Park et al., 2005). RP assembly checkpoint requires accurate chaperone levels for the nucleotide‐dependent switch, confirming that excess chaperones misregulate the complex proteasome holoenzyme (Nahar et al., 2022). Increased stability of Rpn14 due to α‐syn expression might change the stability of the Rpn14/Rpt6 subcomplexes and decrease the 26S proteasome assembly and activity. The base chaperones and CP compete for Rpt binding during assembly (Ehlinger et al., 2013) and increased Rpn14 stability may interfere with the effective interconversion and expulsion of Rpn14 during Rpt:CP assembly. A major finding of this study is that α‐syn interacts with Rpn14. A yet open question is, whether this interaction takes place on the proteasome or in the context of another Rpn14‐containing complex or is a combination of both. The complex dynamics and diversity of the proteasome pose challenges in determining the precise sequence of events leading to cellular dysfunction when Rpn14 and α‐syn are co‐expressed.

Phosphorylated and non‐phosphorylated α‐syn have different effects on UPS activity, possibly due to the structural and kinetic properties of the protein aggregates, dependent on PTMs. In a recent study, differently generated α‐syn fibrils were injected into mouse brains and it was found that fibrils with exposed C‐terminal regions reduced 26S activity and induced accumulation of phosphorylated S129 and ubiquitinated proteins, while fibrils with packed C‐terminus did not interact with the 26S proteasome (Suzuki et al., 2020). This reveals that the C‐terminal region of α‐syn interacts with the 26S proteasome, resulting in impaired activity which may be due to changed conformation of α‐syn C‐terminal region caused by phosphorylation at S129 that increases its negative charge.

The identified proteasomal chaperone Rpn14/PAAF1 represents a novel factor in α‐syn‐induced proteasome inhibition and contributes to a better understanding of the interplay between pS129 α‐syn and the 26S proteasome as the cellular degradation apparatus and its consequences, which contribute to PD.

## AUTHOR CONTRIBUTIONS

Conceptualization: BP, GHB, TO, MK, and ES; funding acquisition: GHB; investigation: DG, TA, AB, MN, EG, RM, and BP; supervision: BP and GHB; writing—original draft: DG, BP, and GHB; writing—review and editing: DG, TA, EG, ET, TO, MK, BP, and GHB.

## FUNDING INFORMATION

No funding information provided.

## CONFLICT OF INTEREST STATEMENT

The authors have no conflicts of interest to declare.

## Supporting information


Data S1 – Supporting methods.

Figures S1–S11.

Tables S1–S5.

## Data Availability

The data supporting the findings of this study are available in the main text and in the supporting information.

## References

[acel14128-bib-0001] Bentea, E. , Verbruggen, L. , & Massie, A. (2017). The proteasome inhibition model of Parkinson's disease. Journal of Parkinson's Disease, 7, 31–63. 10.3233/JPD-160921 PMC5302045 27802243

[acel14128-bib-0002] Ehlinger, A. , Park, S. , Fahmy, A. , Lary, J. W. , Cole, J. L. , Finley, D. , & Walters, K. J. (2013). Conformational dynamics of the Rpt6 ATPase in proteasome assembly and Rpn14 binding. Structure, 21, 753–765. 10.1016/J.STR.2013.02.021 23562395 PMC3670613

[acel14128-bib-0003] Eisele, M. R. , Reed, R. G. , Rudack, T. , Schweitzer, A. , Beck, F. , Nagy, I. , Pfeifer, G. , Plitzko, J. M. , Baumeister, W. , Tomko, R. J., Jr. , & Sakata, E. (2018). Expanded coverage of the 26S proteasome conformational landscape reveals mechanisms of peptidase gating. Cell Reports, 24, 1301–1315.e5. 10.1016/j.celrep.2018.07.004 30067984 PMC6140342

[acel14128-bib-0004] Emmanouilidou, E. , Stefanis, L. , & Vekrellis, K. (2010). Cell‐produced α‐synuclein oligomers are targeted to, and impair, the 26S proteasome. Neurobiology of Aging, 31, 953–968. 10.1016/j.neurobiolaging.2008.07.008 18715677

[acel14128-bib-0005] Funakoshi, M. , Tomko, R. J. , Kobayashi, H. , & Hochstrasser, M. (2009). Multiple assembly chaperones govern biogenesis of the proteasome regulatory Particle Base. Cell, 137, 887–899. 10.1016/J.CELL.2009.04.061 19446322 PMC2718848

[acel14128-bib-0006] Gietz, R. D. , & Woods, R. A. (2002). Transformation of yeast by lithium acetate/single‐stranded carrier DNA/polyethylene glycol method. Methods in Enzymology, 350, 87–96. 10.1016/S0076-6879(02)50957-5 12073338

[acel14128-bib-0007] Golemis, E. A. , Serebriiskii, I. , & Law, S. F. (1999). The yeast two‐hybrid system: Criteria for detecting physiologically significant protein‐protein interactions. Current Issues in Molecular Biology, 1, 31–45. 10.21775/cimb.001.031 11475699

[acel14128-bib-0008] Khmelinskii, A. , Blaszczak, E. , Pantazopoulou, M. , Fischer, B. , Omnus, D. J. , Le Dez, G. , Brossard, A. , Gunnarsson, A. , Barry, J. D. , Meurer, M. , Kirrmaier, D. , Boone, C. , Huber, W. , Rabut, G. , Ljungdahl, P. O. , & Knop, M. (2014). Protein quality control at the inner nuclear membrane. Nature, 516, 410–413. 10.1038/nature14096 25519137 PMC4493439

[acel14128-bib-0009] Khmelinskii, A. , Keller, P. J. , Bartosik, A. , Meurer, M. , Barry, J. D. , Mardin, B. R. , Kaufmann, A. , Trautmann, S. , Wachsmuth, M. , Pereira, G. , Huber, W. , Schiebel, E. , & Knop, M. (2012). Tandem fluorescent protein timers for in vivo analysis of protein dynamics. Nature Biotechnology, 30, 708–714. 10.1038/nbt.2281 22729030

[acel14128-bib-0010] Knop, M. , Siegers, K. , Pereira, G. , Zachariae, W. , Winsor, B. , Nasmyth, K. , & Schiebel, E. (1999). Epitope tagging of yeast genes using a PCR‐based strategy: More tags and improved practical routines. Yeast, 15, 963–972. 10.1002/(SICI)1097-0061(199907)15:10B<963::AID-YEA399>3.0.CO;2-W 10407276

[acel14128-bib-0011] Lehtonen, Š. , Sonninen, T.‐M. , Wojciechowski, S. , Goldsteins, G. , & Koistinaho, J. (2019). Dysfunction of cellular Proteostasis in Parkinson's disease. Frontiers in Neuroscience, 13, 457. 10.3389/fnins.2019.00457 31133790 PMC6524622

[acel14128-bib-0012] Li, F. , Tian, G. , Langager, D. , Sokolova, V. , Finley, D. , & Park, S. (2017). Nucleotide‐dependent switch in proteasome assembly mediated by the Nas6 chaperone. Proceedings of the National Academy of Sciences of the United States of America, 114, 1548–1553. 10.1073/pnas.1612922114 28137839 PMC5321026

[acel14128-bib-0013] Lindersson, E. , Beedholm, R. , Højrup, P. , Moos, T. , Gai, W. P. , Hendil, K. B. , & Jensen, P. H. (2004). Proteasomal inhibition by α‐synuclein filaments and oligomers. The Journal of Biological Chemistry, 279, 12924–12934. 10.1074/JBC.M306390200 14711827

[acel14128-bib-0014] McNaught, K. S. P. , & Jenner, P. (2001). Proteasomal function is impaired in substantia nigra in Parkinson's disease. Neuroscience Letters, 297, 191–194. 10.1016/S0304-3940(00)01701-8 11137760

[acel14128-bib-0015] McNaught, K. S. P. , Mytilin, C. , JnoBaptiste, R. , Yabut, J. , Shashidharan, P. , Jenner, P. , & Olanow, C. W. (2002). Impairment of the ubiquitin‐proteasome system causes dopaminergic cell death and inclusion body formation in ventral mesencephalic cultures. Journal of Neurochemistry, 81, 301–306. 10.1046/j.1471-4159.2002.00821.x 12064477

[acel14128-bib-0016] Mnaimneh, S. , Davierwala, A. P. , Haynes, J. , Moffat, J. , Peng, W.‐T. , Zhang, W. , Yang, X. , Pootoolal, J. , Chua, G. , Lopez, A. , Trochesset, M. , Morse, D. , Krogan, N. J. , Hiley, S. L. , Li, Z. , Morris, Q. , Grigull, J. , Mitsakakis, N. , Roberts, C. J. , … Hughes, T. R. (2004). Exploration of essential gene functions via titratable promoter alleles. Cell, 118, 31–44. 10.1016/j.cell.2004.06.013 15242642

[acel14128-bib-0017] Nahar, A. , Sokolova, V. , Sekaran, S. , Orth, J. D. , & Park, S. (2022). Assembly checkpoint of the proteasome regulatory particle is activated by coordinated actions of proteasomal ATPase chaperones. Cell Reports, 39, 110918. 10.1016/j.celrep.2022.110918 35675778 PMC9214829

[acel14128-bib-0018] Nemec, A. A. , Peterson, A. K. , Warnock, J. L. , Reed, R. G. , & Tomko, R. J. (2019). An allosteric interaction network promotes conformation state‐dependent eviction of the Nas6 assembly chaperone from nascent 26S proteasomes. Cell Reports, 26, 483–495.e5. 10.1016/J.CELREP.2018.12.042 30625330 PMC6344052

[acel14128-bib-0019] Oueslati, A. (2016). Implication of alpha‐synuclein phosphorylation at S129 in Synucleinopathies: What have we learned in the last decade? Journal of Parkinson's Disease, 6, 39–51. 10.3233/JPD-160779 PMC4927808 27003784

[acel14128-bib-0020] Outeiro, T. F. , & Lindquist, S. (2003). Yeast cells provide insight into alpha‐synuclein biology and pathobiology. Science, 302, 1772–1775. 10.1126/science.1090439 14657500 PMC1780172

[acel14128-bib-0021] Park, S. , Roelofs, J. , Kim, W. , Robert, J. , Schmidt, M. , Gygi, S. P. , & Finley, D. (2009). Hexameric assembly of the proteasomal ATPases is templated through their C termini. Nature, 459, 866–870. 10.1038/nature08065 19412160 PMC2722381

[acel14128-bib-0022] Park, Y. , Hwang, Y.‐P. , Lee, J.‐S. , Seo, S.‐H. , Yoon, S. K. , & Yoon, J.‐B. (2005). Proteasomal ATPase‐associated factor 1 negatively regulates proteasome activity by interacting with proteasomal ATPases. Molecular and Cellular Biology, 25, 3842–3853. 10.1128/mcb.25.9.3842-3853.2005 15831487 PMC1084299

[acel14128-bib-0023] Petroi, D. , Popova, B. , Taheri‐Talesh, N. , Irniger, S. , Shahpasandzadeh, H. , Zweckstetter, M. , Outeiro, T. F. , & Braus, G. H. (2012). Aggregate clearance of alpha‐synuclein in *Saccharomyces cerevisiae* depends more on autophagosome and vacuole function than on the proteasome. The Journal of Biological Chemistry, 287, 27567–27579. 10.1074/jbc.M112.361865 22722939 PMC3431624

[acel14128-bib-0024] Popova, B. , Galka, D. , Häffner, N. , Wang, D. , Schmitt, K. , Valerius, O. , Knop, M. , & Braus, G. H. (2021). α‐Synuclein decreases the abundance of proteasome subunits and alters ubiquitin conjugates in yeast. Cell, 10, 2229. 10.3390/CELLS10092229 PMC8468666 34571878

[acel14128-bib-0025] Popova, B. , Wang, D. , Pätz, C. , Akkermann, D. , Lázaro, D. F. , Galka, D. , Kolog Gulko, M. , Bohnsack, M. T. , Möbius, W. , Bohnsack, K. E. , Outeiro, T. F. , & Braus, G. H. (2021). DEAD‐box RNA helicase Dbp4/DDX10 is an enhancer of α‐synuclein toxicity and oligomerization. PLoS Genetics, 17, e1009407. 10.1371/journal.pgen.1009407 33657088 PMC7928443

[acel14128-bib-0026] Roelofs, J. , Park, S. , Haas, W. , Tian, G. , McAllister, F. E. , Huo, Y. , Lee, B. H. , Zhang, F. , Shi, Y. , Gygi, S. P. , & Finley, D. (2009). Chaperone‐mediated pathway of proteasome regulatory particle assembly. Nature, 459, 861–865. 10.1038/nature08063 19412159 PMC2727592

[acel14128-bib-0027] Saeki, Y. , Toh‐e, A. , Kudo, T. , Kawamura, H. , & Tanaka, K. (2009). Multiple proteasome‐interacting proteins assist the assembly of the yeast 19S regulatory particle. Cell, 137, 900–913. 10.1016/J.CELL.2009.05.005 19446323

[acel14128-bib-0028] Sakata, E. , Eisele, M. R. , & Baumeister, W. (2021). Molecular and cellular dynamics of the 26S proteasome. Biochimica et Biophysica Acta, Proteins and Proteomics, 1869, 140583. 10.1016/J.BBAPAP.2020.140583 33321258

[acel14128-bib-0029] Seong, K. M. , Baek, J.‐H. , Yu, M.‐H. , & Kim, J. (2007). Rpn13p and Rpn14p are involved in the recognition of ubiquitinated Gcn4p by the 26S proteasome. FEBS Letters, 581, 2567–2573. 10.1016/j.febslet.2007.04.064 17499717

[acel14128-bib-0030] Shahpasandzadeh, H. , Popova, B. , Kleinknecht, A. , Fraser, P. E. , Outeiro, T. F. , & Braus, G. H. (2014). Interplay between sumoylation and phosphorylation for protection against alpha‐synuclein inclusions. The Journal of Biological Chemistry, 289, 31224–31240. 10.1074/jbc.M114.559237 25231978 PMC4223324

[acel14128-bib-0031] Shirozu, R. , Yashiroda, H. , & Murata, S. (2015). Identification of minimum Rpn4‐responsive elements in genes related to proteasome functions. FEBS Letters, 589, 933–940. 10.1016/j.febslet.2015.02.025 25747386

[acel14128-bib-0032] Snyder, H. , Mensah, K. , Theisler, C. , Lee, J. , Matouschek, A. , & Wolozin, B. (2003). Aggregated and monomeric α‐synuclein bind to the S6′ proteasomal protein and inhibit proteasomal function. The Journal of Biological Chemistry, 278, 11753–11759. 10.1074/jbc.M208641200 12551928

[acel14128-bib-0033] Spillantini, M. G. , Schmidt, M. L. , Lee, V. M. Y. , Trojanowski, J. Q. , Jakes, R. , & Goedert, M. (1997). α‐synuclein in Lewy bodies [8]. Nature, 388, 839–840. 10.1038/42166 9278044

[acel14128-bib-0034] Stefanis, L. , Emmanouilidou, E. , Pantazopoulou, M. , Kirik, D. , Vekrellis, K. , & Tofaris, G. K. (2019). How is alpha‐synuclein cleared from the cell? Journal of Neurochemistry, 150, 577–590. 10.1111/jnc.14704 31069800

[acel14128-bib-0035] Stefanis, L. , Larsen, K. E. , Rideout, H. J. , Sulzer, D. , & Greene, L. A. (2001). Expression of A53T mutant but not wild‐type alpha‐synuclein in PC12 cells induces alterations of the ubiquitin‐dependent degradation system, loss of dopamine release, and autophagic cell death. The Journal of Neuroscience, 21, 9549–9560. 11739566 10.1523/JNEUROSCI.21-24-09549.2001 PMC6763041

[acel14128-bib-0036] Suzuki, G. , Imura, S. , Hosokawa, M. , Katsumata, R. , Nonaka, T. , Hisanaga, S. I. , Saeki, Y. , & Hasegawa, M. (2020). α‐Synuclein strains that cause distinct pathologies differentially inhibit proteasome. eLife, 9, 1–21. 10.7554/ELIFE.56825 PMC7406352 32697196

[acel14128-bib-0037] Tanaka, Y. , Engelender, S. , Igarashi, S. , Rao, R. K. , Wanner, T. , Tanzi, R. E. , Sawa, A. , L Dawson, V. , Dawson, T. M. , & Ross, C. A. (2001). Inducible expression of mutant α‐synuclein decreases proteasome activity and increases sensitivity to mitochondria‐dependent apoptosis. Human Molecular Genetics, 10, 919–926. 10.1093/hmg/10.9.919 11309365

[acel14128-bib-0038] Teng, X. , Dayhoff‐Brannigan, M. , Cheng, W. C. , Gilbert, C. E. , Sing, C. N. , Diny, N. L. , Wheelan, S. J. , Dunham, M. J. , Boeke, J. D. , Pineda, F. J. , & Hardwick, J. M. (2013). Genome‐wide consequences of deleting any single gene. Molecular Cell, 52, 485–494. 10.1016/J.MOLCEL.2013.09.026 24211263 PMC3975072

[acel14128-bib-0039] Tenreiro, S. , Reimão‐Pinto, M. M. , Antas, P. , Rino, J. , Wawrzycka, D. , Macedo, D. , Rosado‐Ramos, R. , Amen, T. , Waiss, M. , Magalhães, F. , Gomes, A. , Santos, C. N. , Kaganovich, D. , & Outeiro, T. F. (2014). Phosphorylation modulates clearance of alpha‐synuclein inclusions in a yeast model of Parkinson's disease. PLoS Genetics, 10, e1004302. 10.1371/journal.pgen.1004302 24810576 PMC4014446

[acel14128-bib-0040] Tong, A. H. Y. , & Boone, C. (2006). Synthetic genetic array analysis in *Saccharomyces cerevisiae* . Methods in Molecular Biology, 313, 171–192. 10.1385/1-59259-958-3:171 16118434

[acel14128-bib-0041] Vilchez, D. , Saez, I. , & Dillin, A. (2014). The role of protein clearance mechanisms in organismal ageing and age‐related diseases. Nature Communications, 5, 5659. 10.1038/ncomms6659 25482515

[acel14128-bib-0042] Xilouri, M. , Brekk, O. R. , & Stefanis, L. (2013). Alpha‐synuclein and protein degradation systems: A reciprocal relationship. Molecular Neurobiology, 47, 537–551. 10.1007/s12035-012-8341-2 22941029

[acel14128-bib-0043] Zavodszky, E. , Peak‐Chew, S. Y. , Juszkiewicz, S. , Narvaez, A. J. , & Hegde, R. S. (2021). Identification of a quality‐control factor that monitors failures during proteasome assembly. Science, 373, 998–1004. 10.1126/science.abc6500 34446601 PMC7611656

[acel14128-bib-0044] Zhang, N.‐Y. Y. , Tang, Z. , & Liu, C.‐W. W. (2008). Alpha‐synuclein protofibrils inhibit 26 S proteasome‐mediated protein degradation: Understanding the cytotoxicity of protein protofibrils in neurodegenerative disease pathogenesis. The Journal of Biological Chemistry, 283, 20288–20298. 10.1074/jbc.M710560200 18502751

