# Supplementary material for: Inhibition of 26S proteasome activity by α‐synuclein is mediated by the proteasomal chaperone Rpn14/PAAF1
Source: Aging Cell. 2024 Feb 28;23(5):e14128. doi: 10.1111/acel.14128 (PMC11113265; doi:10.1111/acel.14128)
Supplement: Supplementary file 1 — Data S1 – Supporting methods. [file ACEL-23-e14128-s001.pdf]

Supplementary methods

Tandem Fluorescent Protein Timer Screening and data analyses

Yeast tFT library was employed that consists of 4044 strains each expressing a different tFT-tagged protein (Khmelinskii *et al.* 2014). Three query strains were constructed that harbor two genomically integrated copies of *GAL1*-driven $SNCA^{WT}$, $SNCA^{S129A}$ gene or empty vector (EV) control, respectively. The strains RH3906, RH3907 and RH3905 were generated by integrative transformation of yeast strain yMaM1234 with the linearized integrative plasmids pME5515, pME5516 or pME5514, respectively, using standard lithium acetate procedure (Gietz and Woods 2002). The integrative plasmids carry *kanMX* cassette conferring G418 resistance, and a 200 bp genomic sequence that enables integration into the *Δura3* locus. The number of integrated copies was verified by Southern blotting as previously described (Petroi *et al.* 2012). The query strains were mated with the tFT library in a 1536-colony format, with three technical replicates that were arranged next to each other on the solid agar plates. Synthetic genetic array (SGA) procedure (Baryshnikova *et al.* 2010) was applied, and each query strain was crossed to an array of tFT clones in a high-throughput scale with a ROTOR HDA pinning robot (Singer instruments, UK). Briefly, tFT and query strains were mated, the resulting diploids were selected, sporulated, and selected for haploids carrying the tFT-tagged ORF and the query allele through several replica-pinning steps on appropriate selective media as described previously (Khmelinskii *et al.* 2014), followed by seamless *URA3* marker excision.

For high-throughput fluorescence intensity measurements yeast cells were pinned on galactose-containing plates to induce *GAL1*-driven αSyn expression and the plates were grown for 24 h at 30°C. Fluorescence measurements were conducted with a stacker for automated plate delivery to Infinite M1000 Pro plate reader (Tecan, Switzerland) in a custom temperature control chamber. Intensities were recorded in the mCherry and in the sfGFP channel. The plates were imaged with a PhenoBooth Colony Counter (Singer instruments, UK). Failed crosses after haploid selection were excluded from the measurement based on colony size.

The recorded fluorescence intensities were corrected for background by subtracting the median local autofluorescence of the EV control arranged next to each sample. Fluorescence intensity measurements were log-transformed. Spatial effect normalization was performed to correct GFP and mCherry signals as a function of

position. The data for each plate were normalized to the median fluorescence of a reference strain set that was present on every plate. Standard deviations were regressed against absolute fluorescence intensities.

For each single strain the normalized and corrected intensities were used to calculate the ratio mCherry/sfGFP: R=log2(mCherry/GFP). Changes in protein stability between the control (EV) and aSyn or S129A expressing strains were estimated by subtracting the log-ratios of mCherry/sfGFP intensities yielding a Δ-score ($RatioDiff_{(EV-\alpha Syn)} = log2(R_{EV}) - log2(R_{\alpha Syn})$). Delta-scores of negative values indicate stabilization of the tFT-fusion protein upon αSyn expression, while positive values indicate destabilization of the tFT-fusion protein compared to the EV control. *P*-values were calculated with a moderated t-test and adjusted with the Benjamini-Hochberg method for controlling the false discovery rate. Significant differences in protein stability were acknowledged when $p \leq 0.01$.

The intensity of the GFP signal that is a measure of protein abundance was compared with the median background intensity (bg) of GFP, and the fusion proteins were categorized into three expression groups according to the ratio Rb = GFP/bg: (i) low expression level (Rb = 1 to 3); (ii) middle expression level (Rb = 3 to 10); (iii) high expression level (Rb = 10 to 350). To select hits with significantly altered protein stability, the following criteria were used: p<0.01; cut-off delta-score: low expression level: -2.3; medium expression level: -1; high expression level: -0.5 (Table S1 and Table S2).

Cycloheximide chases

Yeast strains were grown at 30°C in galactose-containing SC medium for 6 h to A_{600} = =0.8. Afterwards, cycloheximide was added to a final concentration of 50 µg/ml. Three-milliliter samples were taken at each time point, cells were harvested by centrifugation and flash frozen in liquid nitrogen. Whole-cell extracts were prepared as previously described (Knop *et al.* 1999) and subjected to western blot analysis.

Purification of 26S Proteasomes

The intact 26S proteasomes were purified via *RPN11-3xFLAG* tag. *S. cerevisiae* cells were grown for 16 h in galactose-containing SC-selection medium for induction of αSyn expression. The purification of 3XFLAG-tagged 26S proteasomes was performed in two steps. Cell lysis was performed by cryo-grinding, followed by affinity purification

using M2 anti-FLAG beads (Sigma A2220). After incubation for 1.5 h at 4ºC, the proteasome was eluted with FLAG peptide (MedChemExpress, HY-P0223). Purified proteasomes were loaded on linear 15–45% (w/w) sucrose gradients in buffer containing 20 mM HEPES-NaOH, pH 7.5, 40 mM NaCl, 5 mM $MgCl_2$, 4 mM ATP, 5 mM DTT, centrifuged at 28,000 rpm for 16 h at 4°C with Optima XPN-80 and SW40Ti swing rotor (Beckman Coulter), fractionated, and analyzed by Western blot.

References

Baryshnikova A., Costanzo M., Dixon S., Vizeacoumar F. J., Myers C. L., Andrews B., Boone C. (2010) *Synthetic genetic array (SGA) analysis in Saccharomyces cerevisiae and schizosaccharomyces pombe*. Elsevier Inc.

Gietz R. D., Woods R. A. (2002) Transformation of yeast by lithium acetate/single-stranded carrier DNA/polyethylene glycol method. *Methods Enzymol.* **350**, 87–96.

Khmelinskii A., Blaszczak E., Pantazopoulou M., Fischer B., Omnus D. J., Dez G. Le, Brossard A., et al. (2014) Protein quality control at the inner nuclear membrane. *Nature* **516**, 410–413.

Knop M., Siegers K., Pereira G., Zachariae W., Winsor B., Nasmyth K., Schiebel E. (1999) Epitope tagging of yeast genes using a PCR-based strategy: more tags and improved practical routines. *Yeast* **15**, 963–972.

Petroi D., Popova B., Taheri-Talesh N., Irniger S., Shahpasandzadeh H., Zweckstetter M., Outeiro T. F., Braus G. H. (2012) Aggregate clearance of alpha-synuclein in Saccharomyces cerevisiae depends more on autophagosome and vacuole function than on the proteasome. *J Biol Chem* **287**, 27567–27579.
