# Supplementary material for: Inhibition of 26S proteasome activity by α‐synuclein is mediated by the proteasomal chaperone Rpn14/PAAF1
Source: Aging Cell. 2024 Feb 28;23(5):e14128. doi: 10.1111/acel.14128 (PMC11113265; doi:10.1111/acel.14128)
Supplement: Supplementary file 2 — Figures S1–S11. [file ACEL-23-e14128-s003.pdf]

Supplementary Figures S1-S11

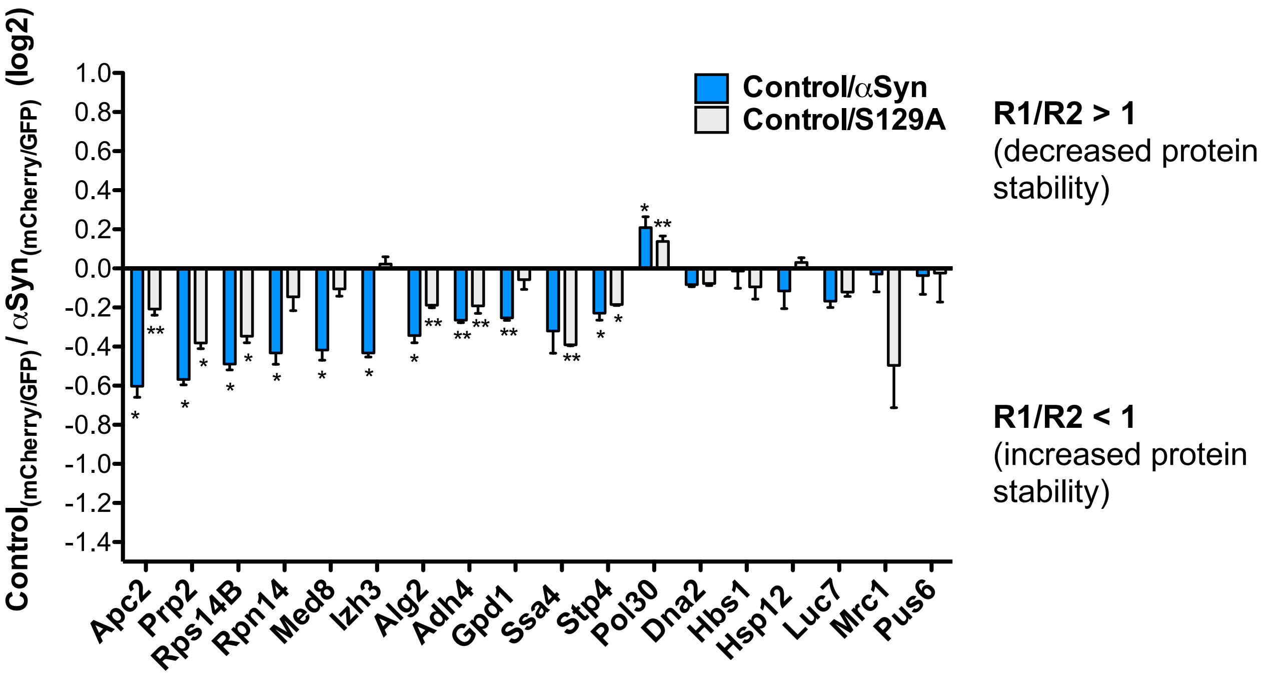

Figure S1. Fluorescence intensity ratios from flow cytometry data support the results obtained in tFT-screen. Flow cytometry was conducted with selected tFT-strains expressing αSyn, S129A or with empty vector control. Intensity of fluorescence signal derived from mCherry and sfGFP was measured for 10000 single cells and mCherry/sfGFP ratios calculated for control, αSyn or S129A expressing cells. R1/R2 indicates ratio mCherry/GFP of control cells (R1) to ratio mCherry/GFP of αSyn or S129A expressing cells (R2) as log2. Significance of differences was calculated with t-test versus control cells (*p < 0.05; **p < 0.01; ***; p < 0.001, n=3).

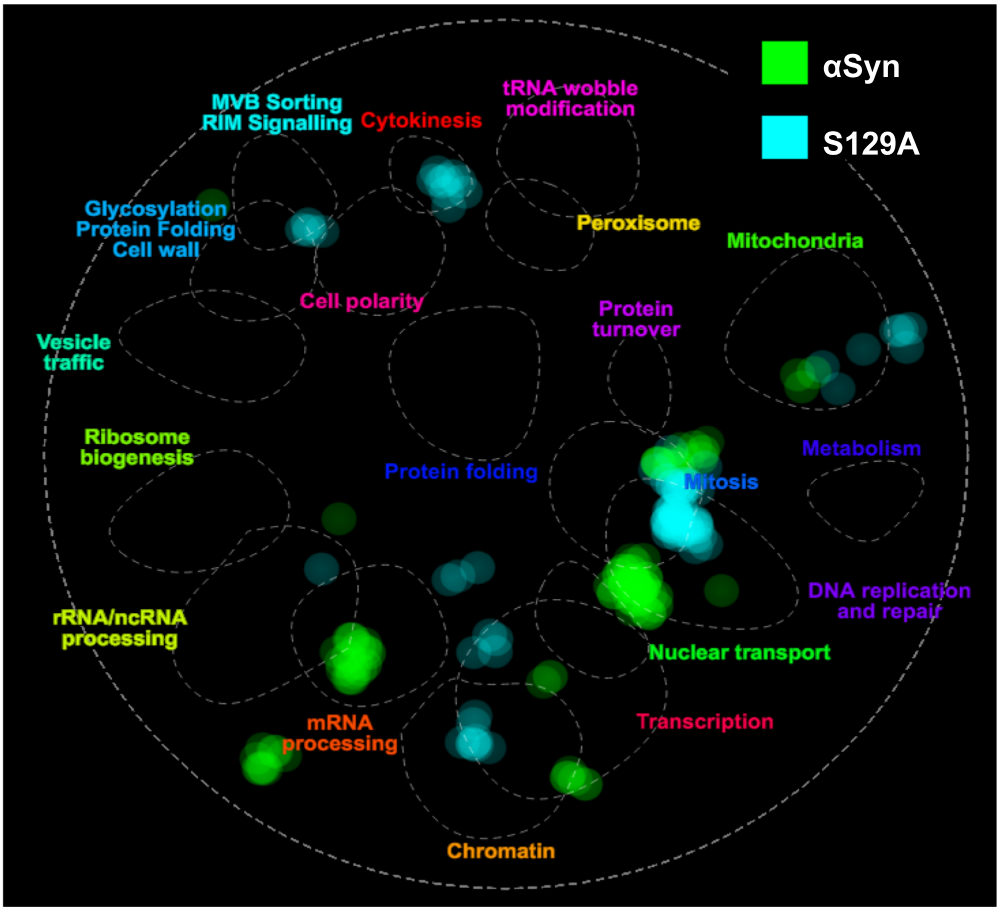

Figure S2. Spatial Analysis of Functional Enrichment (SAFE) (http://www.thecellmap.org) analysis of the identified proteins with significantly changed stability in presence of αSyn or S129A, compared to empty vector control. Specific biological processes that are enriched upon αSyn expression (green) or S129A expression (blue) are depicted on the network map. αSyn expression revealed enrichment of proteins involved in DNA replication & repair, mitosis, mRNA processing, nuclear transport, transcription and mitochondria. In presence of S129A the hits are functionally enriched mainly in the categories of DNA replication & repair and glycosylation & protein folding.

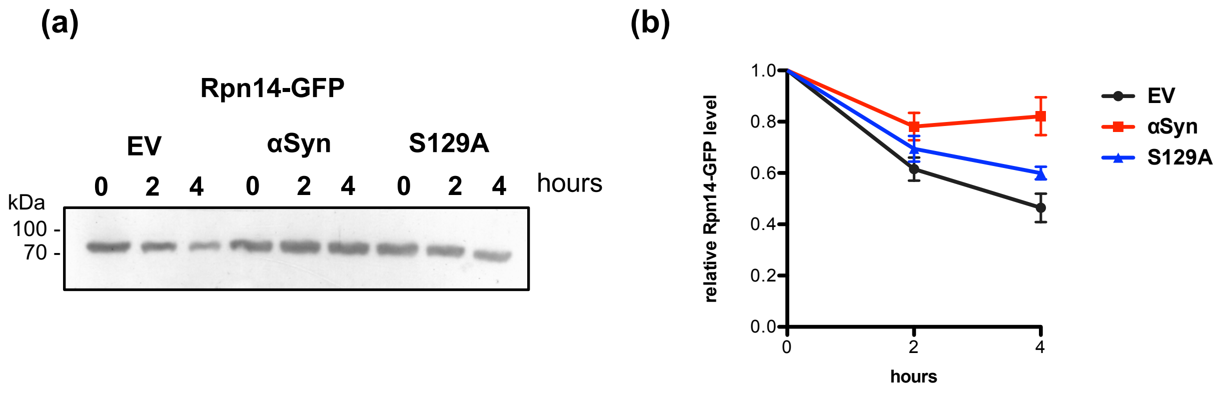

Figure S3. Expression of αSyn increases Rpn14 stability. (a) Cycloheximide chase experiments with *RPN14*-GFP strain, expressing *GAL1*-driven αSyn, S129A or empty vector control. Cells were grown for 6 h in galactose-containing medium prior to treatment with 50 µg/ml cycloheximide to stop *de novo* protein synthesis. Equal volume of samples were collected at the indicated time points and flash frozen in liquid nitrogen. Whole-cell extracts were separated by SDS–PAGE followed by immunoblotting with GFP antibody. (b) Densitometric analysis of the immunodetection of Rpn14-GFP. The GFP signal was normalized to the signal intensity of each individual sample at 0 h.

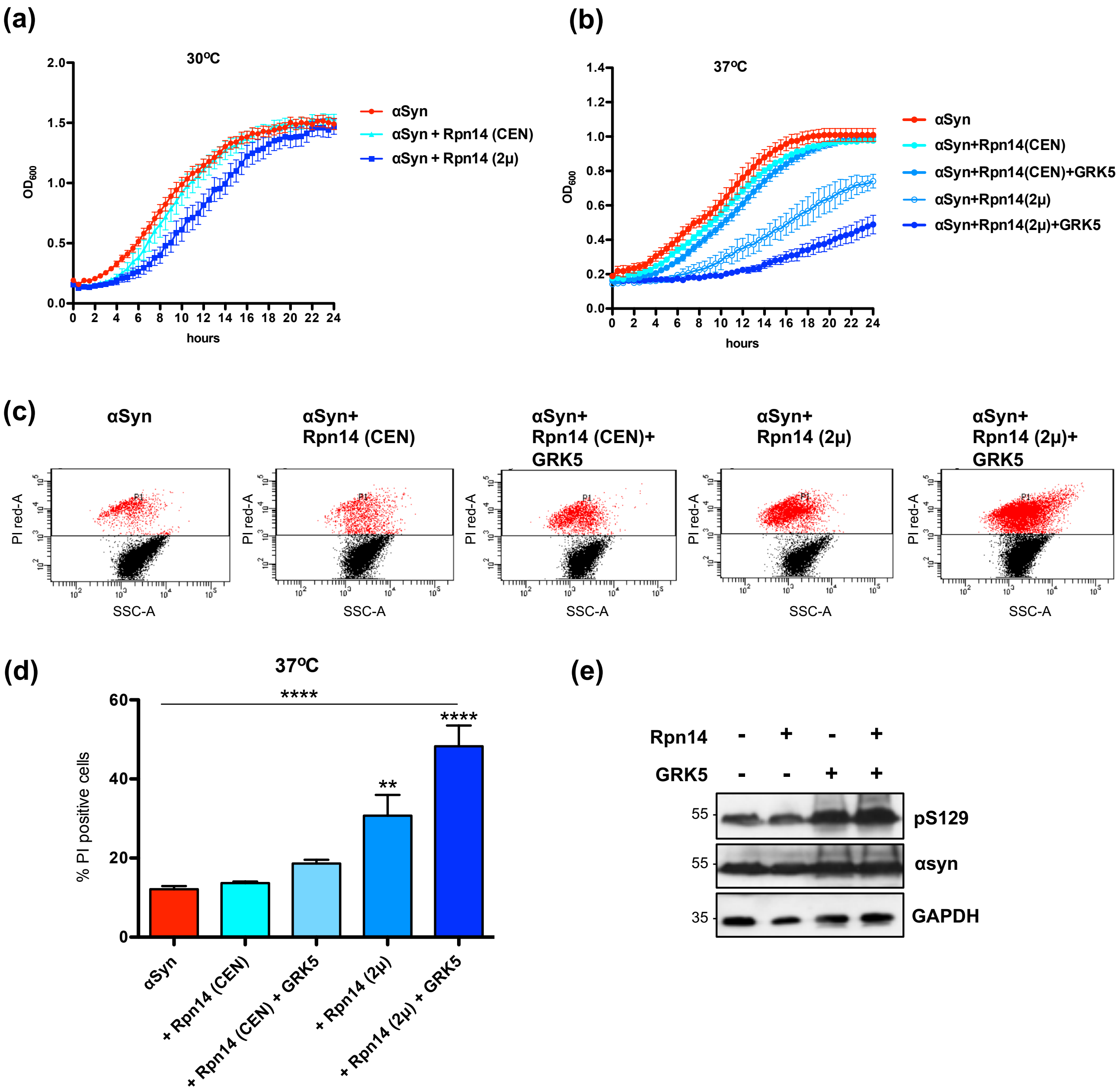

Figure S4. Elevated levels of Rpn14 increase αSyn-associated growth retardation and cytotoxicity. (a) Growth assays of yeast cells, expressing *GAL1*-driven αSyn-GFP from two gene copies, and *RPN14* from a low copy *CEN* plasmid or a high copy 2μ plasmid at 30°C. (b) Growth assays of yeast cells from (a) at 37°C with or without overexpression of the human kinase GRK5. (c) Propidium iodide (PI) fluorescence intensity and side scatter (SSC) of cells assessed with flow cytometry. Representative charts illustrating the sub-populations of yeast cells with higher fluorescent intensities (P1) than the background. Cells were stained with 12.5 μg/ml PI for 30 min. 6 h after induction of expression at 37°C (d) Percentage of PI positive cells from (c). A total of 10000 cells per experiment were counted. Significance of differences was calculated with one-way ANOVA with Newman–Keuls post-hoc test (****, $p < 0.0001$; ***, $p < 0.001$; **, $p < 0.01$; $n = 3$). (e) Expression of the human kinase GRK5 increases S129 phosphorylation in yeast. Immunoblotting of yeast cells expressing αSyn-GFP from two copies in presence or absence of *RPN14* or *GRK5* overexpression. The phosphorylation level of αSyn was detected by pS129 phosphorylation-specific antibody when expressed either alone or in the presence of GRK5.

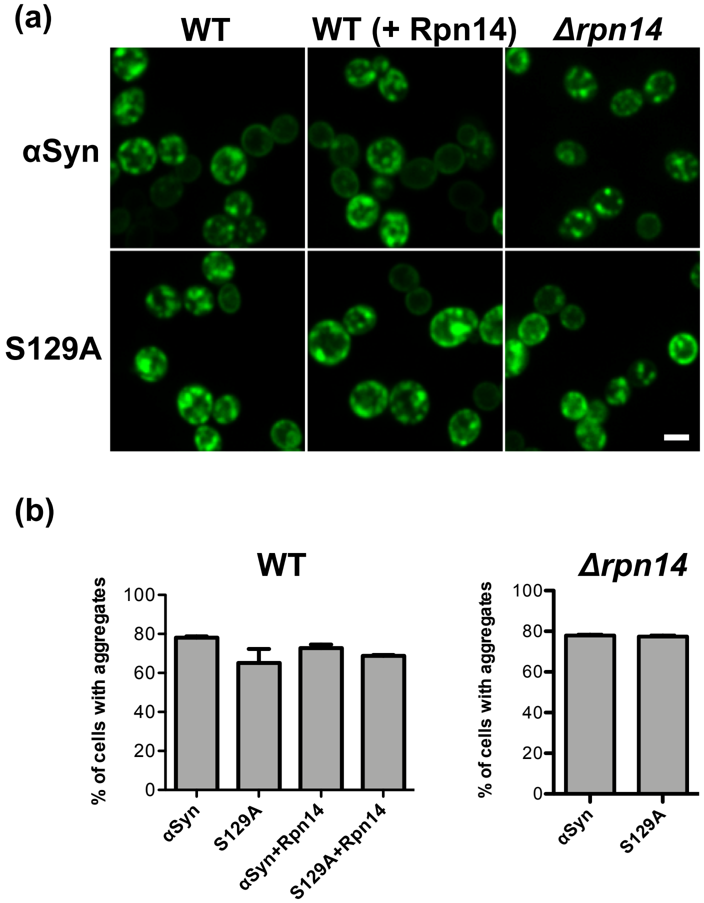

Figure S5. Rpn14 does not affect αSyn aggregates formation. (a) Fluorescence microscopy of yeast cells, expressing *GAL1*-driven αSyn-GFP or S129A-GFP from 2μ plasmids 6 h post-induction in wild type yeast strain at endogenous (WT) or elevated (WT + Rpn14) levels of Rpn14, or in *Δrpn14* deletion strain. Rpn14 was overexpressed from 2μ plasmid. Scale bar: 5 μm. (b) Quantification of the percentage of cells displaying αSyn-GFP or S129A-GFP aggregates from (a) (n=3).

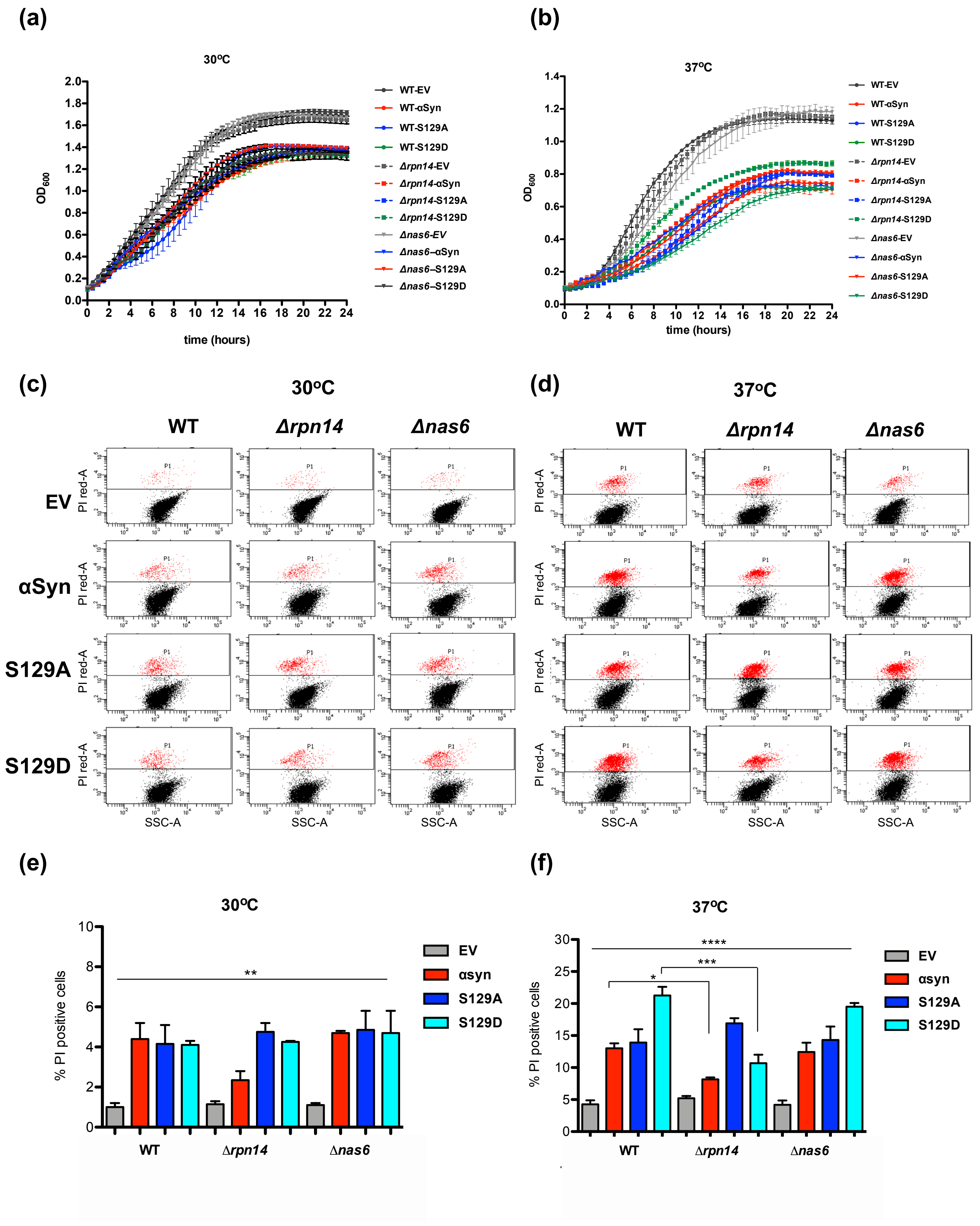
(a)
30ºC
OD600
time (hours)
WT-EV
WT-αSyn
WT-S129A
WT-S129D
Δrpn14-EV
Δrpn14-αSyn
Δrpn14-S129A
Δrpn14-S129D
Δnas6-EV
Δnas6–αSyn
Δnas6–S129A
Δnas6–S129D
(b)
37ºC
OD600
time (hours)
WT-EV
WT-αSyn
WT-S129A
WT-S129D
Δrpn14-EV
Δrpn14-αSyn
Δrpn14-S129A
Δrpn14-S129D
Δnas6-EV
Δnas6-αSyn
Δnas6-S129A
Δnas6-S129D
(c)
30ºC
WT
Δrpn14
Δnas6
EV
αSyn
S129A
S129D
PI red-A
SSC-A
P1
(d)
37ºC
WT
Δrpn14
Δnas6
PI red-A
SSC-A
P1
(e)
30ºC
% PI positive cells
**
WT
Δrpn14
Δnas6
EV
αsyn
S129A
S129D
(f)
37ºC
% PI positive cells
****
***
*
WT
Δrpn14
Δnas6
EV
αsyn
S129A
S129D

Figure S6. Native expression of Rpn14 increases αSyn toxicity. Growth assays of yeast cells, expressing αSyn, S129A or S129D in wild type, *Δrpn14 or Δnas6* strains at 30°C (a) or 37°C (b). (c) Propidium iodide (PI) fluorescence intensity and side scatter (SSC) of cells assessed with flow cytometry analysis. Representative flow cytometry charts illustrating the sub-populations of yeast cells with higher fluorescent intensities (P1) than the background. Cells expressing different αSyn variants or empty vector (EV) control after 6 h induction of expression at 30°C (c) or at 37°C (d) were stained with 12.5 µg/ml PI for 30 min. The percentage of PI positive cells at 30°C (e) or at 37°C (f) is presented. A total of 10000 cells per experiment were counted. Significance of differences was calculated with one-way ANOVA with Newman–Keuls post-hoc test (****, $p < 0.0001$; ***, $p < 0.001$; **, $p < 0.01$; *, $p < 0.05$; $n = 3$).

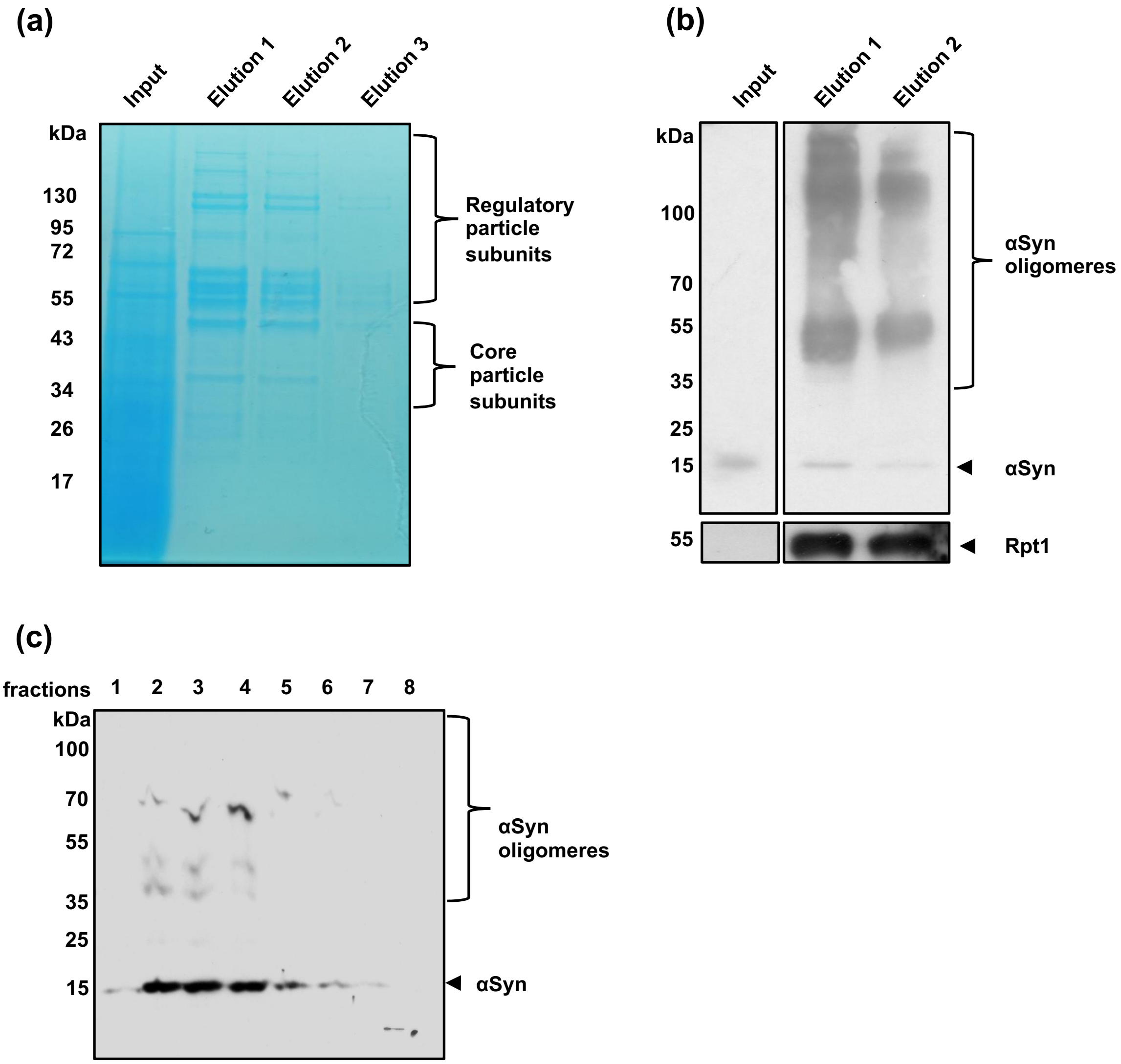

Figure S7. αSyn copurifies with Rpn11-3xFLAG tagged 26S yeast proteasome. (a) SDS-PAGE of Input (crude protein extract) and Elutions 1-3. αSyn expression was induced for 16 hours. Cells were harvested and crude protein extract prepared as indicated in Materials and Methods (Input). Bound 26S proteasomes were eluted three times with 250 µL of 400 µg/mL Flag peptide. Equal volume from each sample was loaded on the gel. The profile of the eluted proteins is typical for the 26S proteasome landscape. **(b)** Western blot of samples from 3xFLAG pulldown. Membrane was probed with anti αSyn antibody, stripped and re-probed with anti Rpt1 antibody. **(c)** Western blot analysis of ultracentrifugation fractions. 20 µl from each fraction were loaded on the gel. Membrane was probed with anti αSyn antibody. Signal was detected in fractions 2-6.

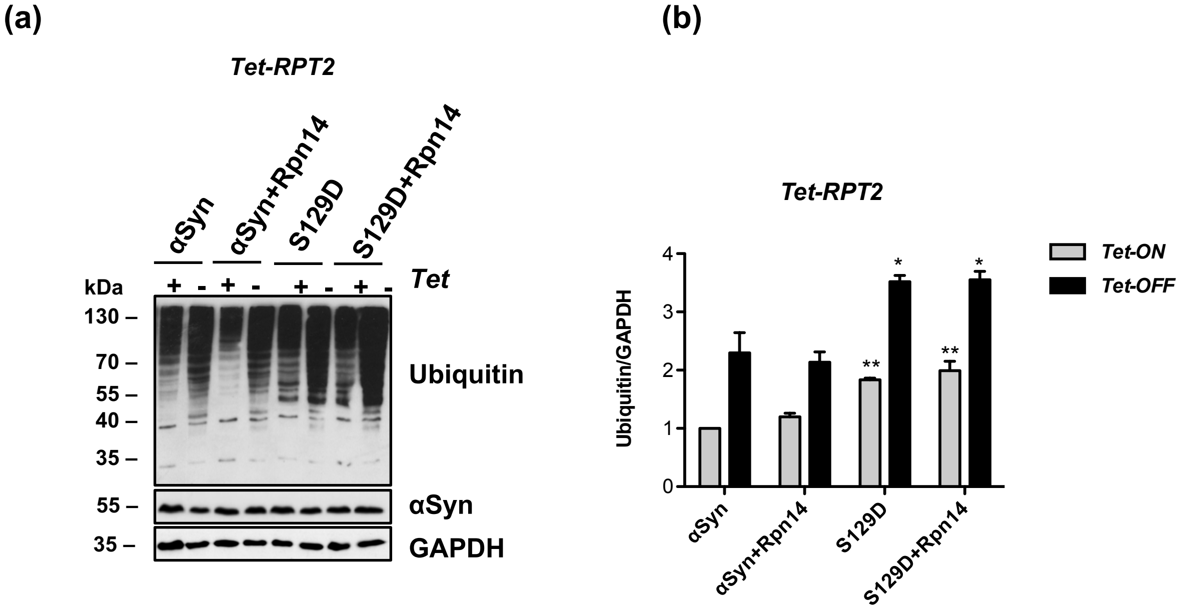

Figure S8. Expression of S129D increases the accumulation of ubiquitinated proteins. (a) Immunoblot analysis of *Tet-RPT2* strain expressing *GPD*-driven *RPN14*, *GAL1*-driven αSyn-GFP or S129D-GFP. Yeast cells were grown overnight in galactose-containing medium to induce αSyn expression. The *Tet* promoter was repressed by simultaneous addition of 10 μg/ml doxycycline to the growth medium. (+) indicates *Tet-ON*, and (-) *Tet-OFF*. Immunoblotting analyses were performed with anti-ubiquitin or αSyn antibodies. GAPDH was used as a loading control. (b) Densitometric analysis of the immunodetection of the ubiquitin conjugates in *Tet-RPT2* strain relative to GAPDH. Significance of differences was calculated with *t*-test relative to the corresponding αSyn +/- Rpn14 (*, $p < 0.05$; **, $p < 0.01$).

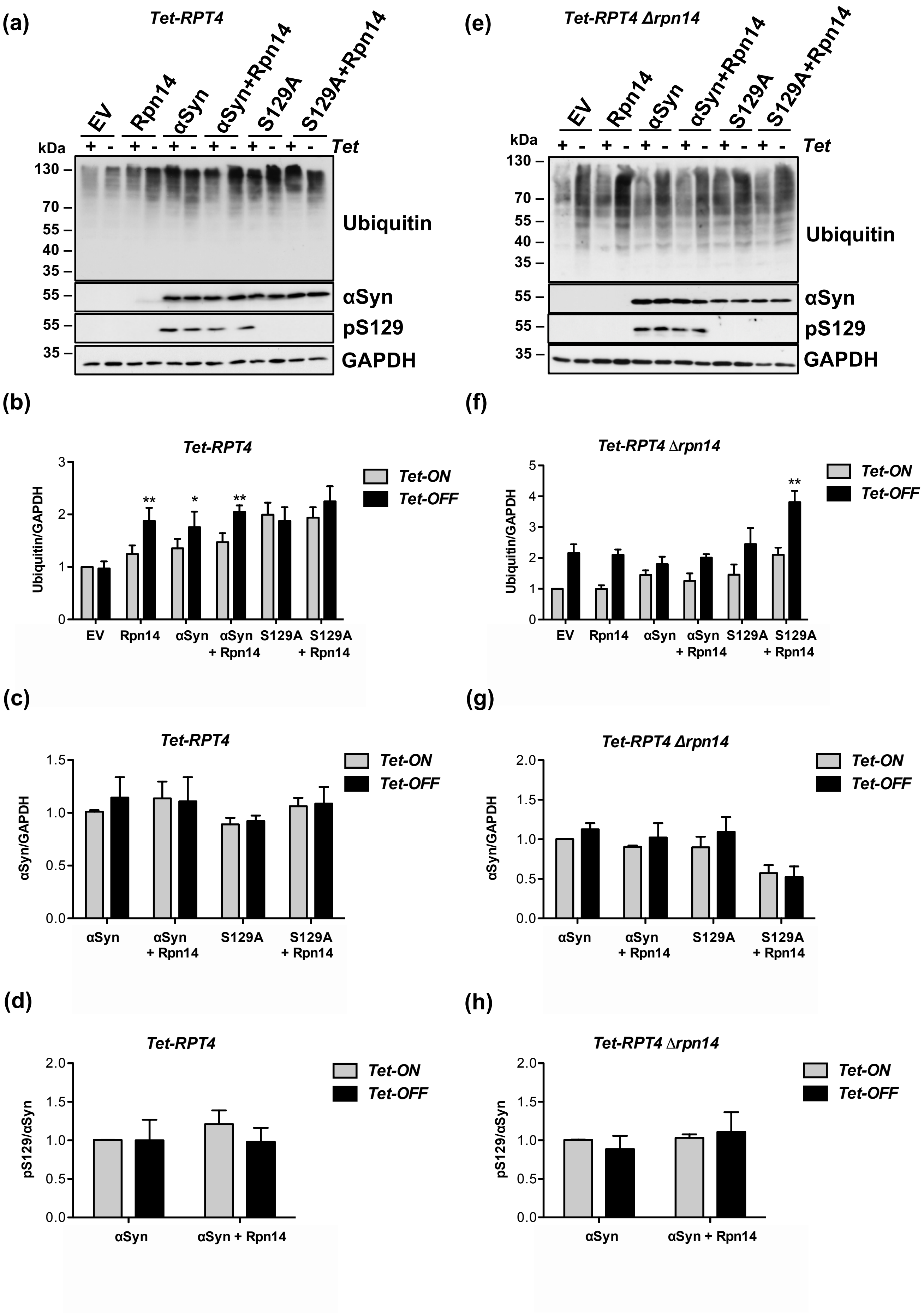

(a)
Tet-RPT4
EV
Rpn14
αSyn
αSyn+Rpn14
S129A
S129A+Rpn14
kDa
Tet
130
70
55
40
35
Ubiquitin
αSyn
pS129
GAPDH
(e)
Tet-RPT4 Δrpn14
(b)
Tet-RPT4
Ubiquitin/GAPDH
Tet-ON
Tet-OFF
**
*
+ Rpn14
(f)
Tet-RPT4 Δrpn14
(c)
αSyn/GAPDH
(g)
(d)
pS129/αSyn
αSyn + Rpn14
(h)

Figure S9. Expression of αSyn or elevated level of Rpn14 leads to accumulation of ubiquitinated proteins upon downregulation of *Tet-RPT4*. (a) Immunoblot analysis of *Tet-RPT4* strain expressing *GPD*-driven *RPN14* from *CEN* plasmid, *GAL1*-driven αSyn-GFP or S129A-GFP. Empty vector (EV) was used as control. Yeast cells were grown overnight in a galactose-containing medium to induce αSyn expression. The *Tet* promoter was downregulated by simultaneous addition of 10 μg/mL doxycycline (Dox) to the growth medium. (+) indicates *Tet-ON,* and (-) indicates *Tet-OFF*. Immunoblotting analysis was performed with anti-ubiquitin, αSyn or pS129 antibodies. GAPDH was used as loading control. (b) Densitometric analysis of the immunodetection of the ubiquitin conjugates in *Tet-RPT4* strain relative to GAPDH. The significance of differences was calculated with a *t*-test relative to EV control (*, $p < 0.05$; **, $p < 0.01$). (c) Densitometric analysis of αSyn protein levels from *Tet-RPT4* (A) relative to the GAPDH loading control. (d) Densitometric analysis of pS129 fraction relative to αSyn signal. (e) Immunoblot analysis of *Tet-RPT4 Δrpn14* strain expressing αSyn-GFP, S129A-GFP, *RPN14* or empty vector (EV) as control, performed as in (a). (f) Densitometric analysis of the immunodetection of the ubiquitin conjugates in *Tet-RPT4 Δrpn14* strain relative to GAPDH. The significance of differences was calculated with *t*-test relative to EV control (**, $p < 0.01$). (g) Densitometric analysis of αSyn protein levels from (e) relative to GAPDH. (h) Densitometric analysis of pS129 fraction relative to αSyn signal in *Tet-RPT4 Δrpn14* strain.

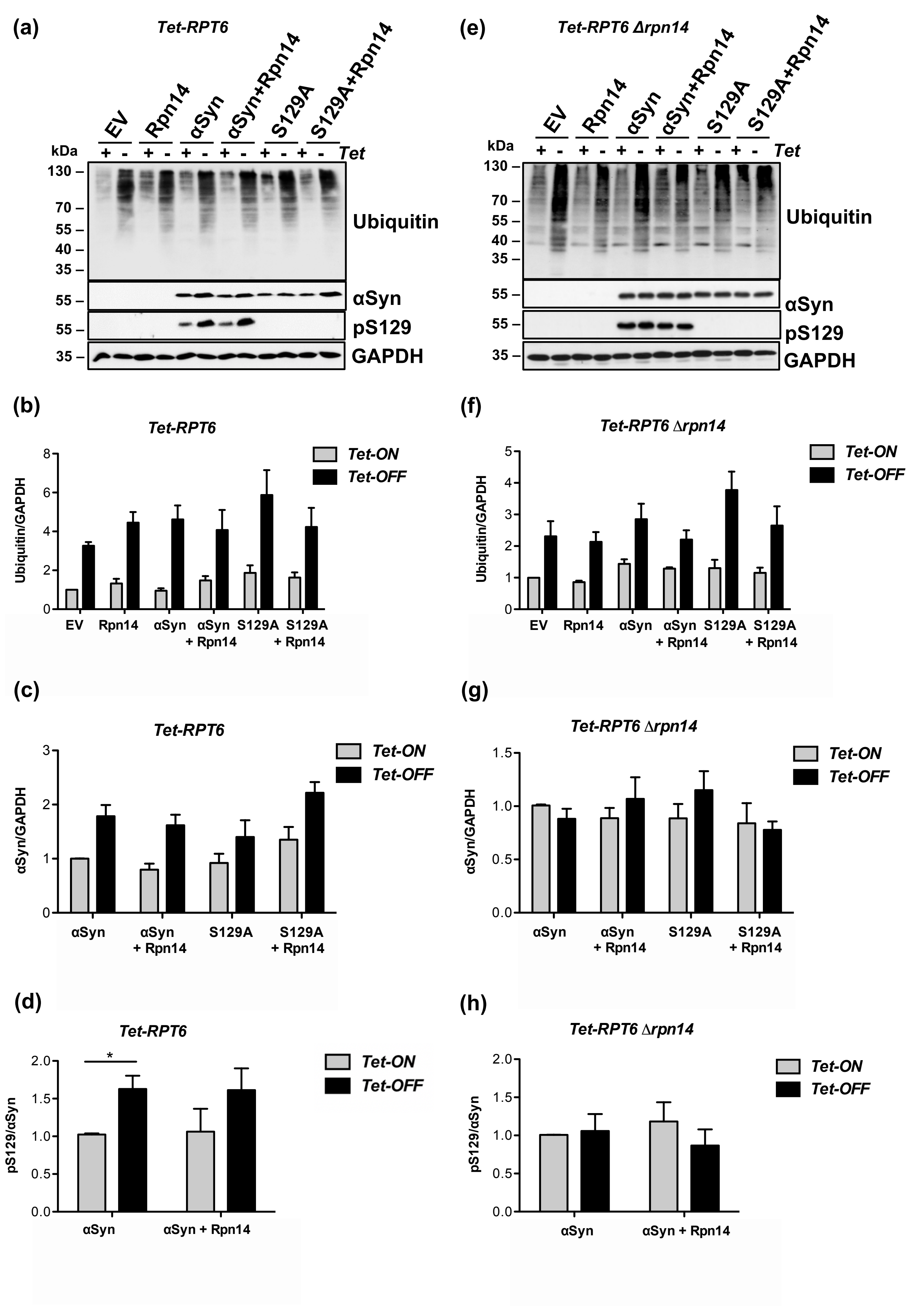

(a)
Tet-RPT6
EV
Rpn14
αSyn
αSyn+Rpn14
S129A
S129A+Rpn14
kDa
+ - + - + - + - + - + -
Tet
130
70
55
40
35
Ubiquitin
55
αSyn
55
pS129
35
GAPDH
(e)
Tet-RPT6 Δrpn14
EV
Rpn14
αSyn
αSyn+Rpn14
S129A
S129A+Rpn14
kDa
+ - + - + - + - + - + -
Tet
130
70
55
40
35
Ubiquitin
55
αSyn
55
pS129
35
GAPDH
(b)
Tet-RPT6
Tet-ON
Tet-OFF
Ubiquitin/GAPDH
0
2
4
6
8
EV
Rpn14
αSyn
αSyn + Rpn14
S129A
S129A + Rpn14
(f)
Tet-RPT6 Δrpn14
Tet-ON
Tet-OFF
Ubiquitin/GAPDH
0
1
2
3
4
5
EV
Rpn14
αSyn
αSyn + Rpn14
S129A
S129A + Rpn14
(c)
Tet-RPT6
Tet-ON
Tet-OFF
αSyn/GAPDH
0
1
2
3
αSyn
αSyn + Rpn14
S129A
S129A + Rpn14
(g)
Tet-RPT6 Δrpn14
Tet-ON
Tet-OFF
αSyn/GAPDH
0.0
0.5
1.0
1.5
αSyn
αSyn + Rpn14
S129A
S129A + Rpn14
(d)
Tet-RPT6
*
Tet-ON
Tet-OFF
pS129/αSyn
0.0
0.5
1.0
1.5
2.0
αSyn
αSyn + Rpn14
(h)
Tet-RPT6 Δrpn14
Tet-ON
Tet-OFF
pS129/αSyn
0.0
0.5
1.0
1.5
2.0
αSyn
αSyn + Rpn14

Figure S10. Expression of αSyn or elevated level of Rpn14 leads to accumulation of ubiquitinated proteins upon downregulation of *Tet-RPT6*. (a) Immunoblot analysis of *Tet-RPT6* strain expressing *GPD*-driven *RPN14* from *CEN* plasmid, *GAL1*-driven αSyn-GFP or S129A-GFP. Empty vector (EV) was used as control. Yeast cells were grown overnight in a galactose-containing medium to induce αSyn expression. The *Tet* promoter was downregulated by simultaneous addition of 10 µg/mL doxycycline (Dox) to the growth medium. (+) indicates *Tet-ON*; (-) *Tet-OFF*. Immunoblotting analysis was performed with ubiquitin, αSyn or pS129 antibodies. GAPDH was used as a loading control. (b) Densitometric analysis of the immunodetection of the ubiquitin conjugates in *Tet-RPT6* strain relative to GAPDH. (c) Densitometric analysis of αSyn protein levels from *Tet-RPT6* (a) relative to the GAPDH loading control. (d) Densitometric analysis of pS129 fraction relative to αSyn signal. The significance of differences was calculated with *t*-test relative to EV control (*, $p < 0.05$). (e) Immunoblot analysis of *Tet-RPT6 Δrpn14* strain expressing αSyn-GFP, S129A-GFP, *RPN14* or empty vector (EV) as control, performed as in (a). (f) Densitometric analysis of the immunodetection of the ubiquitin conjugates in *Tet-RPT6 Δrpn14* strain relative to GAPDH. (g) Densitometric analysis of αSyn protein levels from (e) relative to GAPDH. (h) Densitometric analysis of pS129 fraction relative to αSyn signal in *Tet-RPT6 Δrpn14* strain.

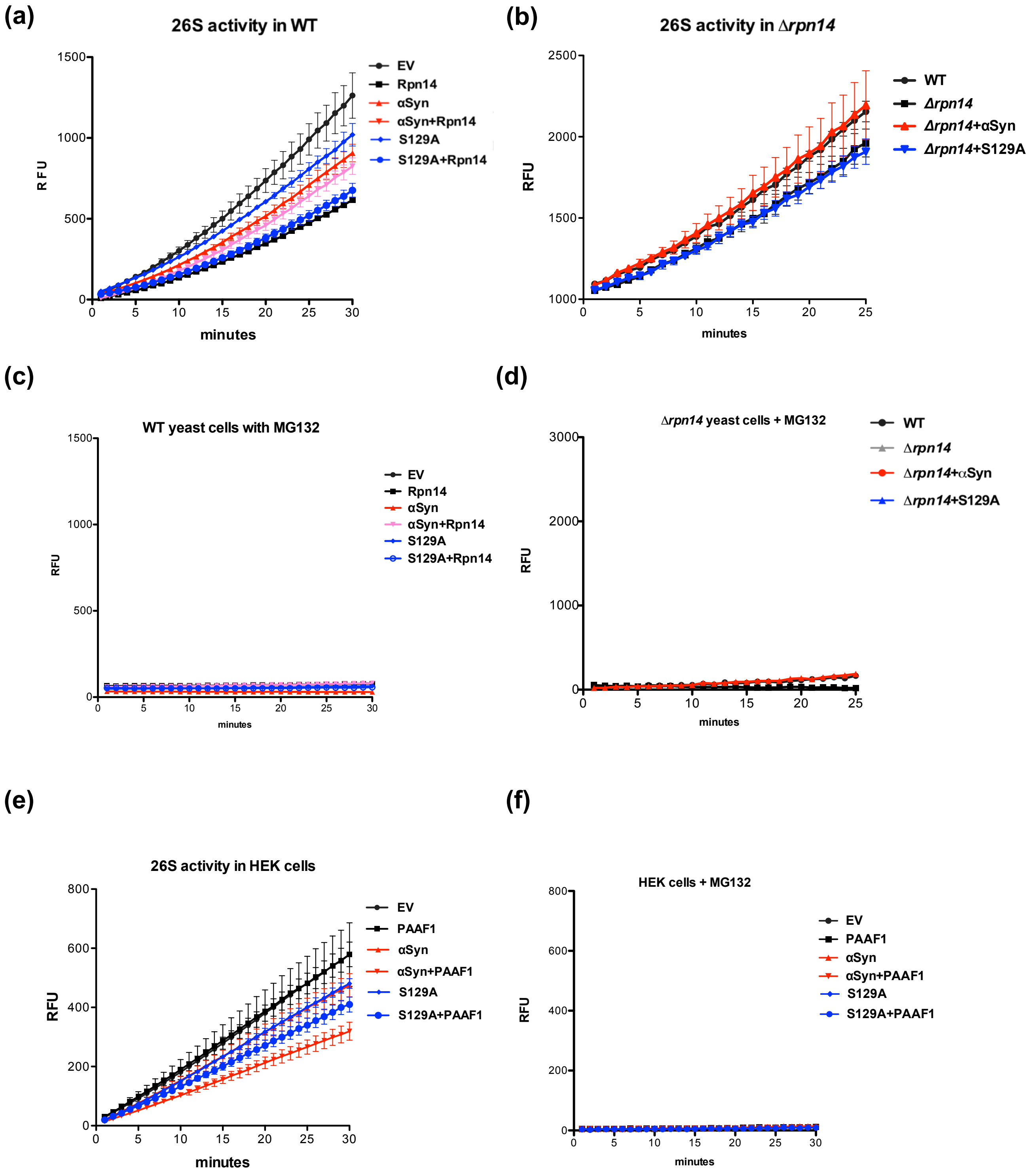

(a)
26S activity in WT
R F U
minutes
EV
Rpn14
αSyn
αSyn+Rpn14
S129A
S129A+Rpn14
(b)
26S activity in Δrpn14
RFU
minutes
WT
Δrpn14
Δrpn14+αSyn
Δrpn14+S129A
(c)
WT yeast cells with MG132
RFU
minutes
EV
Rpn14
αSyn
αSyn+Rpn14
S129A
S129A+Rpn14
(d)
Δrpn14 yeast cells + MG132
RFU
minutes
WT
Δrpn14
Δrpn14+αSyn
Δrpn14+S129A
(e)
26S activity in HEK cells
RFU
minutes
EV
PAAF1
αSyn
αSyn+PAAF1
S129A
S129A+PAAF1
(f)
HEK cells + MG132
RFU
minutes
EV
PAAF1
αSyn
αSyn+PAAF1
S129A
S129A+PAAF1

Figure S11. Proteasome activity assays. (a) Wild type (WT) yeast cells expressing *GPD*-driven *RPN14* from *CEN* plasmid, *GAL1*-driven αSyn or S129A from 2µ plasmid, or empty vector (EV) as control were collected after 6 hours of *GAL1* induction. The 26S proteasomal activity in crude protein extracts was monitored by measuring the hydrolysis of the fluorogenic peptide Suc-LLVY-AMC by detecting relative fluorescence units (RFU). (b) 26S proteasomal activity in *Δrpn14* strain, performed similarly as in (a). (c) Crude protein extracts from wild type cells or *Δrpn14* cells (d) were preincubated with 100 µM proteasome inhibitor MG132 for 10 min prior to measurement as control. Lack of proteasome activity is indicative for the specificity of the assay. (e) HEK cells were transfected with constructs expressing *PAAF1*, αSyn or S129A under the control of the *CMV* promoter. EV – empty vector. The 26S proteasomal activity was assayed in crude protein extracts by measuring the hydrolysis of Suc-LLVY-AMC. (f) HEK protein extracts were preincubated with 100 µM proteasome inhibitor MG132 for 10 min prior to the measurement presented in (e).
