# Supplementary material for: Inhibition of 26S proteasome activity by α‐synuclein is mediated by the proteasomal chaperone Rpn14/PAAF1
Source: Aging Cell. 2024 Feb 28;23(5):e14128. doi: 10.1111/acel.14128 (PMC11113265; doi:10.1111/acel.14128)
Supplement: Supplementary file 3 — Tables S1–S5. [file ACEL-23-e14128-s002.pdf]

Table S1. Yeast strains used in this study.

Name	Genotype	Source
W303	*MATa; ura3-52; trp1D2; leu2-3_112; his3-11; ade2-1; can1-100*	ATTC: 208353
BY4741	*MATa; his3Δ 1; leu2Δ0; met15Δ0; ura3Δ0*	EUROSCARF
Δrpn14	*MATa; his3Δ1; leu2Δ0; met15Δ0; ura3Δ0; Δrpn14::kanMX*	EUROSCARF
Δnas6	*MATa; his3Δ1; leu2Δ0; met15Δ0; ura3Δ0; Δnas6::kanMX*	EUROSCARF
YMaM330	*MATalpha his3Δ1 leu2Δ0 met15Δ0 ura3Δ0 can1Δ:: STE2pr-spHIS5 lyp1Δ::STE3pr-LEU2 leu2Δ::GAL1pr-I-SCEI-natNT2*	(Khmelinskii *et al*, 2014)
YMaM1234	*leu2Δ0::hphNT1-MAL63-Pmal32-NLS-SceI in BY4741*	(Khmelinskii *et al*, 2014)
YMaM1235	his3Δ1::mCherryΔN-ScURA3-mCherryΔN in YMaM330	This study
RH3905	*ura3Δ0::kanMX* in YMaM1234	This study
RH3906	*ura3Δ0::kanMX::GAL1^{pr}::SNCA^{WT}::CYC1^{term}* (two gene copies) in YMaM1234	This study
RH3907	*ura3Δ0::kanMX::GAL1^{pr}::SNCAS^{129A}::CYC1^{term}* (two gene copies) in YMaM1234	This study
RH3908	*RPN14::mCherry::sfGFP in* yMaM330	This study
RH3909	*SSA4::mCherry::sfGFP in* yMaM330	This study
RH3910	*HSP12::mCherry::sfGFP in* yMaM330	This study
RH3911	*ADH4::mCherry::sfGFP in* yMaM330	This study
RH3912	*GPD1::mCherry::sfGFP in* yMaM330	This study
RH3913	*POL30::mCherry::sfGFP in* yMaM330	This study
RH3914	*STP4::mCherry::sfGFP in* yMaM330	This study
RH3915	*LUC7::mCherry::sfGFP in* yMaM330	This study
RH3916	*MED8::mCherry::sfGFP in* yMaM330	This study
RH3917	*ALG2::mCherry::sfGFP in* yMaM330	This study
RH3918	*IZH3::mCherry::sfGFP in* yMaM330	This study
RH3919	*MRC1::mCherry::sfGFP in* yMaM330	This study
RH3920	*PUS6::mCherry::sfGFP in* yMaM330	This study
RH3921	*DNA2::mCherry::sfGFP in* yMaM330	This study
RH3922	*PRP2::mCherry::sfGFP in* yMaM330	This study
RH3923	*RPS14B::mCherry::sfGFP in* yMaM330	This study
RH3924	*APC2::mCherry::sfGFP in* yMaM330	This study
RH3925	*HBS1::mCherry::sfGFP in* yMaM330	This study
RH3466	*MATa; ura3-52; trp1D2; leu2-3_112; his3-11; ade2-1; can1-100 GAL1::SNCA::GFP::URA3 (1 copy)*	(Petroi *et al*, 2012)
RH3467	*MATa; ura3-52; trp1D2; leu2-3_112; his3-11; ade2-1; can1-100 GAL1::SNCA::GFP::URA3 (2 copies)*	(Petroi *et al*, 2012)

RPN14-GFP	*RPN14*-GFP: MATa; *his3Δ1; leu2 Δ0; met15 Δ0; ura3Δ0; rpn14Δ::RPN14::GFP::HIS3-MX6*	Yeast GFP collection, Invitrogen, USA
Tet-RPT2	*pRPT2::kanR-tet07-TATA URA3::CMV-tTA MATa his3-1 leu2-0 met15-0*	yTHC collection, Horizon Discovery, UK
Tet-RPT4	*pRPT4::kanR-tet07-TATA URA3::CMV-tTA MATa his3-1 leu2-0 met15-0*	yTHC collection, Horizon Discovery, UK
Tet-RPT6	*pRPT6::kanR-tet07-TATA URA3::CMV-tTA MATa his3-1 leu2-0 met15-0*	yTHC collection, Horizon Discovery, UK
RH3926	*pRPT2::kanR-tet07-TATA URA3::CMV-tTA MATa his3-1 leu2-0 met15-0 rpn14Δ::NatMX6*	This study
RH3927	*pRPT4::kanR-tet07-TATA URA3::CMV-tTA MATa his3-1 leu2-0 met15-0 rpn14Δ::NatMX6*	This study
RH3928	*pRPT6::kanR-tet07-TATA URA3::CMV-tTA MATa his3-1 leu2-0 met15-0 rpn14Δ::NatMX6*	This study
EGY48	MATa*, his3, trp1, ura3, LexAop (x6)-LEU2*	(Golemis *et al*, 1999)
YYS40	MATa *rpn11::RPN11-3xFLAG-HIS3*	(Saeki *et al*, 2009)
YYS1601	MATα *rpn1::RPN1-yEGFP1F-LEU2 rpn7::RPN7-2HA-mCherry-URA3*	(Saeki *et al*, 2009)
YYS1604	MATa *rpn1::RPN1-yEGFP1F-LEU2 rpn7::RPN7-2xHA-mCherry-URA3 Δrpn14::TRP1*	(Saeki *et al*, 2009)

Table S2. Plasmids used in this study.

Name	Description	Source
p423	*2μ, HIS3, GAL1^{pr}, CYC1^{term}, AmpR*	(Mumberg *et al*, 1994)
p425	*2μ, LEU2, GAL1^{pr}, CYC1^{term}, AmpR*	(Mumberg *et al*, 1994)
p426	*2μ, URA3, GAL1^{pr}, CYC1^{term}, AmpR*	(Mumberg *et al*, 1994)
p415	*CEN, LEU2, GAL1^{pr}, CYC1^{term}, AmpR*	(Mumberg *et al*, 1994)
pME3760	*p426-GAL1^{pr}::SNCA*	(Petroi *et al*, 2012)
pME3763	*p426-GAL1^{pr}::SNCA::GFP*	(Petroi *et al*, 2012)
pME5320	*p426-GAL1^{pr}::SNCA^{S129A}*	(Popova *et al*, 2021a)
pME5513	*p423-GAL1^{pr}::SNCA^{S129A}::GFP*	This study
pUG6	*loxP::pAgTEF1::kanMX::tAgTEF1::loxP*	Euroscarf
pME5514	*pUG6-ura3Δ0(200 bp)*	This study
pME5515	*pBP474-GAL1^{pr}::SNCA^{WT}::CYC1^{term}*	This study
pME5516	*pBP474-GAL1^{pr}::SNCA^{S129A}::CYC1^{term}*	This study
pME5033	*p423-GAL1^{pr}::SNCA::VenusC*	(Tenreiro *et al*, 2016)
pME5034	*p426-GAL1^{pr}::VenusN::SNCA*	(Tenreiro *et al*, 2016)
pME5039	*p425-GAL1^{pr}::SNCA::GFP*	This study
pME5090	*p426-GAL1^{pr}::VenusN-Stop*	(Popova *et al*, 2021b)
pME5517	*p423-GAL1^{pr}::SNCA^{S129A}::VenusC*	This study
pME5518	*p423-GAL1^{pr}::SNCA^{S129D}::VenusC*	This study
pME5519	*p426-GAL1^{pr}::VenusN::RPN14*	This study
pME5520	*p415-GPD^{pr}::RPN14::mCherry::sfGFP*	This study
pME5521	*p425-GPD^{pr}::RPN14::mCherry::sfGFP*	This study
pME5522	*p415-GPD^{pr}::RPN14::His6*	This study
pRS313	*CEN, HIS3, AmpR*	(Sikorski & Hieter, 1989)
pME5523	*pRS313-GPD^{pr}::CYC1^{term}*	This study
pME5524	*pRS313-GPD^{pr}::RPN14::His6::CYC1^{term}*	This study
pEG202	*2μ, HIS3, ADH^{pr}, LexA, ADH^{term}*	(Golemis *et al*, 1999)
pJG4-5	*2μ, TRP1, GAL1^{pr}, B42, ADH^{term}*	(Golemis *et al*, 1999)
pME5525	*pEG202-SNCA^{WT}*	This study
pME5526	*pJG4-5-SNCA^{WT}*	This study
pME5527	*pJG4-5-SNCA^{S129A}*	This study
pME5528	*pJG4-5-SNCA^{S129D}*	This study
pME5529	*pEG202-RPN14*	This study
pME5530	*pEG202-SNCA^{S129A}*	This study
pME5531	*pEG202-SNCA^{S129D}*	This study
ABIN5763273	*pORF-PAAF1*	antibodies-online GmbH, Germany
pME5532	*pcDNA3.1-PAAF1*	This study
pME4093	*p423-GPD^{pr}::GRK5*	(Shahpasandzadeh *et al*, 2014)

Table S3. Genes encoding proteins with significantly changed stability upon expression of αSyn in comparison to the empty vector (EV) control. Ratio represents log2 from mCherry/sfGFP fluorescence intensities. Δ-score ($RatioDiff_{(EV\text{-}\alpha Syn)}$) represents $log2(R_{EV})$-$log2(R_{\alpha Syn})$ and is a measure for changed stability. Negative Δ-score indicates stabilization of the fusion protein upon αSyn expression.

ORF	Gene	Name	Ratio (EV)	Ratio (αSyn)	Δ-score	p-value (EV-αSyn
YGR142W	*BTN2*	BaTteN disease	-5.27	0.85	-6.12	0.01
YGL004C	*RPN14*	Regulatory Particle Non-ATPase	0.52	6.01	-5.49	0.00
YDR185C	*UPS3*	UnProceSsed	-2.89	2.55	-5.44	0.00
YOR244W	*ESA1*	Catalytic subunit of the histone acetyltransferase complex	-0.73	4.46	-5.20	0.01
YDL048C	*STP4*	protein with similarity to Stp1p	-5.28	-0.11	-5.18	0.00
YPL026C	*SKS1*	Suppressor Kinase of SNF3	-3.72	1.40	-5.12	0.00
YOR344C	*TYE7*	Ty1-mediated Expression	-5.68	-0.74	-4.94	0.00
YGR238C	*KEL2*	KELch repeat	-0.32	4.49	-4.80	0.00
YDR523C	*SPS1*	SPorulation Specific	-2.14	2.62	-4.75	0.00
YDR279W	*RNH202*	RNase H	0.38	5.02	-4.64	0.00
YCL039W	*GID7*	Glucose Induced Degradation deficient	-3.16	1.46	-4.63	0.00
YDL063C	*SYO1*	SYnchronized impOrt or SYmpOrtin	0.29	4.90	-4.61	0.01
YFL007W	*BLM10*	BLeoMycin resistance	-2.63	1.96	-4.58	0.00
YEL006W	*YEA6*	Mitochondrial NAD+ transporter	-3.27	1.26	-4.54	0.01
YKR054C	*DYN1*	DYNein	-1.43	3.09	-4.52	0.00
YNL172W	*APC1*	Anaphase Promoting Complex subunit	-2.51	2.00	-4.51	0.00
YOL043C	*NTG2*	eNdonuclease Three-like Glycosylase	-0.60	3.90	-4.49	0.00
YOL116W	*MSN1*	Multicopy suppressor of SNF1 mutation	-0.71	3.70	-4.42	0.00
YCL061C	*MRC1*	Mediator of the Replication Checkpoint	-1.31	3.07	-4.38	0.00
YMR199W	*CLN1*	CycLiN	-3.97	0.38	-4.35	0.01
YGR044C	*RME1*	Regulator of MEiosis	-3.76	0.59	-4.35	0.00
YIL087C	*AIM19*	Altered Inheritance rate of Mitochondria	-3.60	0.71	-4.31	0.00
YHR061C	*GIC1*	GTPase Interactive Component	-2.67	1.61	-4.28	0.00
YML099C	*ARG81*	ARGinine requiring	-2.76	1.46	-4.22	0.01
YHR034C	*PIH1*	Protein Interacting with Hsp90	-0.38	3.84	-4.21	0.00
YMR135C	*GID8*	Glucose Induced Degradation deficient	-3.61	0.60	-4.21	0.00
YNR031C	*SSK2*	Suppressor of Sensor Kinase	0.99	5.18	-4.20	0.00
YMR056C	*AAC1*	ADP/ATP Carrier	-3.13	1.05	-4.18	0.01
YMR225C	*MRPL44*	Mitochondrial Ribosomal Protein, Large subunit	-0.99	3.15	-4.13	0.01
YMR137C	*PSO2*	PSOralen derivative sensitive	-2.22	1.91	-4.13	0.00
YBL084C	*CDC27*	Cell Division Cycle	-2.07	2.05	-4.12	0.00
YDR026C	*NSI1*	NTS1 SIlencing protein 1	-1.80	2.32	-4.12	0.00
YOR355W	*GDS1*	Involved in histone H4 acetylation	-3.94	0.16	-4.10	0.00
YLR247C	*IRC20*	Increased Recombination Centers	-1.43	2.66	-4.09	0.01
YLR385C	*SWC7*	SWr Complex	1.56	5.63	-4.07	0.00

YPL141C	*FRK1*	Fatty acyl-CoA synthetase and RNA processing-associated Kinase	-1.83	2.24	-4.07	0.00
YKL125W	*RRN3*	Regulation of RNA polymerase I	-2.73	1.31	-4.04	0.00
YIR017C	*MET28*	METhionine	-2.96	1.06	-4.02	0.00
YNL155W	*CUZ1*	Cdc48-associated UBL/Zn-finger protein	-3.71	0.29	-4.00	0.01
YMR198W	*CIK1*	Chromosome Instability and Karyogamy	-2.49	1.51	-4.00	0.00
YPL076W	*GPI2*	GlycosylPhosphatidylInositol anchor biosynthesis	-0.59	3.41	-4.00	0.01
YER013W	*PRP22*	Pre-mRNA Processing	-2.63	1.35	-3.98	0.00
YOR279C	*RFM1*	Repression Factor of Middle sporulation element	-2.73	1.24	-3.97	0.01
YLR023C	*IZH3*	Implicated in Zinc Homeostasis	-4.08	-0.11	-3.96	0.01
YNL082W	*PMS1*	PostMeiotic Segregation	-2.66	1.30	-3.95	0.00
YKL185W	*ASH1*	Asymmetric Synthesis of HO	-3.08	0.87	-3.95	0.00
YDR375C	*BCS1*	ubiquinol-cytochrome c reductase (bc1) Synthesis	-2.25	1.69	-3.94	0.00
YGL139W	*FLC3*	FLavin Carrier	-1.46	2.47	-3.92	0.01
YGL229C	*SAP4*	Sit4 Associated Protein	-2.03	1.86	-3.89	0.00
YDR118W	*APC4*	Anaphase Promoting Complex	-1.73	2.15	-3.88	0.00
YNL257C	*SIP3*	SNF1-Interacting Protein	-1.22	2.66	-3.88	0.00
YDR266C	*HEL2*	Histone E3 Ligase	-1.61	2.24	-3.85	0.00
YKL179C	*COY1*	CASP Of Yeast	-1.74	2.10	-3.84	0.00
YJL025W	*RRN7*	Regulation of RNA polymerase I	-1.18	2.61	-3.79	0.00
YML109W	*ZDS2*	Zillion Different Screens	-1.97	1.81	-3.78	0.00
YER173W	*RAD24*	RADiation sensitive	-0.75	3.01	-3.76	0.00
YOR346W	*REV1*	REVersionless	-0.39	3.37	-3.76	0.01
YLR098C	*CHA4*	Catabolism of Hydroxy Amino acids	-1.65	2.11	-3.76	0.01
YNR045W	*PET494*	PETite colonies	-1.57	2.17	-3.74	0.00
YDR052C	*DBF4*	DumbBell Former	-2.24	1.50	-3.73	0.00
YER124C	*DSE1*	Daughter Specific Expression	-4.10	-0.37	-3.73	0.00
YDR443C	*SSN2*	Suppressor of SNf1	-0.53	3.17	-3.70	0.00
YMR048W	*CSM3*	Chromosome Segregation in Meiosis	-1.27	2.42	-3.69	0.01
YKR022C	*NTR2*	NineTeen complex Related protein	-1.10	2.57	-3.66	0.00
YLR003C	*CMS1*	Complementation of Mcm-10 Suppressor	-2.83	0.82	-3.65	0.00
YIL050W	*PCL7*	Pho85 CycLin	-3.20	0.44	-3.64	0.00
YDR076W	*RAD55*	RADiation sensitive	-1.28	2.33	-3.62	0.01
YJL139C	*YUR1*	Yeast Unknown Reading frame	-1.43	2.18	-3.61	0.00
YLR105C	*SEN2*	Splicing ENdonuclease	-1.56	2.02	-3.59	0.00
YOR337W	*TEA1*	Ty Enhancer Activator	-1.71	1.86	-3.57	0.00
YKL078W	*DHR2*	DEAH-box RNA helicase	-1.26	2.29	-3.55	0.00
YGL162W	*SUT1*	Sterol UpTake	-1.10	2.44	-3.55	0.01
YLR135W	*SLX4*	Synthetic Lethal of unknown (X) function	-1.32	2.22	-3.54	0.00
YDR439W	*LRS4*	Loss of RDNA Silencing	-1.87	1.67	-3.54	0.00
YOR162C	*YRR1*	Yeast Reveromycin-A Resistant	-2.10	1.43	-3.54	0.00
YDR125C	*ECM18*	ExtraCellular Mutant	-1.32	2.21	-3.53	0.00
YBR008C	*FLR1*	FLuconazole Resistance	-1.90	1.63	-3.53	0.00

YAR031W	*PRM9*	Pheromone-Regulated Membrane protein	-0.61	2.91	-3.52	0.00
YFL004W	*VTC2*	Vacuolar Transporter Chaperone	-3.14	0.38	-3.52	0.00
YIR005W	*IST3*	Increased Sodium Tolerance	-1.73	1.77	-3.50	0.00
YBL063W	*KIP1*	KInesin related Protein	-1.13	2.36	-3.49	0.01
YNL321W	*VNX1*	Vacuolar Na+/H+ eXchanger	-1.41	2.06	-3.48	0.00
YBR042C	*CST26*	Chromosome STability	-1.08	2.39	-3.47	0.00
YPL179W	*PPQ1*	Protein Phosphatase Q	-2.27	1.19	-3.46	0.00
YKL074C	*MUD2*	Mutant U1 Die	-1.83	1.63	-3.46	0.00
YOL100W	*PKH2*	Pkb-activating Kinase Homolog	-1.18	2.28	-3.46	0.00
YJL110C	*GZF3*	Gata Zinc Finger protein	-2.43	1.02	-3.45	0.00
YJR097W	*JJJ3*	J-protein (Type III)	-1.27	2.17	-3.44	0.00
YKL043W	*PHD1*	PseudoHyphal Determinant	-1.66	1.77	-3.43	0.00
YOR373W	*NUD1*	Mitotic exit network (MEN) scaffold protein	-0.69	2.74	-3.43	0.00
YOR311C	*DGK1*	DiacylGlycerol Kinase	-0.90	2.53	-3.43	0.00
YMR224C	*MRE11*	Meiotic REcombination	-1.29	2.13	-3.42	0.00
YNL053W	*MSG5*	Multicopy Suppressor of GPA1	-2.24	1.18	-3.42	0.00
YOL105C	*WSC3*	Cell wall integrity and Stress response Component	-0.97	2.44	-3.41	0.00
YHR036W	*BRL1*	BRr6 Like protein	-1.24	2.17	-3.41	0.00
YKL101W	*HSL1*	Histone Synthetic Lethal	-1.28	2.11	-3.39	0.00
YOR025W	*HST3*	Homolog of SIR Two (SIR2)	-2.60	0.77	-3.37	0.00
YJL004C	*SYS1*	Suppressor of Ypt Six	-1.03	2.33	-3.37	0.00
YCR018C	*SRD1*	Involved in the processing of pre-rRNA	-2.48	0.88	-3.37	0.00
YKR077W	*MSA2*	Mbf and Sbf Associated	-3.19	0.17	-3.36	0.00
YPL230W	*USV1*	Up in StarVation	-2.38	0.96	-3.35	0.00
YDR184C	*ATC1*	Aip Three Complex	-1.78	1.57	-3.35	0.00
YAL041W	*CDC24*	Cell Division Cycle	-0.33	2.99	-3.32	0.00
YBR065C	*ECM2*	ExtraCellular Mutant	-0.87	2.45	-3.32	0.00
YDR247W	*VHS1*	Viable in a Hal3 Sit4 background	-2.29	1.02	-3.31	0.00
YLR193C	*UPS1*	UnProceSsed	-2.58	0.68	-3.26	0.00
YER033C	*ZRG8*	Zinc Regulated Gene	-1.73	1.52	-3.24	0.00
YKL033W	*TTI1*	Two Tel2-Interacting protein	-1.18	2.06	-3.24	0.00
YIL017C	*VID28*	Vacuolar Import and Degradation	-1.24	1.99	-3.23	0.00
YER167W	*BCK2*	Bypass of C Kinase	-1.34	1.88	-3.23	0.00
YFL029C	*CAK1*	Cdk-Activating Kinase	-1.21	2.01	-3.22	0.01
YLR183C	*TOS4*	Target Of Sbf	-2.44	0.78	-3.22	0.00
YOL077W-A	*ATP19*	ATP synthase	-3.55	-0.33	-3.21	0.00
YOR127W	*RGA1*	Rho GTPase Activating Protein	-1.16	2.05	-3.21	0.01
YDR323C	*PEP7*	carboxyPEPtidase Y-deficient	-1.17	2.03	-3.20	0.00
YML071C	*COG8*	Conserved Oligomeric Golgi complex	-1.31	1.89	-3.20	0.01
YGR206W	*MVB12*	MultiVesicular Body sorting factor of 12 kilodaltons	-1.52	1.67	-3.19	0.00
YGL222C	*EDC1*	Enhancer of mRNA DeCapping	-2.99	0.19	-3.18	0.00
YPL242C	*IQG1*	IQGAP-related protein	-1.20	1.98	-3.18	0.00

YNL230C	*ELA1*	ELongin A	-0.91	2.23	-3.13	0.00
YLR442C	*SIR3*	Silent Information Regulator	-1.20	1.93	-3.13	0.00
YJR089W	*BIR1*	Baculoviral IAP Repeat-containing protein	-1.05	2.07	-3.12	0.00
YMR119W	*ASI1*	Amino acid Sensor-Independent	-0.56	2.56	-3.12	0.00
YLR127C	*APC2*	Anaphase Promoting Complex	-1.52	1.60	-3.12	0.00
YLR401C	*DUS3*	DihydroUridine Synthase	-1.26	1.86	-3.11	0.00
YGR251W	*NOP19*	NucleOlar Protein	-0.49	2.62	-3.11	0.00
YBR083W	*TEC1*	Transposon Enhancement Control	-1.54	1.57	-3.10	0.00
YDR189W	*SLY1*	Suppressor of Loss of Ypt1	0.19	3.29	-3.10	0.00
YMR172W	*HOT1*	High-Osmolarity-induced Transcription	-1.19	1.89	-3.09	0.00
YLR034C	*SMF3*	Involved in iron homeostasis	-2.86	0.21	-3.07	0.00
YMR268C	*PRP24*	Pre-mRNA Processing	-0.63	2.44	-3.07	0.01
YPL133C	*RDS2*	Regulator of Drug Sensitivity	-1.65	1.39	-3.05	0.00
YDL070W	*BDF2*	BromoDomain Factor	-2.25	0.79	-3.04	0.00
YMR036C	*MIH1*	Mitotic Inducer Homolog	-0.34	2.69	-3.03	0.01
YAL031C	*GIP4*	Glc7 Interacting Protein	-0.71	2.30	-3.02	0.00
YFL033C	*RIM15*	Regulator of IME2	-0.50	2.52	-3.02	0.00
YGR169C	*PUS6*	PseudoUridine Synthase	-1.42	1.59	-3.01	0.00
YOR271C	*FSF1*	Fungal SideroFlexin 1	-0.35	2.66	-3.01	0.00
YGR099W	*TEL2*	TELomere maintenance	-1.12	1.89	-3.00	0.00
YJL198W	*PHO90*	PHOsphate metabolism	-1.18	1.82	-3.00	0.00
YAL056W	*GPB2*	Regulator of cAMP-PKA signaling	-0.51	2.47	-2.98	0.00
YDR464W	*SPP41*	Suppressor of PrP4	-0.90	2.08	-2.98	0.00
YNL023C	*FAP1*	FKBP12-Associated Protein	-0.50	2.47	-2.97	0.00
YGL175C	*SAE2*	Sporulation in the Absence of spo Eleven	-0.14	2.83	-2.97	0.01
YBR179C	*FZO1*	FuZzy Onions homolog	-1.77	1.18	-2.95	0.00
YIR015W	*RPR2*	RNase P Ribonucleoprotein	-1.75	1.20	-2.95	0.00
YPR134W	*MSS18*	Mitochondrial Splicing System	-0.40	2.54	-2.94	0.00
YNL063W	*MTQ1*	Methyltransferase	-0.80	2.13	-2.93	0.01
YPR031W	*NTO1*	NuA Three Orf	-1.19	1.73	-2.92	0.00
YMR232W	*FUS2*	cell FUSion	-1.22	1.70	-2.92	0.00
YPL157W	*TGS1*	TrimethylGuanosine Synthase	-1.02	1.89	-2.91	0.00
YNL133C	*FYV6*	Function required for Yeast Viability	-0.46	2.45	-2.91	0.00
YPR140W	*TAZ1*	TAfaZzin	-0.75	2.16	-2.91	0.00
YGL065C	*ALG2*	Asparagine-Linked Glycosylation	0.22	3.13	-2.91	0.00
YIL061C	*SNP1*	Component of U1 snRNP	-1.45	1.45	-2.91	0.00
YBL102W	*SFT2*	Suppressor of sed Five Ts	0.31	3.21	-2.90	0.00
YGL240W	*DOC1*	Destruction Of Cyclin B	-0.62	2.28	-2.89	0.00
YBR168W	*PEX32*	PEroXisome related	-0.73	2.15	-2.89	0.00
YNR011C	*PRP2*	Pre-mRNA Processing	-1.55	1.32	-2.87	0.00
YKL146W	*AVT3*	Amino acid Vacuolar Transport	-1.57	1.30	-2.87	0.00
YLR371W	*ROM2*	RhO1 Multicopy suppressor	-1.11	1.75	-2.86	0.01
YNL129W	*NRK1*	Nicotinamide Riboside Kinase	-1.36	1.50	-2.86	0.00

YKR084C	*HBS1*	Hsp70 subfamily B Suppressor	-0.92	1.94	-2.86	0.00
YPL022W	*RAD1*	RADiation sensitive	-0.49	2.37	-2.86	0.00
YOR315W	*SFG1*	SuperFicial pseudohyphal Growth	-2.10	0.76	-2.86	0.00
YDL111C	*RRP42*	Ribosomal RNA Processing	0.86	3.70	-2.84	0.00
YBR257W	*POP4*	Processing Of Precursor RNAs	-0.93	1.91	-2.84	0.00
YDR448W	*ADA2*	transcriptional ADAptor	-1.44	1.39	-2.83	0.01
YMR288W	*HSH155*	Human Sap Homolog	-0.51	2.33	-2.83	0.00
YGR134W	*CAF130*	CCR4 Associated Factor	-1.04	1.79	-2.83	0.01
YNL027W	*CRZ1*	Calcineurin-Responsive Zinc finger	-0.90	1.92	-2.83	0.00
YLR068W	*FYV7*	Function required for Yeast Viability	-0.66	2.16	-2.82	0.01
YDR252W	*BTT1*	BTf Three	-1.30	1.51	-2.81	0.00
YCR082W	*AHC2*	Ada Histone acetyltransferase complex Component	-2.39	0.42	-2.81	0.00
YAR003W	*SWD1*	Set1c, WD40 repeat protein	0.24	3.05	-2.81	0.01
YLR107W	*REX3*	Rna EXonuclease	-1.03	1.77	-2.80	0.00
YJL056C	*ZAP1*	Zinc-responsive Activator Protein	-1.88	0.92	-2.80	0.00
YER142C	*MAG1*	3-MethylAdenine DNA Glycosylase	-0.48	2.31	-2.79	0.00
YOL028C	*YAP7*	Yeast AP-1	-1.43	1.36	-2.79	0.00
YBR095C	*RXT2*	Component of histone deacetylase Rpd3L complex	-0.46	2.32	-2.78	0.00
YPL267W	*ACM1*	APC/C[Cdh1] Modulator	-1.99	0.78	-2.77	0.00
YNL273W	*TOF1*	TOpoisomerase I-interacting Factor	-0.55	2.22	-2.77	0.00
YLR215C	*CDC123*	Cell Division Cycle	-0.10	2.67	-2.76	0.00
YNL068C	*FKH2*	ForK head Homolog	-1.23	1.53	-2.76	0.00
YOL113W	*SKM1*	STE20/PAK homologous Kinase related to Morphogenesis	-0.85	1.91	-2.76	0.00
YML076C	*WAR1*	Weak Acid Resistance	-0.79	1.97	-2.76	0.00
YPL256C	*CLN2*	CycLiN	-2.34	0.40	-2.75	0.00
YGR096W	*TPC1*	Thiamine Pyrophosphate Carrier	-0.50	2.25	-2.74	0.01
YNL298W	*CLA4*	CLn Activity dependant	-0.28	2.45	-2.73	0.00
YDR410C	*STE14*	STErile	0.00	2.73	-2.73	0.00
YOL072W	*THP1*	Tho2/Hpr1 Phenotype	-0.35	2.38	-2.73	0.01
YNR023W	*SNF12*	Sucrose NonFermenting	-0.79	1.94	-2.73	0.00
YNL213C	*RRG9*	Required for Respiratory Growth	-0.78	1.95	-2.73	0.00
YPL155C	*KIP2*	KInesin related Protein	-0.97	1.75	-2.73	0.00
YEL059C-A	*SOM1*	SOrting Mitochondrial	-0.31	2.42	-2.72	0.00
YNR055C	*HOL1*	HistidinOl	-0.42	2.30	-2.72	0.00
YDR291W	*HRQ1*	Homologous to RecQ protein	-0.64	2.08	-2.72	0.00
YDL087C	*LUC7*	Lethal Unless Cap-binding complex is produced	-1.37	1.34	-2.72	0.00
YHR164C	*DNA2*	DNA synthesis defective	-0.04	2.67	-2.71	0.01
YPL115C	*BEM3*	Bud EMergence	-0.82	1.87	-2.70	0.00
YDR219C	*MFB1*	Mitochondria-associated F-Box protein	-1.62	1.08	-2.70	0.00
YBR195C	*MSI1*	Multicopy Suppressor of IRA1	-0.83	1.86	-2.69	0.00
YDR376W	*ARH1*	Adrenodoxin Reductase Homolog	-0.20	2.48	-2.68	0.00
YBR097W	*VPS15*	Vacuolar Protein Sorting	-0.85	1.83	-2.68	0.00

YHR090C	*YNG2*	Yeast iNG1 homolog	-0.86	1.82	-2.68	0.00
YLR265C	*NEJ1*	Nonhomologous End-Joining defective	-0.82	1.84	-2.67	0.00
YMR127C	*SAS2*	Something About Silencing	0.67	3.34	-2.66	0.00
YCR092C	*MSH3*	MutS Homolog	-0.53	2.13	-2.66	0.00
YMR138W	*CIN4*	Chromosome INstability	-1.01	1.65	-2.66	0.00
YJL191W	*RPS14B*	Ribosomal Protein of the Small subunit	-1.97	0.69	-2.66	0.00
YGL113W	*SLD3*	Synthetically Lethal with Dpb11-1	-0.17	2.49	-2.66	0.00
YLR451W	*LEU3*	LEUcine biosynthesis	-1.58	1.07	-2.65	0.00
YER107C	*GLE2*	GLFG LEthal	-1.29	1.35	-2.64	0.01
YIL009W	*FAA3*	Fatty Acid Activation	-0.62	2.00	-2.62	0.00
YER075C	*PTP3*	Protein Tyrosine Phosphatase	-0.85	1.78	-2.62	0.00
YCR024C	*SLM5*	Synthetic Lethal with Mss4	-0.10	2.52	-2.62	0.00
YCL055W	*KAR4*	KARyogamy	-0.68	1.94	-2.62	0.00
YLR115W	*CFT2*	Cleavage Factor Two	-0.51	2.10	-2.61	0.00
YDR437W	*GPI19*	Glycosyl PhosphatidylInositol anchor biosynthesis	-1.03	1.58	-2.61	0.00
YPL140C	*MKK2*	Mitogen-activated Kinase Kinase	-1.55	1.06	-2.61	0.00
YHR171W	*ATG7*	AuTophaGy related	-0.40	2.21	-2.60	0.01
YGR171C	*MSM1*	Mitochondrial aminoacyl-tRNA Synthetase, Methionine	-0.26	2.34	-2.60	0.00
YDL080C	*THI3*	THIamine metabolism	-0.58	2.02	-2.59	0.01
YKL114C	*APN1*	APurinic/apyrimidinic eNdonuclease	-0.94	1.65	-2.59	0.00
YMR292W	*GOT1*	GOlgi Transport	0.03	2.62	-2.59	0.00
YKL004W	*AUR1*	AUreobasidin A Resistance	-0.70	1.89	-2.59	0.00
YER132C	*PMD1*	Paralog of MDS3	-0.58	2.00	-2.59	0.00
YHR102W	*KIC1*	Kinase that Interacts with Cdc31p	-0.90	1.69	-2.59	0.01
YJL091C	*GWT1*	GPI-anchored Wall protein Transfer	0.02	2.61	-2.59	0.00
YKR031C	*SPO14*	SPOrulation	0.13	2.71	-2.58	0.01
YDR440W	*DOT1*	Disruptor Of Telomeric silencing	0.07	2.64	-2.58	0.00
YBL060W	*YEL1*	Yeast EFA6-Like	-0.85	1.72	-2.57	0.00
YDL036C	*PUS9*	PseudoUridine Synthase	-0.51	2.06	-2.57	0.00
YMR285C	*NGL2*	Involved in 5.8S rRNA processing	-1.49	1.07	-2.57	0.00
YBR239C	*ERT1*	Ethanol Regulated Transcription factor	-0.93	1.63	-2.56	0.00
YLR452C	*SST2*	SuperSensiTive	-0.41	2.15	-2.56	0.00
YHR031C	*RRM3*	rDNA Recombination Mutation	-0.93	1.63	-2.56	0.00
YDR473C	*PRP3*	Pre-mRNA Processing	-1.03	1.52	-2.55	0.00
YER054C	*GIP2*	Glc7-Interacting Protein	-1.27	1.27	-2.54	0.00
YLR136C	*TIS11*	similar to the mammalian TPA Induced Sequence gene family	-2.36	0.18	-2.54	0.00
YNL073W	*MSK1*	Mitochondrial aminoacyl-tRNA Synthetase, lysine (K)	0.63	3.16	-2.53	0.00
YMR201C	*RAD14*	RADiation sensitive	-1.00	1.53	-2.53	0.00
YDR206W	*EBS1*	Est1-like Bcy1 Suppressor	-0.91	1.61	-2.52	0.00
YJL098W	*SAP185*	Sit4 Associated Protein	-1.13	1.40	-2.52	0.00
YGR102C	*GTF1*	Glutaminyl Transamidase subunit F	-0.69	1.83	-2.52	0.00
YMR280C	*CAT8*	CATabolite repression	-0.95	1.56	-2.52	0.00

YMR060C	*SAM37*	Sorting and Assembly Machinery	-0.15	2.36	-2.51	0.00
YER088C	*DOT6*	Disruptor Of Telomeric silencing	-1.57	0.94	-2.51	0.00
YLR382C	*NAM2*	Nuclear Accommodation of Mitochondria	-0.38	2.13	-2.50	0.00
YKL062W	*MSN4*	Multicopy suppressor of SNF1 mutation	-1.99	0.51	-2.50	0.00
YGR002C	*SWC4*	SWr Complex	-1.42	1.08	-2.50	0.00
YDR004W	*RAD57*	RADiation sensitive	-0.23	2.27	-2.50	0.00
YGR062C	*COX18*	Cytochrome c OXidase	-1.08	1.40	-2.48	0.00
YLR228C	*ECM22*	ExtraCellular Mutant	-1.14	1.34	-2.48	0.01
YNL242W	*ATG2*	AuTophaGy related	-0.37	2.11	-2.48	0.00
YDR423C	*CAD1*	CADmium resistance	-1.13	1.35	-2.47	0.00
YLR085C	*ARP6*	Actin-Related Protein	-0.56	1.91	-2.47	0.01
YMR211W	*DML1*	Drosophila melanogaster Misato-Like protein	-0.27	2.20	-2.47	0.01
YER147C	*SCC4*	Sister Chromatid Cohesion	-0.59	1.88	-2.46	0.00
YDR495C	*VPS3*	Vacuolar Protein Sorting	-0.21	2.25	-2.46	0.00
YOL080C	*REX4*	Rna EXonuclease	0.22	2.67	-2.46	0.00
YNL233W	*BNI4*	Bud Neck Involved	-0.83	1.62	-2.45	0.00
YNR004W	*SWM2*	Synthetic With mud2-delta	-0.53	1.92	-2.45	0.00
YJR047C	*ANB1*	ANaeroBically induced	-0.43	2.02	-2.44	0.01
YML111W	*BUL2*	Binds Ubiquitin Ligase	-0.18	2.26	-2.44	0.00
YJR055W	*HIT1*	HIgh Temperature growth	-0.44	1.99	-2.43	0.00
YDL030W	*PRP9*	Pre-mRNA Processing	-0.62	1.81	-2.42	0.00
YLR272C	*YCS4*	Yeast Condensin Subunit	-1.08	1.35	-2.42	0.00
YGL059W	*PKP2*	Protein Kinase of PDH	-1.07	1.35	-2.42	0.00
YLR077W	*FMP25*	Found in Mitochondrial Proteome	-0.02	2.40	-2.41	0.00
YKR049C	*FMP46*	Found in Mitochondrial Proteome	-0.55	1.86	-2.41	0.01
YCR033W	*SNT1*	SaNT domains	-0.64	1.77	-2.41	0.00
YBR193C	*MED8*	MEDiator complex	-0.59	1.81	-2.41	0.01
YML059C	*NTE1*	Neuropathy Target Esterase	0.04	2.44	-2.40	0.00
YKL020C	*SPT23*	SuPpressor of Ty	-0.84	1.57	-2.40	0.00
YER162C	*RAD4*	RADiation sensitive	-0.16	2.23	-2.39	0.00
YGL226C-A	*OST5*	OligoSaccharylTransferase	0.39	2.77	-2.38	0.00
YDL001W	*RMD1*	Required for Meiotic nuclear Division	-0.90	1.48	-2.38	0.00
YGL166W	*CUP2*	Copper-binding transcription factor	-0.87	1.50	-2.37	0.00
YDR288W	*NSE3*	Non SMC Element	-0.45	1.92	-2.37	0.00
YOL056W	*GPM3*	Glycerate PhosphoMutase	-1.52	0.84	-2.36	0.00
YPL138C	*SPP1*	Set1c, Phd finger Protein	-0.67	1.69	-2.36	0.00
YLR188W	*MDL1*	MultiDrug resistance-Like	-0.99	1.37	-2.36	0.01
YGL085W	*LCL3*	Long Chronological Lifespan 3	-0.65	1.70	-2.36	0.01
YOR228C	*MCP1*	Mdm10 Complementing Protein	-0.79	1.56	-2.35	0.00
YPL072W	*UBP16*	UBiquitin-specific Protease	0.11	2.46	-2.35	0.01
YHR041C	*SRB2*	Suppressor of RNA polymerase B	-0.41	1.93	-2.34	0.00
YHR116W	*COX23*	Cytochrome OXidase	1.17	3.51	-2.34	0.00
YOR249C	*APC5*	Anaphase Promoting Complex	-1.23	1.11	-2.34	0.00

YLR051C	*FCF2*	Faf1p Copurifying Factor	-0.64	1.69	-2.33	0.01
YLR086W	*SMC4*	Structural Maintenance of Chromosomes	-0.85	1.48	-2.33	0.00
YIL079C	*AIR1*	Arginine methyltransferase-Interacting RING finger protein	-0.54	1.79	-2.33	0.00
YNL078W	*NIS1*	Neck protein Interacting with Septins	-0.65	1.68	-2.33	0.00
YBR055C	*PRP6*	Pre-mRNA Processing	-0.83	1.50	-2.32	0.00
YIL056W	*VHR1*	VHt1 Regulator	-1.10	1.23	-2.32	0.00
YFR048W	*RMD8*	Required for Meiotic nuclear Division	0.56	2.89	-2.32	0.00
YPL177C	*CUP9*	Transcriptional repressor	-2.17	0.14	-2.31	0.00
YLR130C	*ZRT2*	Zinc-Regulated Transporter	-0.85	1.46	-2.31	0.00
YER111C	*SWI4*	SWItching deficient	-0.82	1.50	-2.31	0.00
YJL203W	*PRP21*	Pre-mRNA Processing	-0.72	1.59	-2.31	0.00
YHL025W	*SNF6*	Sucrose NonFermenting	-0.08	2.23	-2.31	0.00
YPR147C	*YPR147C*	Bifunctional triacylglycerol lipase	-1.72	0.34	-2.06	0.01
YBR291C	*CTP1*	Citrate Transport Protein	-1.81	0.07	-1.88	0.00
YOL013C	*HRD1*	HMG-coA Reductase Degradation	-0.30	1.56	-1.86	0.00
YPR049C	*ATG11*	AuTophaGy related	0.00	1.62	-1.62	0.00
YNL185C	*MRPL19*	Mitochondrial Ribosomal Protein, Large subunit	-0.52	1.01	-1.53	0.00
YPR174C	*CSA1*	Cdc5 SPB Anchor	0.04	1.52	-1.48	0.00
YGR084C	*MRP13*	Mitochondrial Ribosomal Protein	-0.02	1.37	-1.39	0.00
YIL107C	*PFK26*	6-PhosphoFructo-2-Kinase	0.04	1.35	-1.31	0.00
YHR001W-A	*QCR10*	ubiQuinol-cytochrome C oxidoReductase	-2.93	-1.64	-1.29	0.00
YMR098C	*ATP25*	ATPase	0.28	1.57	-1.29	0.00
YPR114W	*YPR114W*	Similar to ceramide synthases	1.93	3.22	-1.28	0.00
YLL008W	*DRS1*	Deficiency of Ribosomal Subunits	0.41	1.60	-1.18	0.00
YMR208W	*ERG12*	ERGosterol biosynthesis	-0.82	0.34	-1.17	0.00
YOL082W	*ATG19*	AuTophaGy related	0.09	1.07	-0.98	0.00
YER035W	*EDC2*	Enhancer of mRNA DeCapping	-1.51	-0.54	-0.97	0.00
YDL222C	*FMP45*	Found in Mitochondrial Proteome	0.46	1.41	-0.95	0.00
YDL064W	*UBC9*	UBiquitin-Conjugating	0.11	0.95	-0.84	0.00
YCR021C	*HSP30*	Heat Shock Protein	0.60	1.43	-0.83	0.00
YNL137C	*NAM9*	Nuclear Accommodation of Mitochondria	-0.12	0.67	-0.79	0.01
YIL111W	*COX5B*	Cytochrome c OXidase	-0.28	0.45	-0.73	0.00
YDR342C	*HXT7*	HeXose Transporter	1.05	1.76	-0.70	0.00
YDR343C	*HXT6*	HeXose Transporter	0.42	1.07	-0.65	0.00
YLR290C	*COQ11*	COenzyme Q	0.19	0.81	-0.62	0.00
YER103W	*SSA4*	Stress-Seventy subfamily A	-0.17	0.45	-0.61	0.00
YML081C-A	*ATP18*	ATP synthase	-0.63	-0.07	-0.57	0.00
YBR169C	*SSE2*	Stress Seventy subfamily E	0.11	0.58	-0.46	0.00
YNL052W	*COX5A*	Cytochrome c OXidase	0.20	0.66	-0.46	0.00
YPL061W	*ALD6*	ALdehyde Dehydrogenase	-0.11	0.34	-0.46	0.00
YPL004C	*LSP1*	Long chain bases Stimulate Phosphorylation	0.31	0.76	-0.45	0.00
YFL014W	*HSP12*	Heat Shock Protein	0.29	0.72	-0.43	0.00

YML128C	*MSC1*	Meiotic Sister-Chromatid recombination	0.56	0.99	-0.43	0.00
YDR432W	*NPL3*	Nuclear Protein Localization	0.95	1.37	-0.42	0.00
YBR072W	*HSP26*	Heat Shock Protein	1.07	1.49	-0.42	0.00
YNL055C	*POR1*	PORin	-3.14	-2.73	-0.41	0.00
YMR175W	*SIP18*	Salt Induced Protein	0.32	0.71	-0.40	0.00
YDL223C	*HBT1*	HuB1 Target	0.41	0.80	-0.39	0.00
YDR225W	*HTA1*	Histone h Two A	0.12	0.51	-0.39	0.00
YGL245W	*GUS1*	GlUtamyl-tRNA Synthetase	-0.09	0.30	-0.38	0.00
YPR069C	*SPE3*	SPErmidine auxotroph	0.41	0.78	-0.38	0.01
YDR033W	*MRH1*	Membrane protein Related to Hsp30p	0.08	0.46	-0.37	0.00
YNL016W	*PUB1*	PolyUridylate Binding	-0.10	0.27	-0.37	0.00
YNR018W	*RCF2*	Respiratory superComplex Factor	0.31	0.69	-0.37	0.00
YMR092C	*AIP1*	Actin Interacting Protein	0.01	0.38	-0.37	0.00
YHR193C	*EGD2*	Enhancer of Gal4 DNA binding	-0.01	0.35	-0.37	0.00
YKL035W	*UGP1*	UDP-glucose pyrophosphorylase	0.14	0.51	-0.37	0.00
YBL003C	*HTA2*	Histone h Two A	0.01	0.37	-0.36	0.00
YIL053W	*GPP1*	Glycerol-3-Phosphate Phosphatase	0.05	0.40	-0.36	0.00
YNR016C	*ACC1*	Acetyl-CoA Carboxylase	0.14	0.50	-0.35	0.01
YOR187W	*TUF1*	Mitochondrial translation elongation factor Tu (EF-Tu)	0.26	0.60	-0.34	0.00
YJL088W	*ARG3*	ARGinine requiring	0.41	0.75	-0.34	0.00
YLR180W	*SAM1*	S-AdenosylMethionine requiring	0.16	0.49	-0.33	0.00
YFR053C	*HXK1*	HeXoKinase	0.05	0.39	-0.33	0.00
YOR232W	*MGE1*	Mitochondrial GrpE	0.09	0.42	-0.33	0.00
YBR011C	*IPP1*	Inorganic PyroPhosphatase	-0.11	0.22	-0.33	0.00
YGL008C	*PMA1*	Plasma Membrane ATPase	0.31	0.64	-0.33	0.00
YDR012W	*RPL4B*	Ribosomal Protein of the Large subunit	0.05	0.38	-0.33	0.00
YGL105W	*ARC1*	Aminoacyl-tRNA synthetase Cofactor	-0.04	0.27	-0.31	0.00
YHR087W	*RTC3*	Restriction of Telomere Capping	0.43	0.73	-0.31	0.01
YNL096C	*RPS7B*	Ribosomal Protein of the Small subunit	0.00	0.30	-0.31	0.00
YBL002W	*HTB2*	Histone h Two B	0.12	0.42	-0.31	0.00
YGL256W	*ADH4*	Alcohol DeHydrogenase	0.72	1.03	-0.30	0.00
YBR088C	*POL30*	POLymerase	0.72	0.49	0.22	0.01
YJR121W	*ATP2*	ATP synthase	1.11	0.75	0.36	0.01
YLR304C	*ACO1*	ACOnitase	1.31	0.53	0.78	0.01
YOR062C	*YOR062C*	Similar to Reg1	0.37	-0.65	1.02	0.04
YLR355C	*ILV5*	IsoLeucine-plus-Valine requiring	4.63	3.58	1.04	0.01

Table S4. Genes encoding proteins with significantly changed stability upon expression of S129A in comparison to the empty vector (EV) control. Ratio represents log2 from mCherry/sfGFP fluorescence intensities. Δ-score ($RatioDiff_{(EV-S129A)}$) represents $log2(R_{EV})$-$log2(R_{S129A})$ and is a measure for changed stability. Negative Δ-score indicates stabilization of the fusion protein upon S129A expression.

ORF	Gene	Name	Ratio (EV)	Ratio (S129A)	Δ-score	p-value (EV- S129A)
YDR185C	*UPS3*	UnProceSsed	-2.89	2.27	-5.16	0.01
YGL139W	*FLC3*	FLavin Carrier	-1.46	3.58	-5.03	0.01
YMR066W	*SOV1*	Synthesis Of Var	-0.73	4.00	-4.73	0.01
YDR026C	*NSI1*	NTS1 SIlencing protein 1	-1.80	2.62	-4.42	0.01
YMR198W	*CIK1*	Chromosome Instability and Karyogamy	-2.49	1.63	-4.12	0.01
YMR048W	*CSM3*	Chromosome Segregation in Meiosis	-1.27	2.81	-4.08	0.01
YLR247C	*IRC20*	Increased Recombination Centers	-1.43	2.47	-3.90	0.01
YGL162W	*SUT1*	Sterol UpTake	-1.10	2.55	-3.65	0.01
YNL230C	*ELA1*	ELongin A	-0.91	2.74	-3.64	0.01
YGR134W	*CAF130*	CCR4 Associated Factor	-1.04	2.54	-3.57	0.01
YPL005W	*AEP3*	ATPase ExPression	0.15	3.72	-3.57	0.01
YPL139C	*UME1*	Unscheduled Meiotic gene Expression	-0.58	2.96	-3.54	0.01
YER173W	*RAD24*	RADiation sensitive	-0.75	2.76	-3.51	0.01
YGR200C	*ELP2*	ELongator Protein	0.83	4.27	-3.45	0.01
YIL098C	*FMC1*	Formation of Mitochondrial Complexes	-0.14	3.25	-3.39	0.01
YDR076W	*RAD55*	RADiation sensitive	-1.28	2.07	-3.35	0.01
YDR118W	*APC4*	Anaphase Promoting Complex	-1.73	1.58	-3.31	0.01
YER013W	*PRP22*	Pre-mRNA Processing	-2.63	0.66	-3.29	0.01
YBR257W	*POP4*	Processing Of Precursor RNAs	-0.93	2.27	-3.20	0.01
YNL023C	*FAP1*	FKBP12-Associated Protein	-0.50	2.69	-3.19	0.01
YDR125C	*ECM18*	ExtraCellular Mutant	-1.32	1.83	-3.15	0.01
YBR008C	*FLR1*	FLuconazole Resistance	-1.90	1.21	-3.11	0.01
YKL033W	*TTI1*	Two Tel2-Interacting protein	-1.18	1.92	-3.10	0.01
YMR223W	*UBP8*	UBiquitin-specific processing Protease	0.66	3.64	-2.98	0.01
YIR005W	*IST3*	Increased Sodium Tolerance	-1.73	1.20	-2.93	0.01
YLR371W	*ROM2*	RhO1 Multicopy suppressor	-1.11	1.79	-2.89	0.01
YHR164C	*DNA2*	DNA synthesis defective	-0.04	2.85	-2.89	0.01
YGR206W	*MVB12*	MultiVesicular Body sorting factor of 12 kilodaltons	-1.52	1.35	-2.87	0.01
YKL125W	*RRN3*	Regulation of RNA polymerase I	-2.73	0.09	-2.82	0.01
YNL321W	*VNX1*	Vacuolar Na+/H+ eXchanger	-1.41	1.40	-2.81	0.01
YMR127C	*SAS2*	Something About Silencing	0.67	3.47	-2.79	0.01
YFL007W	*BLM10*	BLeoMycin resistance	-2.63	0.15	-2.77	0.01
YGR251W	*NOP19*	NucleOlar Protein	-0.49	2.25	-2.74	0.01

YBR065C	*ECM2*	ExtraCellular Mutant	-0.87	1.84	-2.72	0.01
YNL082W	*PMS1*	PostMeiotic Segregation	-2.66	0.04	-2.70	0.01
YGL240W	*DOC1*	Destruction Of Cyclin B	-0.62	2.07	-2.69	0.01
YCL039W	*GID7*	Glucose Induced Degradation deficient	-3.16	-0.50	-2.67	0.01
YKR093W	*PTR2*	Peptide TRansport	0.49	3.13	-2.65	0.01
YGR044C	*RME1*	Regulator of MEiosis	-3.76	-1.11	-2.64	0.01
YBL084C	*CDC27*	Cell Division Cycle	-2.07	0.54	-2.61	0.01
YKR084C	*HBS1*	Hsp70 subfamily B Suppressor	-0.92	1.67	-2.59	0.01
YML076C	*WAR1*	Weak Acid Resistance	-0.79	1.76	-2.55	0.01
YPR134W	*MSS18*	Mitochondrial Splicing System	-0.40	2.14	-2.54	0.01
YKL078W	*DHR2*	DEAH-box RNA helicase	-1.26	1.25	-2.51	0.01
YHR102W	*KIC1*	Kinase that Interacts with Cdc31p	-0.90	1.58	-2.48	0.01
YKL185W	*ASH1*	Asymmetric Synthesis of HO	-3.08	-0.61	-2.47	0.01
YER147C	*SCC4*	Sister Chromatid Cohesion	-0.59	1.86	-2.44	0.01
YMR232W	*FUS2*	cell FUSion	-1.22	1.21	-2.43	0.01
YLR115W	*CFT2*	Cleavage Factor Two	-0.51	1.90	-2.42	0.01
YBL018C	*POP8*	Processing Of Precursor RNAs	-0.09	2.30	-2.39	0.01
YAR014C	*BUD14*	BUD site selection	-0.49	1.89	-2.38	0.01
YEL059C-A	*SOM1*	SOrting Mitochondrial	-0.31	2.02	-2.33	0.01
YLR127C	*APC2*	Anaphase Promoting Complex	-1.52	0.78	-2.30	0.01
YLR215C	*CDC123*	Cell Division Cycle	-0.10	2.08	-2.18	0.01
YMR098C	*ATP25*	ATPase	0.28	1.16	-0.88	0.01
YLL008W	*DRS1*	Deficiency of Ribosomal Subunits	0.41	1.18	-0.76	0.01
YMR208W	*ERG12*	ERGosterol biosynthesis	-0.82	-0.35	-0.47	0.01
YJR121W	*ATP2*	ATP synthase	1.11	0.54	0.57	0.01
YBR146W	*MRPS9*	Mitochondrial Ribosomal Protein, Small subunit	-0.65	-3.18	2.53	0.01

Table S5. Spatial analysis of functional enrichment (SAFE) upon expression of aSyn or S129A.

Term	p-value EV to aSyn	fold change	Fraction of input gene list annotated to a bioprocess cluster	Cluster frequency	Background frequency
DNA replication & repair	1.26432E-07	1.952191235	25 / 333, 7.5%	7 / 25, 28.0%	251 / 1750, 14.3%
Mitosis	9.847E-06	2.886597938	25 / 333, 7.5%	4 / 25, 16.0%	97 / 1750, 5.5%
mRNA processing	0.000816384	1.721311475	25 / 333, 7.5%	3 / 25, 12.0%	122 / 1750, 7.0%
Nuclear transport	0.001145425	3.043478261	25 / 333, 7.5%	2 / 25, 8.0%	46 / 1750, 2.6%
Transcription	0.185629293	0.454545455	25 / 333, 7.5%	1 / 25, 4.0%	154 / 1750, 8.8%
Mitochondria	0.185629293	0.33492823	25 / 333, 7.5%	1 / 25, 4.0%	209 / 1750, 11.9%
**Term**	**p-value EV to S129A**	**fold change**	**Fraction of input gene list annotated to a bioprocess cluster**	**Cluster frequency**	**Background frequency**
DNA replication & repair	3.25E-06	2.988047809	7 / 56, 12.5%	3 / 7, 42.9%	251 / 1750, 14.3%
Glycosylation & Protein folding	0.011918	1.262626263	7 / 56, 12.5%	1 / 7, 14.3%	198 / 1750, 11.3%

SAFE enrichment scores:

ORF	Name	Annotations	EV-aSyn	EV-S129A
YDR279W	RNH202	DNA replication & repair	0.272987	0
YDR255C	RMD5		0.272987	0
YLR007W	NSE1	DNA replication & repair	0.264111	0
YDL105W	NSE4	DNA replication & repair	0.264111	0
YDR363W	ESC2	DNA replication & repair	0.24764	0
YNL216W	RAP1		0.228824	0
YDR399W	HPT1		0.211277	0.116169
YHR036W	BRL1	Mitosis	0.206273	0
YDL064W	UBC9	DNA replication & repair	0.199478	0
YDR311W	TFB1		0.199478	0
YAL027W	SAW1		0.199478	0
YBR088C	POL30		0.199478	0
YDL105W	NSE4	DNA replication & repair	0.199478	0
YPR119W	CLB2	DNA replication & repair	0.174776	0.104486
YKL022C	CDC16	DNA replication & repair	0.174776	0.104486
YMR001C-A		DNA replication & repair	0.174776	0.104486
YHR036W	BRL1	Nuclear transport	0.173339	0
YLR293C	GSP1	Nuclear transport	0.173339	0
YMR201C	RAD14		0.167213	0
YML023C	NSE5	DNA replication & repair	0.167127	0
YLR116W	MSL5	mRNA processing	0.166182	0
YDR288W	NSE3	DNA replication & repair	0.161223	0
YOR342C		DNA replication & repair	0.161223	0
YER107C	GLE2	Nuclear transport	0.155604	0
YGR098C	ESP1	Mitosis	0.155402	0
YBR088C	POL30	Mitosis	0.153498	0
YOL126C	MDH2	mRNA processing	0.147561	0
YEL019C	MMS21	DNA replication & repair	0.147254	0

YLR298C	YHC1	mRNA processing	0.143908	0
YLR154C	RNH203	DNA replication & repair	0.141366	0
YMR112C	MED11	Transcription	0.141366	0
YIL016W	SNL1		0.140168	0.10997
YDR045C	RPC11	mRNA processing	0.136918	0
YCL057C-A	MIC10		0.133401	0
YML023C	NSE5	DNA replication & repair	0.130539	0
YLR186W	EMG1		0.129495	0
YDR177W	UBC1	DNA replication & repair	0.127112	0.104486
YJR022W	LSM8	mRNA processing	0.125784	0
YLR201C	COQ9	Mitochondria	0.125784	0.115435
YER038C	KRE29	DNA replication & repair	0.125548	0
YMR298W	LIP1		0.125548	0
YDL033C	SLM3		0.125548	0
YOR116C	RPO31	mRNA processing	0.124512	0
YHR062C	RPP1		0.121248	0
YHR166C	CDC23	DNA replication & repair	0.121248	0.174456
YFR027W	ECO1	DNA replication & repair	0.121248	0.174456
YLR146C	SPE4		0.121248	0
YLR135W	SLX4	DNA replication & repair	0.120808	0
YML103C	NUP188	Nuclear transport	0.120808	0
YMR306W	FKS3		0.120808	0
YLR396C	VPS33	MVB Sorting & RIM Signalling	0.117046	0
YDL008W	APC11	DNA replication & repair	0.115763	0
YLR347C	KAP95	Nuclear transport	0.115763	0
YNL172W	APC1	DNA replication & repair	0.115763	0
YMR306C-A			0.115763	0
YGL116W	CDC20	DNA replication & repair	0.115763	0
YDR288W	NSE3	DNA replication & repair	0.115763	0
YGL116W	CDC20	DNA replication & repair	0.115763	0
YDR443C	SSN2	Transcription	0.115763	0
YPL042C	SSN3	Transcription	0.115763	0
YPR056W	TFB4		0.115763	0
YOR249C	APC5	Mitosis	0.115763	0
YIL091C	UTP25		0.115763	0
YJL025W	RRN7		0.115763	0
YBR237W	PRP5	mRNA processing	0.11538	0
YLR117C	CLF1	mRNA processing	0.113324	0
YHR074W	QNS1		0.112137	0
YGR129W	SYF2	mRNA processing	0.112137	0
YBR119W	MUD1	mRNA processing	0.11201	0
YPR180W	AOS1	DNA replication & repair	0.11201	0
YBR003W	COQ1	Mitochondria	0.11201	0
YDL087C	LUC7	mRNA processing	0.11132	0
YNR045W	PET494	Mitochondria	0.11062	0
YDR458C	HEH2		0.11062	0

YOR271C	FSF1		0.11062	0
YDL217C	TIM22		0.11062	0
YBL084C	CDC27	DNA replication & repair	0.11062	0
YGL008C	PMA1		0.11062	0
YBL074C	AAR2	mRNA processing	0.108998	0
YPR180W	AOS1		0.107919	0
YDR235W	PRP42	mRNA processing	0.107919	0
YBR055C	PRP6	mRNA processing	0.10781	0
YBL084C	CDC27	Mitosis	0.105787	0
YGL066W	SGF73		0.105787	0
YHR077C	NMD2		0.105787	0
YNR004W	SWM2	mRNA processing	0.104015	0
YOR224C	RPB8	mRNA processing	0.104015	0
YLR383W	SMC6	DNA replication & repair	0.101234	0
YBR088C	POL30		0.101234	0
YPL122C	TFB2		0.101234	0
YLR221C	RSA3		0.101234	0
YAL041W	CDC24		0.100743	0
YJR067C	YAE1		0.100743	0
YDR050C	TPI1		0.100285	0
YML041C	VPS71	Transcription	0.100285	0
YJR045C	SSC1		0	0.107148
YBR283C	SSH1		0	0.123282
YPR080W	TEF1		0	0.107148
YHR191C	CTF8	DNA replication & repair	0	0.109214
YGR092W	DBF2	DNA replication & repair	0	0.207079
YDR310C	SUM1	Glycosylation & Protein folding	0	0.128028
YDR260C	SWM1	Mitosis	0	0.10997
YJL074C	SMC3	DNA replication & repair	0	0.104926
YHR191C	CTF8	DNA replication & repair	0	0.110713
YPL124W	SPC29	DNA replication & repair	0	0.144394
YJR076C	CDC11	Cytokinesis	0	0.127268
YLR381W	CTF3	DNA replication & repair	0	0.129803
YMR198W	CIK1	DNA replication & repair	0	0.132519
YGR099W	TEL2		0	0.101966
YBL039C	URA7		0	0.11297
YGL116W	CDC20	DNA replication & repair	0	0.154378
YNR016C	ACC1	DNA replication & repair	0	0.12848
YGR022C			0	0.107148
YCR049C			0	0.123282
YBR071W			0	0.11297
YAL065C			0	0.116169
YDR364C	CDC40		0	0.10997
YNR016C	ACC1	DNA replication & repair	0	0.138241
YML064C	TEM1	DNA replication & repair	0	0.142812
YPL008W	CHL1	DNA replication & repair	0	0.103561

YFL037W	TUB2	DNA replication & repair	0	0.213751
YOR052C	TMC1		0	0.10997
YDR254W	CHL4	DNA replication & repair	0	0.129803
YGL049C	TIF4632		0	0.11297
YIR010W	DSN1	DNA replication & repair	0	0.135329
YBR211C	AME1	DNA replication & repair	0	0.142812
YFL008W	SMC1	DNA replication & repair	0	0.144394
YOL104C	NDJ1		0	0.123282
YBR281C	DUG2		0	0.11297
YPL055C	LGE1	Transcription	0	0.101966
YOR249C	APC5	DNA replication & repair	0	0.107148
YDR318W	MCM21	DNA replication & repair	0	0.125897
YOL076W	MDM20	DNA replication & repair	0	0.149328
YMR076C	PDS5	DNA replication & repair	0	0.139737
YDR113C	PDS1	DNA replication & repair	0	0.113821
YAL068C	PAU8		0	0.10997
YER154W	OXA1	Mitochondria	0	0.104486
YLR350W	ORM2		0	0.112248
YFL034C-B	MOB2		0	0.116169
YFL034C-B	MOB2		0	0.123282
YNL288W	CAF40		0	0.116169
YDL231C	BRE4		0	0.116169
YGR188C	BUB1	DNA replication & repair	0	0.129803
YCR088W	ABP1		0	0.116169
YGR206W	MVB12		0	0.127268
YMR055C	BUB2	DNA replication & repair	0	0.142812
YJR112W	NNF1	DNA replication & repair	0	0.112248
YJL013C	MAD3	DNA replication & repair	0	0.109214
YLR247C	IRC20		0	0.116169
YOR195W	SLK19	DNA replication & repair	0	0.210356
YPL017C	IRC15	DNA replication & repair	0	0.129803
YBR156C	SLI15	DNA replication & repair	0	0.14126
YDL139C	SCM3	DNA replication & repair	0	0.127178
YER147C	SCC4	DNA replication & repair	0	0.151039
YER147C	SCC4	DNA replication & repair	0	0.109214
YDR180W	SCC2	DNA replication & repair	0	0.139737
YKL047W	ANR2		0	0.119596
YBL052C	SAS3		0	0.107148
YMR124W	EPO1	Cell polarity	0	0.101966
YPL028W	ERG10	Glycosylation & Protein folding	0	0.138767
YCR002C	CDC10	Cytokinesis	0	0.10997
YBR047W	FMP23		0	0.119596
YLR371W	ROM2	Glycosylation & Protein folding	0	0.13212
YDL069C	CBS1	Mitochondria	0	0.109214
YGR044C	RME1		0	0.107148
YGR140W	CBF2	DNA replication & repair	0	0.112248

YOR048C	RAT1	Transcription	0	0.119596
YBR107C	IML3	DNA replication & repair	0	0.146007
YJR076C	CDC11	Cytokinesis	0	0.116169

References

Golemis E. A., Serebriiskii I., Law S. F. (1999) The yeast two-hybrid system: criteria for detecting physiologically significant protein-protein interactions. *Curr. Issues Mol. Biol.* **1**, 31–45.

Khmelinskii A., Blaszczak E., Pantazopoulou M., Fischer B., Omnus D. J., Dez G. Le, Brossard A., et al. (2014) Protein quality control at the inner nuclear membrane. *Nature* **516**, 410–413.

Mumberg D., Muller R., Funk M., Müller R., Funk M. (1994) Regulatable promoters of Saccharomyces cerevisiae: comparison of transcriptional activity and their use for heterologous expression. *Nucleic Acids Res.* **22**, 5767–8.

Petroi D., Popova B., Taheri-Talesh N., Irniger S., Shahpasandzadeh H., Zweckstetter M., Outeiro T. F., Braus G. H. (2012) Aggregate clearance of alpha-synuclein in Saccharomyces cerevisiae depends more on autophagosome and vacuole function than on the proteasome. *J Biol Chem* **287**, 27567–27579.

Popova B., Galka D., Häffner N., Wang D., Schmitt K., Valerius O., Knop M., Braus G. H. (2021a) α-Synuclein Decreases the Abundance of Proteasome Subunits and Alters Ubiquitin Conjugates in Yeast. *Cells 2021, Vol. 10, Page 2229* **10**, 2229.

Popova B., Wang D., Pätz C., Akkermann D., Lázaro D. F., Galka D., Kolog Gulko M., et al. (2021b) DEAD-box RNA helicase Dbp4/DDX10 is an enhancer of α-synuclein toxicity and oligomerization. *PLOS Genet.* **17**, e1009407.

Saeki Y., Toh-e A., Kudo T., Kawamura H., Tanaka K. (2009) Multiple Proteasome-Interacting Proteins Assist the Assembly of the Yeast 19S Regulatory Particle. *Cell* **137**, 900–913

Shahpasandzadeh H., Popova B., Kleinknecht A., Fraser P. E., Outeiro T. F., Braus G. H. (2014) Interplay between sumoylation and phosphorylation for protection against alpha-synuclein inclusions. *J Biol Chem* **289**, 31224–31240.

Sikorski R. S., Hieter P. (1989) A system of shuttle vectors and yeast host strains designed for efficient manipulation of DNA in Saccharomyces cerevisiae. *Genetics* **122**, 19–27.

Tenreiro S., Rosado-Ramos R., Gerhardt E., Favretto F., Magalhães F., Popova B., Becker S., Zweckstetter M., Braus G. H., Outeiro T. F. (2016) Yeast reveals similar molecular mechanisms underlying alpha- and beta-synuclein toxicity. *Hum. Mol. Genet.* **25**, 275–290.
